# Neocortical long-range inhibition promotes cortical synchrony and sleep

**DOI:** 10.1038/s41586-026-10876-y

**Published:** 2026-09-09

**Authors:** Jacob M. Ratliff, Geoffrey Terral, Arenski Vazquez, Stefano Lutzu, Arena Manning, Nelson Perez-Catalan, Gabriela Neubert da Silva, Soyoun Kim, Julie Mota, Matt Mallory, Bianca Stith, Charu Ramakrishnan, Gianna Mattessich, Lief E. Fenno, Tanya Daigle, David A. Stafford, Hongkui Zeng, Bosiljka Tasic, Staci Sorensen, Karl Deisseroth, John Ngai, Thomas S. Kilduff, Lucas Sjulson, Stephanie Rudolph, Renata Batista-Brito

**Affiliations:** 1 Dominick P. Purpura Department of Neuroscience, Albert Einstein College of Medicine, New York, NY USA; 2 Allen Institute for Brain Science, Seattle, WA USA; 3 Department of Bioengineering, Stanford University, Stanford, CA USA; 4 Department of Neuroscience, University of Texas at Austin, Austin, TX USA; 5 Department of Psychiatry and Behavioral Sciences, University of Texas at Austin, Austin, TX USA; 6 Department of Molecular and Cellular Biology, University of California, Berkeley, Berkeley, CA USA; 7 Department of Psychiatry and Behavioral Sciences, Stanford University, Stanford, CA USA; 8 Howard Hughes Medical Institute, Stanford University, Stanford, CA USA; 9 National Institute of Neurological Disorders and Stroke, National Institutes of Health, Bethesda, MD USA; 10 Biosciences Division, SRI International, Menlo Park, CA USA; 11 Department of Psychiatry and Behavioral Sciences, Albert Einstein College of Medicine, New York, NY USA; 12 Department of Genetics, Albert Einstein College of Medicine, New York, NY USA; 13 Present Address: UCL Queen Square Institute of Neurology, University College London, London, UK; 14 Present Address: Nash Family Department of Neuroscience, Icahn School of Medicine at Mount Sinai, New York, NY USA

**Keywords:** Slow-wave sleep, Neural circuits

## Abstract

Sleep and wakefulness are associated with distinct cortical patterns of rhythmic activity^1^. During low-arousal states such as slow-wave sleep, synchronous low-frequency rhythms dominate activity across widespread cortical regions. Although inhibitory neurons are increasingly recognized as key regulators of cortical state^2–4^, the circuit mechanisms that coordinate synchronized activity across local and distant neocortical networks in vivo remain poorly understood. Here we show in mice that cells co-expressing somatostatin (Sst) and chondrolectin (Chodl)—which constitute a sparse and genetically distinct class of neocortical GABAergic inhibitory neurons—are selectively active during low-arousal states and mostly silent during periods of high arousal. In contrast to most neocortical inhibitory neurons, Sst-Chodl cells, despite being extremely sparse, exert widespread influence across the neocortex, through long-range axons that target multiple regions simultaneously. Selective activation of Sst-Chodl cells is sufficient to promote the multi-region cortical synchronization that is characteristic of low-arousal states and to induce sleep. Together, these findings show that long-range Sst-Chodl inhibitory neurons not only track behavioural state, but can also actively promote synchronized cortical activity and sleep behaviour, highlighting that cortical circuits have a key role in sleep regulation, alongside established subcortical mechanisms.

## Abstract (web summary)

In mice, a sparse population of sleep-active long-range inhibitory neurons in the neocortex promote widespread cortical synchronization and sleep, revealing a cortical mechanism that contributes to the regulation of sleep.

## Main

Sleep and rest are essential for mammalian physiology. Their disruption leads to widespread physiological and cognitive impairments and is linked to a wide range of disorders, including insomnia, sleep apnoea, narcolepsy, depression, attention deficit hyperactivity disorder and schizophrenia, and can even lead to death^5^. Transitions between alertness and low-arousal states, such as quiet wakefulness and sleep, occur on a timescale of seconds and are accompanied by pronounced changes in neocortical activity patterns (oscillatory activity or cortical states)^1^. Low-arousal states, including slow-wave sleep (SWS) and quiet immobility, are characterized by synchronous low-frequency cortical fluctuations and coordinated spiking activity, often referred to as synchronized cortical states. These alternate with desynchronized states associated with periods of high arousal and active behaviour, during which low-frequency cortical activity is suppressed^1^. GABAergic inhibitory neurons (INs) have been implicated as key regulators of arousal-dependent neocortical activity^4^. Although fast-spiking parvalbumin-expressing INs have been shown to promote desynchronized states—which dominate periods of alertness—by enhancing gamma-band activity^2,3^, much less is known about the identity and function of IN populations that regulate the synchronized cortical states of low-arousal periods.

The diversity of cortical INs has been recognized for nearly a century^6^, but assigning specific functions to individual classes of IN has proved challenging, in part because established IN classes are themselves highly heterogeneous^7^. For example, somatostatin (Sst)-expressing INs, which are often treated as a monolithic group, can be subdivided into more than ten subclasses with distinct morpho-electric and transcriptomic properties^7^. In this study, we focus on a transcriptionally homogeneous and evolutionarily conserved subtype of Sst-expressing INs characterized by co-expression of Sst, chondrolectin (Chodl), neuronal nitric oxide synthase (Nos1) and the neurokinin-1 receptor (Tacr1). These cells, hereafter referred to as Sst-Chodl cells, correspond to the Nos1 (or nNOS for immunoreactivity)-expressing cortical IN population described in previous studies^7–9^. Sst-Chodl cells are GABAergic INs that differ considerably from canonical, locally projecting neocortical interneurons in that they have long-range projection axons that can extend across millimetres in the mouse cortex and centimetres in larger brains^10,11^. Despite their extreme sparsity—comprising less than 1% of cortical GABAergic neurons^9^—Sst-Chodl cells are highly conserved across vertebrate species, from salamanders to humans^12^. Previous ex vivo studies have suggested that putative Sst-Chodl cells are likely to be active during SWS^13–15^, raising the possibility that they contribute to synchronized states. However, their activity and functional roles in vivo have remained difficult to investigate owing to a lack of tools for their specific targeting.

Here, using an intersectional genetic strategy to selectively target Sst-Chodl cells, we show that these neurons form dense regional and inter-areal intracortical connections and are preferentially active during periods of cortical synchrony, including quiet wakefulness and SWS. Activation of Sst-Chodl cells promotes synchronized cortical states and sleep-related behaviours. Together, these findings identify Sst-Chodl cells as a cortical-circuit element that links neocortical synchrony to sleep regulation, consistent with emerging evidence that cortical activity contributes directly to the control of sleep^16–25^.

## Sst-Chodl cells are long-range projecting INs

**Fig. 1:**
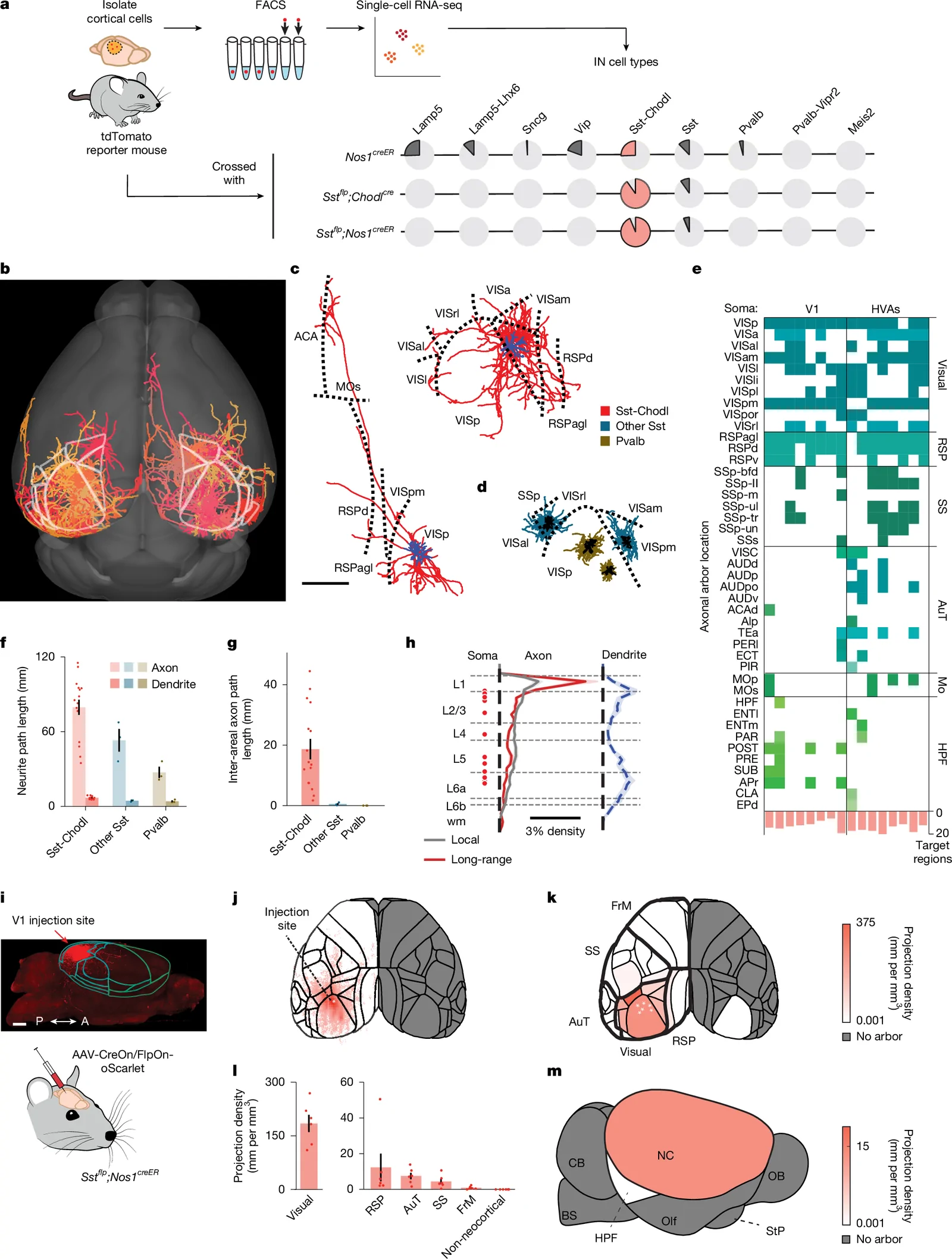
Sst-Chodl cells are long-range INs with extensive intracortical projections. **a**, Experimental strategy for transcriptomic profiles and quantification of IN cell types obtained from single-cell RNA sequencing (RNA-seq) of labelled neurons isolated by fluorescence-activated cell sorting (FACS) from tdTomato reporter mice crossed with *Nos1*^*creER*^, *Sst*^*flp*^*;Chodl*^*cre*^ or *Sst*^*flp*^*;Nos1*^*creER*^ mouse lines (data from ref. ^52^). These approaches selectively and robustly label Sst-Chodl cells. **b**, Single-cell reconstructions of Sst-Chodl cells in V1 and higher visual areas (HVAs). **c**, Example reconstructions of two Sst-Chodl cells (axons in red; dendrites in dark blue) illustrating dense local arborization together with long-range projections spanning millimetres. Dashed lines indicate area borders. Scale bar, 1 mm. **d**, Reconstructions of other Sst neurons (non-Chodl; blue, dendrites in black) and Pvalb neurons (khaki; dendrites in black), which show predominantly local axons with minimal inter-areal spread. These comparisons reveal that Sst-Chodl cells, unlike other inhibitory cell types, routinely project far beyond their home cortical area. **e**, Axonal arborization matrix summarizing downstream cortical regions targeted by individual Sst-Chodl cells (columns); coloured boxes indicate the presence of axon in the region. Bottom, number of downstream regions innervated by each reconstructed cell. **f**, Comparison of axon and dendrite length across Sst-Chodl, other Sst and Pvalb cells. **g**, Axon length for long-range axons (located outside of visual cortical areas) (*n* = 16 Sst-Chodl cells from 3 mice, 3 other Sst cells from 3 mice, and 3 Pvalb cells from 2 mice). **h**, Layer distribution of axons, dendrite density and soma positions for reconstructed neurons. wm, white matter. These analyses show that Sst-Chodl cells have widespread intracortical arbors, with long-range axons travelling preferentially through superficial layers. **i**, Strategy for bulk arbor reconstruction after injection of AAV-CreOn/FlpOn-oScarlet into V1 of *Sst*^*flp*^*;Nos1*^*creER*^ mice, with example sagittal maximum-intensity projection of labelled fibres. Scale bar, 1 mm. A, anterior; P, posterior. **j**, Thin-section reconstructions showing projection patterns in an example brain. **k**, Mean projection density across the brain, with inferred injection sites marked with asterisks. **l**, Quantification of projection densities within the visual cortex and across other brain areas. **m**, Brain-wide distribution of V1 Sst-Chodl projections (*n* = 6). These population-level datasets confirm that Sst-Chodl cells send dense local axons within visual areas and widespread ipsilateral projections across most of the neocortex, with sparse extensions into para-hippocampal regions. Data are mean ± s.e.m. (bars and shading). See Methods for abbreviations of brain regions. The mouse cartoons in **a**,**i** are adapted with permission from ref. ^53^, Elsevier. Source data

**Extended Data Fig. 1:**
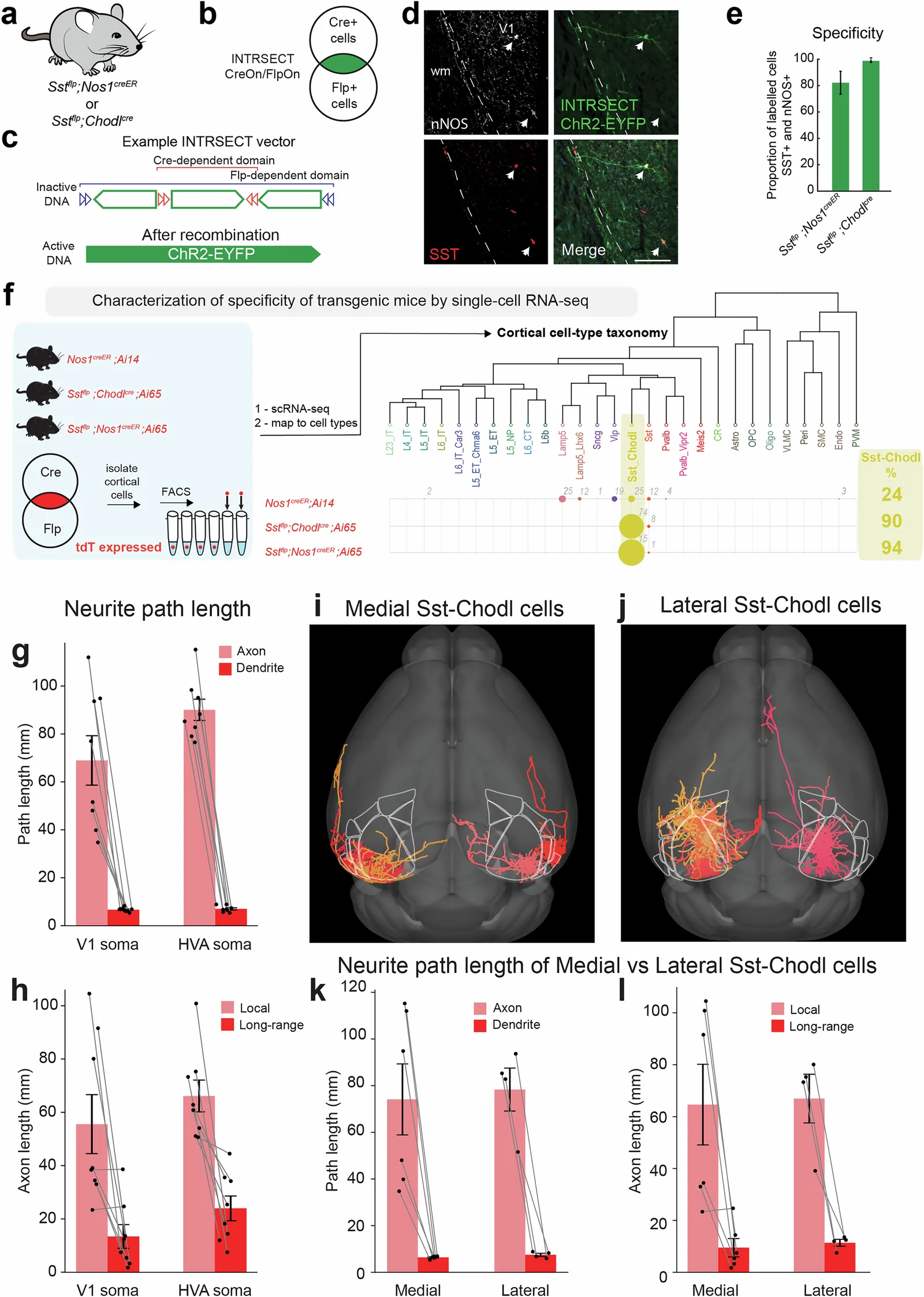
Intersectional targeting and long-range projections of Sst-Chodl cells. **a)** Mouse lines used for intersectional targeting of Sst-Chodl cells. **b)** Venn diagram showing the populations labelled by CreOn/FlpOn INTRSECT vector. **c)** Example INTRSECT construct illustrating that fluorophore expression occurs only after both Cre and Flp recombination. This ensures highly selective access to Sst-Chodl cells. **d)** Histology from *Sst*^*flp*^*;Nos1*^*creER*^ mice showing ChR2-EYFP expression in Sst-Chodl cells in V1; arrows indicate cells co-expressing SST and nNOS. Long-range axonal segments travelling through white matter (wm) are visible. Scale, 150 µm. These examples show the specificity of labelling and the presence of projecting axons originating from targeted cells. **e)** Proportion of cells expressing GCaMP, SST, and nNOS in *Sst*^*flp*^*;Nos1*^*creER*^ mice (62 cells from 4 mice) and *Sst*^*flp*^*;Chodl*^*cre*^ mice (30 cells from 4 mice), showing the specificity of the labelling strategies for studying Sst-Chodl cells. **f)** Cortical cell-type taxonomy obtained from single-cell RNA sequencing of labelled neurons from three transgenic lines (*Nos1*^*creER*^, *Sst*^*flp*^*;Chodl*^*cre*^ and *Sst*^*flp*^*;Nos1*^*creER*^) crossed with the tdTomato reporter line (data from^52^). Numbers indicate cell count. This transcriptomic analysis further verifies that the intersectional strategies provide enriched labelling of Sst-Chodl cells. **g)** Axonal and dendritic path length of reconstructed Sst-Chodl cells originating in primary visual cortex (V1) and higher visual areas (HVA). These measurements show that Sst-Chodl cells in V1 and HVAs have broadly similar arborization magnitudes. **h)** Same as **g**, separated into local axon (within visual areas) versus long-range axon (outside visual areas). Both V1- and HVA-derived cells exhibit substantial long-range projections, indicating that long-range targeting is a shared property across visual cortical Sst-Chodl populations. **i**,**j)** Example reconstructions of Sst-Chodl cells with somata positioned in medial (**i**) versus lateral (**j**) regions of visual cortex. **k**,**l)** Quantification of total axonal and dendritic path lengths (**k**) and local versus long-range axon length (**l**) for medial vs. lateral Sst-Chodl cells. These comparisons show that cell-body location along the medial–lateral axis does not substantially alter overall arbor size or long-range projection extent. (N = 16 Sst-Chodl cells from 3 mice) Data are means; bars indicate s.e.m. The mouse cartoons in **a**,**f** are adapted with permission from ref. ^53^, Cell Press. Source data

We used intersectional genetics to selectively target Sst-Chodl cells (Fig. 1a and Extended Data Fig. 1). To evaluate the extent of their projections, we used the *Sst*^*flp*^;*Chodl*^*cre*^ intersection to sparsely label individual neurons, enabling single-cell anatomical reconstruction of individual Sst-Chodl cells across the neocortex. We reconstructed the full morphology of 16 Sst-Chodl cells across all layers in the primary visual cortex (V1) and higher visual areas (Fig. 1b), and compared their projection pattern with those of parvalbumin (Pvalb) and non-Chodl Sst (other Sst cells) reconstructed cells (Fig. 1c–g). Whereas Pvalb and other Sst cells had local arborization, rarely crossing area boundaries, we found that Sst-Chodl cells had both dense local arborization within visual areas and long-range axons travelling millimetres from the soma across broad neocortical areas (Fig. 1b–g and Supplementary Tables 1 and 2). The projections of Sst-Chodl cells were exclusively intracortical and ipsilateral, broadly targeting nearly all cortical areas, with the notable exception of far-anterior (for example, frontal pole and prelimbic area) and ventral (for example, gustatory area) regions (Fig. 1e). Of note, projection patterns were highly similar between Sst-Chodl cells that originated in V1 and those that originated in higher visual areas (Extended Data Fig. 1g,h and Supplementary Tables 1 and 2), and between medial and lateral Sst-Chodl cells (Extended Data Fig. 1i–l and Supplementary Tables 1 and 2). We encountered local and regional projections across all layers, whereas the long-range projections were preferentially found in upper layers (Fig. 1h).

**Extended Data Fig. 2:**
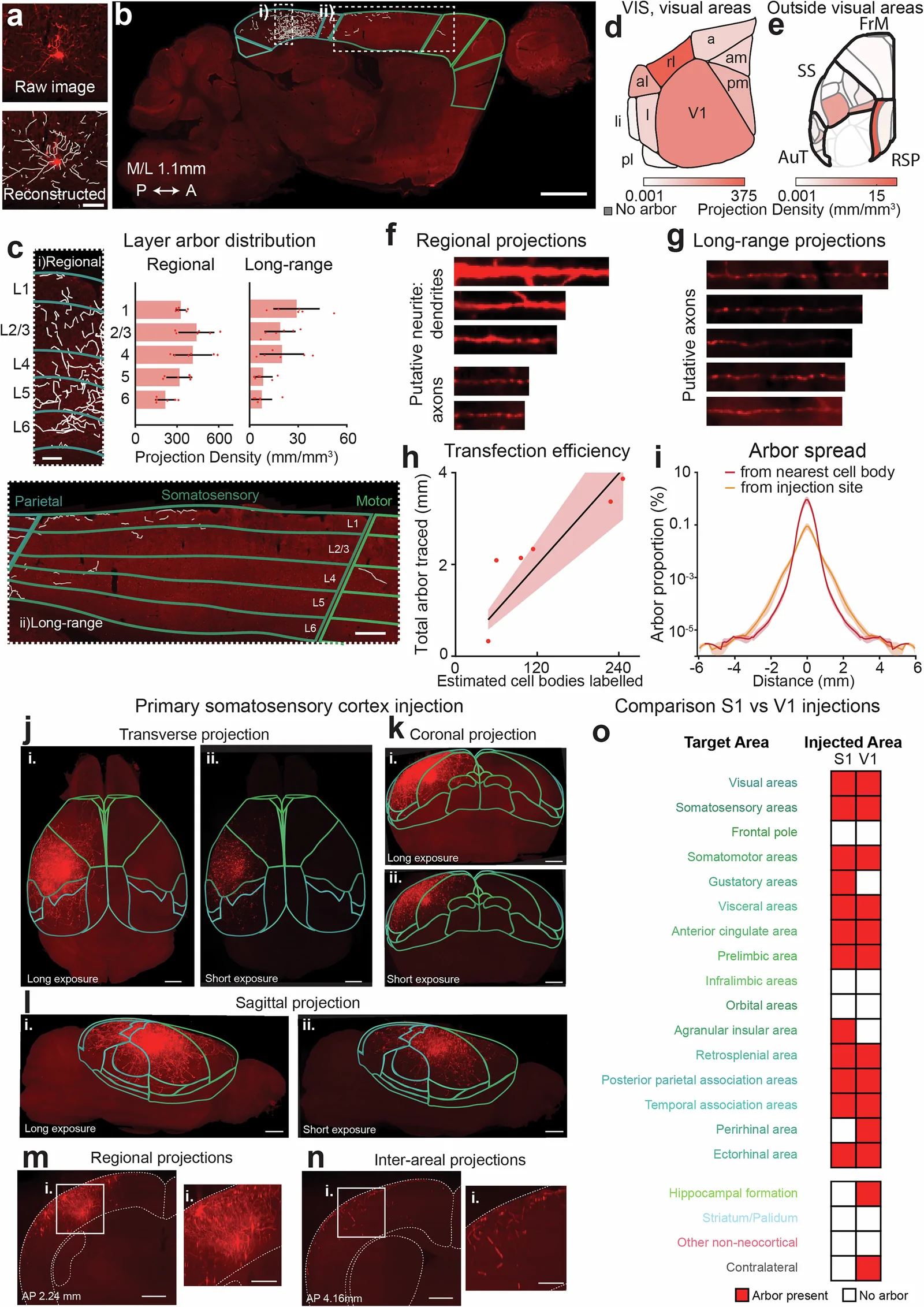
Projection patterns of Sst-Chodl cells originating in visual and somatosensory cortical areas. **a)** Example thin-section reconstruction of a Sst-Chodl cell arbor. Scale: 100 μm. **b)** Example thin-section showing both regional and long-range oScarlet-labelled axons from V1 Sst-Chodl cells. Scale, 1 mm. This reconstruction illustrates the coexistence of dense local arbors and extensive inter-areal projections. **c)** Insets from (**b**) with quantifying regional and inter-areal arbor density. Scale, 100 and 200 µm, respectively. These measurements show that visual areas receive the densest innervation, with substantial additional arbor distributed across other cortical regions. **d)** Projection density across visual cortical subregions averaged across mice, demonstrating consistent local arbor patterns across individuals. **e)** Quantification of long-range projections targeting neocortical areas outside the visual cortex, confirming broad ipsilateral inter-areal connectivity. **f)** Putative dendrites and axons near the injection site in V1. **g)** Putative axons lacking dendrites far from injection site (outside visual areas), consistent with distal long-range projections. **h)** Regression relating transfection strength to total arbor traced, indicating that measured arbor size scales with expression level. **i)** Spread of labelled arbor from the inferred injection site (orange) and from the nearest reconstructed soma (dark red), travelling multiple millimetres, underscoring the long-range nature of Sst-Chodl projections. N = 6 mice. Data are means; shading and bars indicate s.e.m. **j)** Dorsal view of a cleared whole brain showing oScarlet-labelled S1 Sst-Chodl cell arbors with (i) long and (ii) short exposure. **k**,**l)** Same dataset shown from anterior (**k**) and lateral (**l**) perspectives. Scale for **j**–**l**, 1 mm. These views reveal dense local labelling in S1 and prominent long-range axons projecting across extensive ipsilateral cortical territory. **m)** Coronal section near the injection site showing dense local arborization. Scale, 0.5 mm; inset, 0.25 mm. **n)** Coronal section far from the injection site, where labelled fibres appear without nearby somata, demonstrating distal long-range projections from S1 Sst-Chodl cells. Insets correspond to zoomed regions noted in (i). **o)** Comparison of targeted cortical areas following injections into S1 vs V1, showing that Sst-Chodl cells originating from each region innervate surrounding neocortical regions but also send inter-areal projections (N = 3 mice for S1 injections, N = 6 mice for V1 injections). See Methods for abbreviations of brain regions. Source data

We next quantified the density of the Sst-Chodl projections from V1 to other brain regions by injecting the intersectional AAV8-Ef1a-CreOn/FlpOn-oScarlet vector into V1 of *Sst*^*flp*^ and *Nos1*^*creER*^ double-transgenic mice (Extended Data Fig. 2), driving robust cytosolic labelling of Sst-Chodl cells (Fig. 1i). At the population level, we annotated the oScarlet-labelled arbor of Sst-Chodl cells across the brain and found broadly ipsilateral neocortical projections (with small contralateral V1 projections), with dense innervation of visual areas (200–600 mm of arbor per mm^3^ of tissue; Fig. 1j–l and Extended Data Fig. 2a–i) and extensive inter-areal projections that targeted most of the neocortex, including retrosplenial, auditory, somatosensory and frontal motor areas (Fig. 1l). Notably, only a limited set of far-anterior and dorsal neocortical regions lacked detectable innervation (agranular insular area, frontal pole, gustatory areas, infralimbic area and orbital area). Consistent with the single-cell reconstruction data (Fig. 1b–e), we also observed small but reproducible projections from V1 Sst-Chodl cells beyond the neocortex, targeting ipsilateral para-hippocampal structures, including the subiculum and the entorhinal cortex (Fig. 1m and Supplementary Table 3).

Using whole-brain tissue clearing and light-sheet imaging, we compared Sst-Chodl arborization across mice labelled in V1 or in the primary somatosensory cortex (S1) (Extended Data Fig. 2j–o and Supplementary Video 1). Similarly to cells in V1, S1 Sst-Chodl cells exhibited dense local arbors across layers and long-range ipsilateral projections confined to the neocortex and preferentially travelling through superficial layers (Extended Data Fig. 2j–n). Whereas the inter-areal projections from V1 Sst-Chodl cells extensively targeted retrosplenial areas, the long-range projections of S1 Sst-Chodl cells more densely innervated areas surrounding the injection site, such as frontal, motor and visual areas (Extended Data Fig. 2j–o).

Together, these data show that Sst-Chodl cells originating from distinct neocortical areas share a common feature: they are long-range projecting neurons that broadly target the ipsilateral neocortex.

## Inputs to Sst-Chodl cells from diverse cell classes

**Extended Data Fig. 3:**
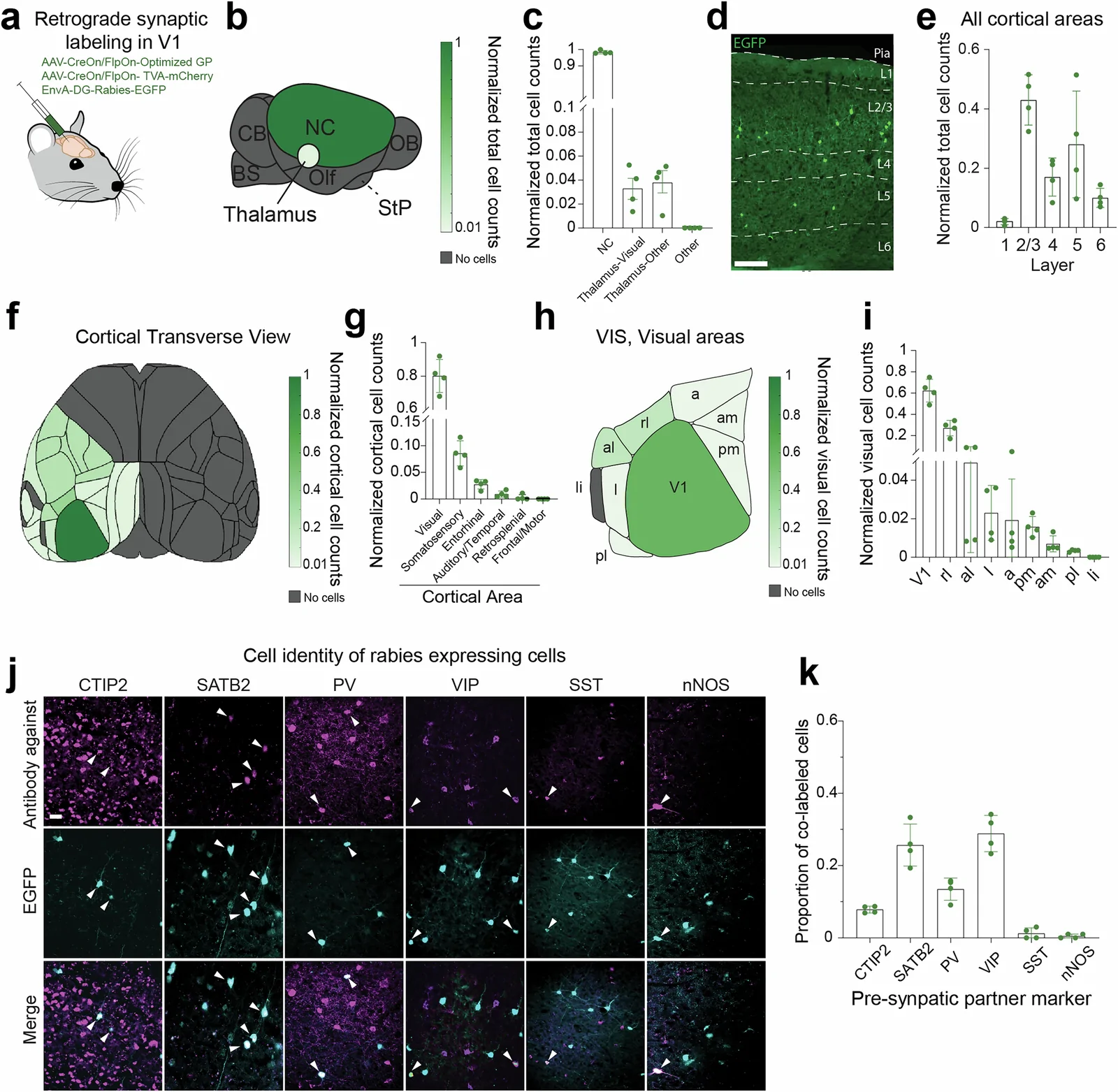
Widespread intracortical excitatory and inhibitory inputs to Sst-Chodl cells. **a)** Schematic of the monosynaptic rabies-based retrograde tracing strategy using intersectional genetics to identify presynaptic partners of Sst-Chodl cells. This approach enables selective labelling of neurons providing direct input to Sst-Chodl cells. **b)** Distribution of rabies-tagged presynaptic neurons across major brain structures. Only a small minority of labelled cells were found in the thalamus (28 out of 527 total cells), indicating that Sst-Chodl cells receive relatively sparse thalamic inputs. **c)** Cell counts across brain regions normalized to total labelled cells. “Thalamus-Visual” includes the dorsal lateral geniculate complex; “Thalamus-Other” includes dorsal thalamus and medial geniculate regions. **d)** Example coronal section showing rabies-expressing presynaptic neurons (EGFP) across layers of V1. Scale, 100 µm. **e)** Quantification of laminar distribution within the neocortex, expressed as a proportion of labelled neocortical cells. Inputs arise predominantly from intermediate layers (layers 2–5). **f**,**g)** Distribution (**f**) and quantification (**g**) of presynaptic neurons across neocortical areas, normalized to the total neocortical presynaptic pool (**f**, transverse view; **g**, quantification). Labelled neurons were almost exclusively ipsilateral and most abundant in V1. **h**,**i)** Distribution (**h**; transverse view) and quantification (**i**) of presynaptic neurons across visual cortical areas, showing that V1 provides the densest inputs within the visual system. **j)** Immunohistochemical examples of presynaptic cells co-expressing rabies and major neuronal subtype markers, including glutamatergic markers CTIP2 (putative pyramidal tract) and SATB2 (intratelencephalic), as well as GABAergic markers PV, VIP, and SST. Scale, 20 µm; white arrows indicate co-labelled cells. **k)** Proportion of presynaptic neurons co-expressing each marker. These analyses show that Sst-Chodl cells receive input from all major cortical excitatory and inhibitory populations probed, with SATB2^+^ and VIP^+^ cells being the most prominent contributors and SST^+^nNOS^+^ neurons the least frequent. Together, these data demonstrate that Sst-Chodl cells integrate a broad array of intracortical inputs, with minimal thalamic contribution and substantial excitatory and inhibitory presynaptic diversity. N = 4 mice. Data are means; bars indicate s.e.m. See Methods for abbreviations of brain regions. The mouse cartoon in **a** is adapted with permission from ref. ^53^, Cell Press. Source data

To identify the presynaptic partners of Sst-Chodl cells, we used an intersectional genetic, monosynaptic retrograde rabies tracing strategy from Sst-Chodl cells in V1 (Extended Data Fig. 3a and Supplementary Table 4). Presynaptic neurons were located almost exclusively within neocortical areas, with only a small fraction detected in the thalamus (less than 4% of all labelled cells; Extended Data Fig. 3b,c). Within the neocortex, labelled neurons were restricted to the ipsilateral hemisphere, distributed mainly across intermediate layers (2–5) and most densely represented in V1 (Extended Data Fig. 3d–i). Immunohistochemical analyses revealed that presynaptic neurons comprised both glutamatergic (CTIP2^+^, putative pyramidal tract; SATB2^+^, putative intratelencephalic) and GABAergic (PV^+^, VIP^+^ and SST^+^) populations, with the majority co-expressing SATB2 and VIP, and the fewest co-expressing SST and nNOS (Extended Data Fig. 3j,k).

Together, these data indicate that Sst-Chodl cells integrate a broad diversity of regional excitatory and inhibitory inputs from all of the major glutamatergic and GABAergic classes that we probed.

## Sst-Chodl cells are active in periods of low arousal

**Fig. 2:**
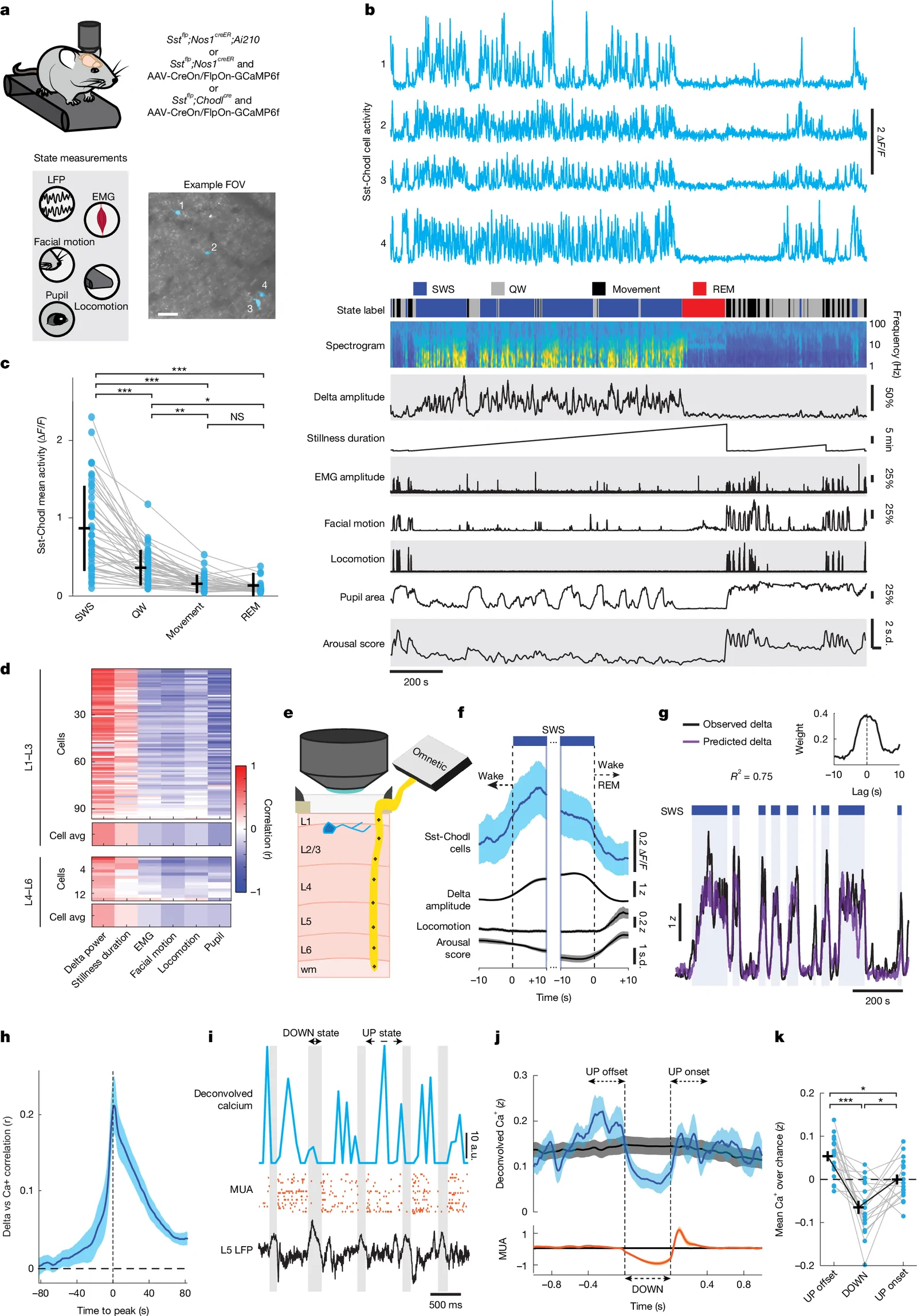
Sst-Chodl cells are strongly active during low-arousal, synchronized cortical states. **a**, Strategy for expressing GCaMP in Sst-Chodl cells and for simultaneous two-photon imaging in V1, LFP recording in contralateral V1 and behavioural state monitoring in head-fixed mice. Example field of view (FOV) showing four labelled Sst-Chodl cells (regions of interest in blue). Scale bar, 100 µm. **b**, Example traces from the four imaged Sst-Chodl cells in **a**, alongside behavioural and physiological measurements of sleep and wake states. These measurements show that Sst-Chodl cell activity increases when the cortex enters synchronous states such as SWS and QW. **c**, Mean activity of layer 1–3 Sst-Chodl cells across behavioural states (ANOVA, *P* < 0.001; two-sided Bonferroni post-hoc tests: SWS–QW, ***P < 0.001; SWS–movement, ***P < 0.001; SWS–REM, ***P < 0.001; QW–movement, **P = 0.001; QW–REM, *P = 0.010; movement–REM, *P* = 0.990; NS, not significant). This shows that Sst-Chodl cells in superficial layers are most active during SWS and QW and suppressed during movement and REM. **d**, Correlation between Sst-Chodl cell activity and state metrics for cells in superficial layers (recorded through cranial windows; layers 1–3) and in deeper cell layers (recorded through microprism; layers 4–6). Sst-Chodl cell activity across all layers was positively correlated with markers of low arousal and negatively correlated with high-arousal signals. Layers 1–3: *n* = 15 mice (11 *Sst*^*flp*^*;Nos1*^*creER*^ and 4 *Sst*^*flp*^*;Chodl*^*cre*^), *n* = 97 cells (76 *Sst*^*flp*^*;Nos1*^*creER*^ and 21 *Sst*^*flp*^*;Chodl*^*cre*^); layers 4–6: *n* = 5 mice (*Sst*^*flp*^*;Chodl*^*cre*^), *n* = 14 cells. avg, average. L1–L6, layers 1–6). **e**, Schematic of simultaneous two-photon imaging and LFP recordings using a flexible 32-channel silicon probe beneath the cranial window.** f**, Mean Sst-Chodl cell activity, delta power, locomotion and arousal aligned to transitions into and out of SWS. Sst-Chodl activity increased with high delta power and decreased during desynchronized states. **g**, Cross-validated linear regression model predicting delta amplitude from deconvolved Sst-Chodl calcium signals, showing that activity reflects ongoing delta fluctuations rather than predicting future changes. **h**, Cross-correlation between delta amplitude and Sst-Chodl cell calcium activity, indicating tight temporal coupling. **i**, Example recordings of deconvolved Sst-Chodl cell activity (a.u., arbitrary units), multi-unit activity (MUA) and layer 5 LFP. DOWN states were detected from MUA and aligned to MUA and LFP signals.** j**, Average peri-stimulus time histogram of calcium activity across Sst-Chodl cells (blue) and MUA (orange) aligned to DOWN state onset; chance levels are shown in black. **k**, Average calcium activity from 400 ms before DOWN states (UP offset), during DOWN states (DOWN) and 400 ms after DOWN states (UP onset). Sst-Chodl cells show increased activity before DOWN states (ANOVA, *P* < 0.001; two-sided Bonferroni post-hoc tests: UP offset–DOWN, ***P < 0.001; DOWN–UP onset, *P = 0.017; UP offset–UP onset, *P = 0.036), consistent with engagement at the termination of UP states rather than during the silent DOWN phase. **f**–**k** include data from two-photon-probe recordings shown in **e**. *n* = 4 mice (*Sst*^*flp*^*;Chodl*^*cre*^), *n* = 17 cells. ***P < 0.001, **P < 0.01, *P < 0.05; NS, not significant. Data are mean ± s.e.m. (bars and shading). The mouse cartoon in **a** is adapted with permission from ref. ^53^, Elsevier. Source data

**Extended Data Fig. 4:**
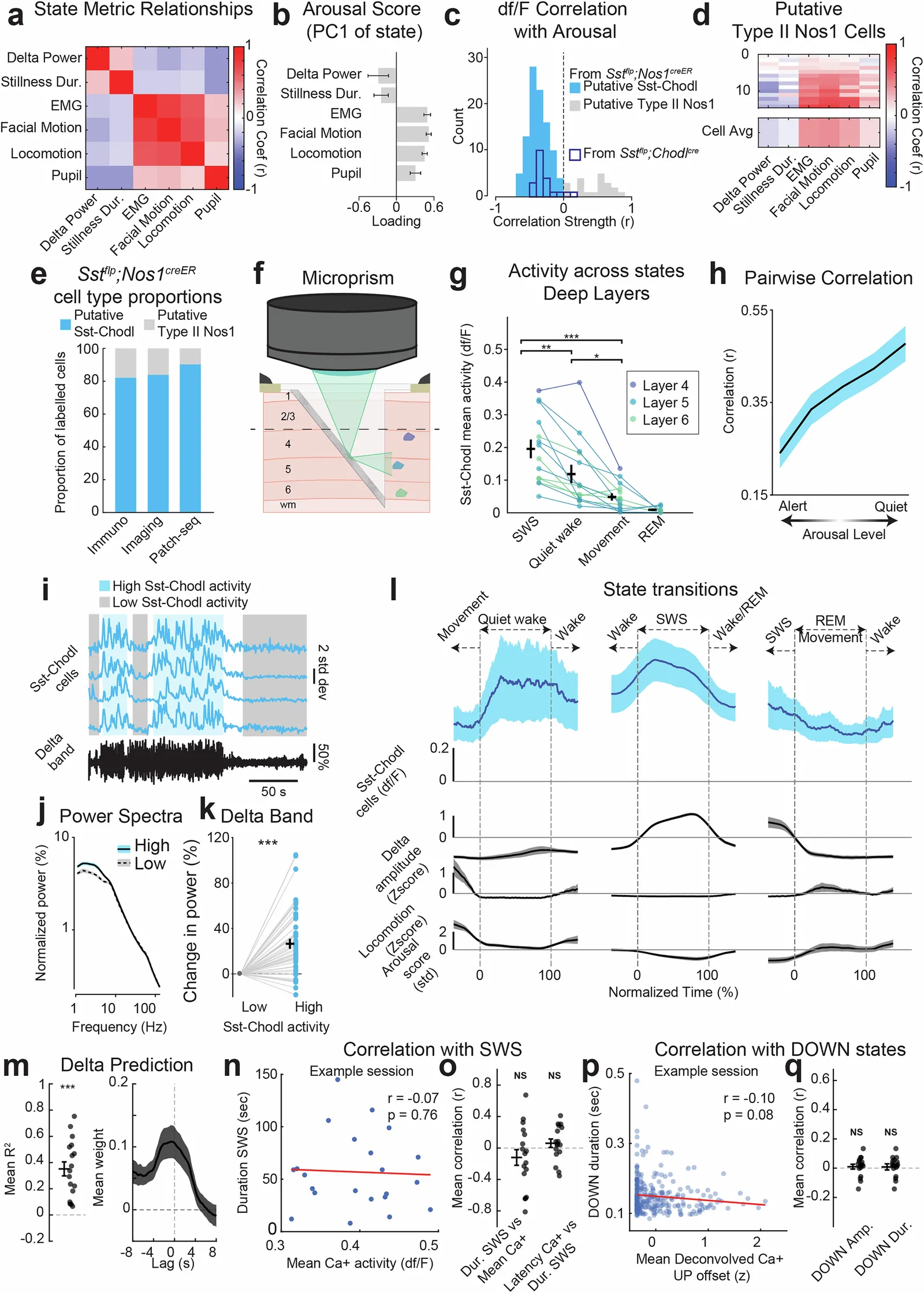
Sst-Chodl population activity tracks low-arousal synchronized states. **a)** Average correlation between state measurements showing consistent relationship between the metrics. **b)** Consistent PC1 loadings in PCA of state measurements across mice. **c)** Distribution of correlation coefficients between imaged cell Δ*F*/*F* and arousal score. Cells from *Sst*^*flp*^*;Nos1*^*creER*^ mice (full) and from *Sst*^*flp*^*;Chodl*^*cre*^ mice (clear) are assigned by k-means clustering for each space of Δ*F*/*F* versus state metric correlation. **d)** Correlation of putative type II Nos1 cell activity with state metrics (n = 16 out of 111 recorded cells from 15 mice from layers 1 to 3), showing a small proportion of cells with opposite pattern of activity as compared to putative Sst-Chodl cells. **e)** Proportion of Sst-Chodl and putative Type II Nos1 cells with identity determined by immunohistochemistry (Immuno, n = 62 cells from 4 mice), imaging and activity correlation (Imaging, n = 90 cells from 11 mice), and by patch-seq in *Sst*^*flp*^*;Nos1*^*creER*^ mice crossed with *Ai65* mice from the Allen Brain Institute^8^ (Patch-seq, n = 87 cells). The small proportion of putative Type II Nos1 cells is consistent across our immunohistochemistry and imaging results and sequencing data. **f)** Schematic of Sst-Chodl cells recorded in deep layers (estimated cell position in layer 4, dark blue; layer 5, light blue; layer 6, light green) with a microprism implanted in V1. **g)** Mean activity of deep-layer recorded cells across states. ANOVA *P* < 0.001, post-hoc multiple comparisons: SWS-QW ** *P* = 0.002, SWS-Movement *** *P* < 0.001, QW-Movement * *P* = 0.016. N = 5 mice (from *Sst*^*flp*^*;Chodl*^*cre*^ mice x *Ai210* cross), n = 14 cells. Putative Type II Nos1 cells were not represented in the graph (2 out of 16 recorded cells). Similarly to Sst-Chodl cells recorded in superficial layers, deep-layer cells are mainly active during low-arousal states, such as SWS and QW. **h)** Mean pairwise correlation between cells based on arousal level (n = 42 pairs), showing consistent correlation activity across cells relative to arousal. **i)** Example recordings during periods of high and low Sst-Chodl cell activity. **j)** Power spectra during periods of high and low Sst-Chodl cell activity and **k)** quantification of delta-band power change between these periods, *P* < 0.001 n = 97 cells, 15 mice. These results show that Sst-Chodl cells are most active when cortical networks are highly synchronized. **l)** Mean Sst-Chodl cell activity, delta amplitude, locomotion, and arousal level (note multiple scales) around state transitions. Sst-Chodl cell activity increases as mice enter low-arousal states in parallel with rising delta power, and decreases when they transition to desynchronized states. **m)** Average linear regression model predicting delta amplitude from deconvolved Sst-Chodl calcium activity. Left: cross-validated R² from ordinary least-squares regression (*P* < 0.001, one-sided Wilcoxon test). Right: mean kernel weights ( ± 1 SD) across temporal lags. **n**,**o)** Relationship between SWS bout duration and Sst-Chodl cell activity. **n)** Example session showing correlations between bout duration and mean calcium activity (left: r = −0.07, P = 0.76; Spearman correlation). **o)** Mean correlation coefficients across cells (Duration SWS vs. Mean Ca^2+^, *P* = 0.266; Latency Ca+ vs. Duration SWS: *P* = 0.237; one-sided Wilcoxon test). These results indicate that Sst-Chodl activity does not predict the duration or onset timing of SWS bouts. **p**,**q)** Relationship between DOWN-state features and Sst-Chodl cell calcium activity. **p)** Example session showing correlations between DOWN duration and mean UP-offset calcium activity (right: r = −0.10, *P* = 0.08; Spearman correlation). **q)** Mean correlation coefficients across cells (DOWN amplitude vs. Ca^2+^: *P* = 0.332; DOWN duration vs. Ca^2+^: *P* = 0.586; Wilcoxon test). These analyses show that although Sst-Chodl cells increase activity prior to DOWN onset, their activity does not predict the amplitude or duration of the upcoming DOWN state. Data in **l**-**q** panels are from simultaneous two-photon and flexible-probe recordings (Fig. 2e–k), n = 17 cells, 4 mice. *** *P* < 0.001, ** *P *< 0.01, * *P* < 0.05; NS, not significant. Data are means; shading and bars indicate s.e.m. Source data

To test whether the activity of Sst-Chodl cells varies across sleep and wake states, as suggested by previous studies of FOS expression^14,15^, we expressed a GCaMP calcium indicator using either viral vectors or transgenic lines (see Methods) in *Sst*^*flp*^*;Nos1*^*creER*^ or *Sst*^*flp*^*;Chodl*^*cre*^ mice. We then used two-photon calcium imaging to measure the activity of labelled cells across arousal and network synchronization states, using pupillometry, locomotion, contralateral cortical local field potentials (LFPs; delta band, 1–4 Hz), electromyography (EMG) and facial movements (Fig. 2a,b and Supplementary Video 2). Because these state measurements were strongly correlated (Extended Data Fig. 4a), we combined them into a one-dimensional ‘arousal score’, corresponding to the first principal component of these state variables (Fig. 2b and Extended Data Fig. 4b). Behavioural state was classified into four categories: wakefulness, which was subdivided into movement and quiet wakefulness (quiet wake; QW); SWS; and rapid eye movement (REM) sleep (Fig. 2b; see Methods).

We found that a large proportion of labelled cells in superficial layers (L1–2/3) from both intersectional mouse lines exhibited intense activity during low-arousal, highly synchronous states such as SWS and QW, and were strongly suppressed during REM sleep and movement, when the neocortex is desynchronized (95 out of 111 cells; Fig. 2b,c and Extended Data Fig. 4c). A minor subset of labelled cells showed the opposite pattern of activation, being most active during periods of high movement (16 out of 111 cells; Extended Data Fig. 4c,d). This subset is consistent with our sequencing and immunohistochemistry analyses indicating the presence of a minor population of non-Sst-Chodl cells in both intersectional crosses, as well as with previously described^8,26^ small heterogenous populations of SST^+^nNOS^+^ neurons that do not express Chodl (Extended Data Fig. 4e).

We next examined the consistency of Sst-Chodl cell activity across cortical layers using microprism-based imaging (Extended Data Fig. 4f). Similar to superficial-layer neurons, Sst-Chodl cells recorded in deep layers were strongly active during SWS and QW and suppressed during movement and REM sleep (14 out of 16 cells; Extended Data Fig. 4g). Across all layers, Sst-Chodl cells that were active during low-arousal states exhibited strong positive correlations with low-arousal metrics, including delta-band spectral power and time elapsed since last locomotion (that is, stillness duration), and negative correlations with high-arousal measures such as EMG, facial motion, locomotion and pupil diameter (Fig. 2d). Activity among these cells was also highly correlated and dependent on the mouse’s arousal level, with stronger correlations observed during low-arousal states (Fig. 2d and Extended Data Fig. 4h). Consistent with this relationship, separating LFP epochs on the basis of periods of high versus low Sst-Chodl cell activity revealed that these neurons were most active when cortical networks were highly synchronized, as indicated by increased (high) delta power (Extended Data Fig. 4i–k).

To examine the temporal relationship between Sst-Chodl cell activity and cortical dynamics, we implanted flexible 32-channel probes beneath the imaging window, to enable two-photon imaging of Sst-Chodl cell activity with high temporal resolution alongside electrophysiological recordings (Fig. 2e–k; see Methods). Sst-Chodl cells exhibited state-dependent changes in activity that closely tracked fluctuations in LFP delta power (Fig. 2f and Extended Data Fig. 4l). Sst-Chodl cell activity closely co-varied with delta power, rather than anticipating future changes in cortical synchrony (Fig. 2g,h and Extended Data Fig. 4m). In addition, neither the mean activity of Sst-Chodl cells during SWS bouts nor other examined features were correlated with the duration of SWS bouts (Extended Data Fig. 4n,o). Together, these results suggest that the activity of Sst-Chodl cells reflects ongoing sleep-related cortical dynamics, rather than anticipating forthcoming state transitions.

We next examined Sst-Chodl cells on a shorter timescale during SWS, which alternates between phases characterized by a near-complete suppression of spiking during upward deflections of the cortical LFP (‘DOWN’ states) and phases of high spiking activity (‘UP’ states)^27^ (Fig. 2i). Aligning Sst-Chodl cell activity with DOWN state transitions revealed an increase in activity before the onset of the DOWN state, whereas other neurons, simultaneously recorded as multi-unit spiking activity from the same recording site, exhibited prominent rebound firing after DOWN-to-UP transitions (UP onset; Fig. 2j,k). Notably, Sst-Chodl cell activity was not correlated with either the duration or the amplitude of the subsequent DOWN state (Extended Data Fig. 4p–q), suggesting that these neurons are preferentially engaged near the termination of cortical UP states rather than during the quiescent DOWN phase itself.

**Extended Data Fig. 5:**
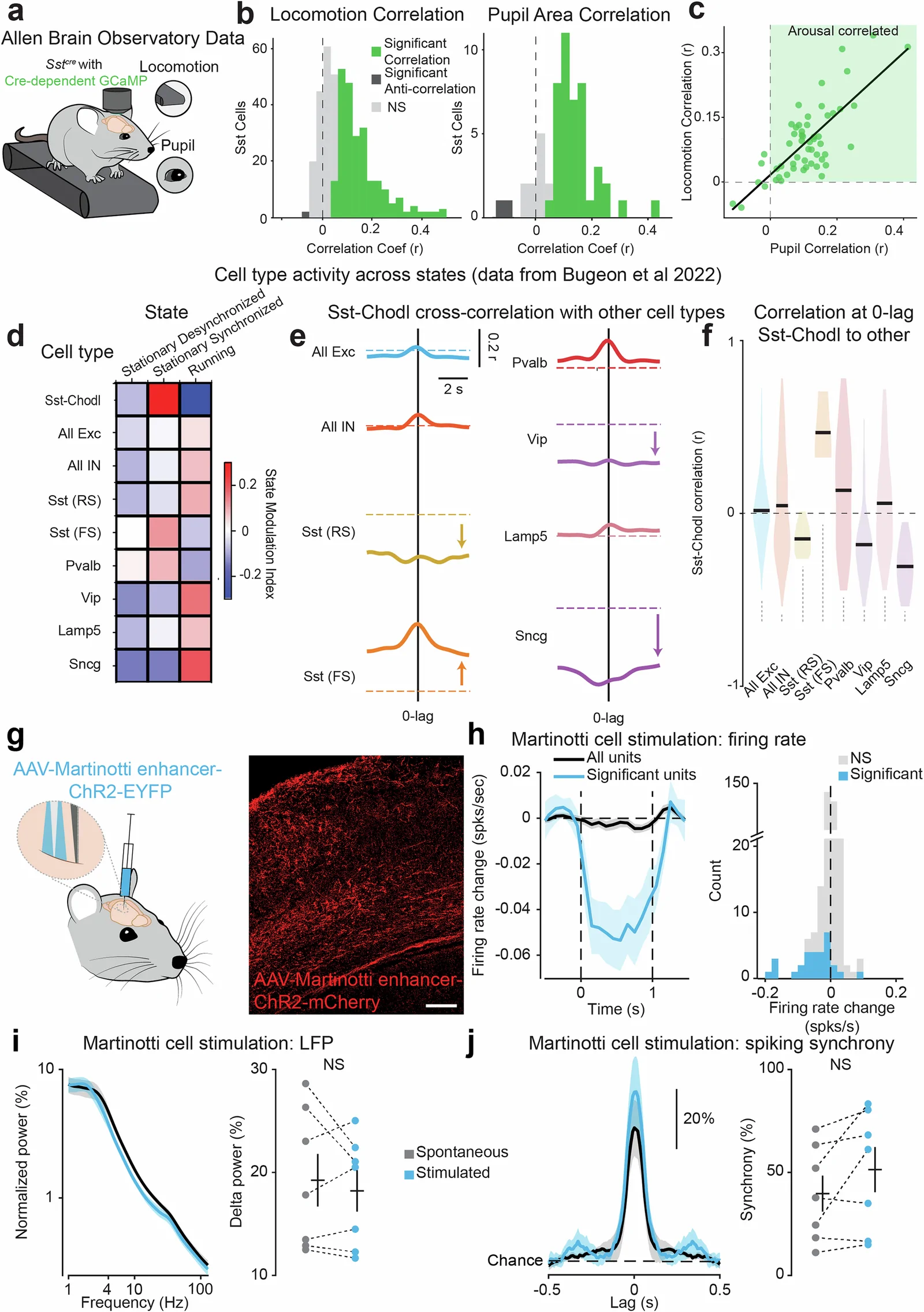
Sst-Chodl cells differ from other Sst and inhibitory subtypes in arousal modulation and synchrony. **a)** Schematic of Allen Brain Observatory^28^ recordings in which Sst cells are imaged with two-photon calcium imaging while pupil diameter and locomotion are monitored. **b)** Distribution of correlations between Sst cell activity and locomotion (left; n = 404 cells from 47 sessions) and pupil diameter (right; n = 55 cells from 9 sessions). Most Sst cells show positive correlations with arousal-linked measures. **c)** Scatter plot of pupil vs locomotion correlations for Sst cells (n = 55 cells from 9 sessions), illustrating that Sst cells in these datasets predominantly increase activity with heightened arousal. **d)** Correlations between activity and arousal state across major neuronal subtypes (dataset from ref. ^29^). Cell counts: 6684 Exc, 1954 IN, 414 Pvalb, 87 Sst (regular spiking; RS), 24 Sst (fast spiking; FS), 2 Sst-Chodl, 392 Vip, 543 Lamp5, 53 Sncg cells. This comparison shows that most inhibitory and excitatory classes are positively modulated by arousal, whereas Sst-Chodl cells exhibit the opposite pattern. **e)** Cross-correlation between Sst-Chodl cells and other neuronal subtypes, showing their relationships to arousal-modulated populations. **f)** Distribution of zero-lag correlations between Sst-Chodl cells and other cell types. Cell counts: 812 Exc, 368 IN, 56 Pvalb, 8 Sst (RS), 3 Sst (FS), 51 Vip, 77 Lamp5, 10 Sncg. Fast-spiking Sst cells include Sst-Tac1-Tacr3 and Sst-Tac1-Htr1d subtypes; regular-spiking Sst cells include all non-FS and non-Chodl Sst subtypes. Black lines denote means. These datasets show that Sst-Chodl cells are negatively correlated with arousal-activated inhibitory and excitatory populations and most positively correlated with fast-spiking Sst subtypes. **g)** Left: schematic of the experimental strategy using an AAV carrying a Martinotti-cell–specific enhancer driving ChR2-YFP expression in V1 of head-fixed mice. Right: example ChR2 expression. Scale, 100 µm. This approach selectively activates a major non-Chodl Sst subtype (Martinotti cells). **h)** Change in firing rate during Martinotti-cell stimulation (0 to 1). The firing rate of 30 out of 361 units was found to be significantly changed during optogenetic activation. **i)** Left: power spectra density during spontaneous and stimulation periods. Right: quantification of delta-band (1–4 Hz) LFP power, showing no significant change with stimulation (*P* = 0.460, paired *t*-test). This indicates that Martinotti-cell activation does not increase low-frequency power. **j)** Left: average normalized cross correlogram (CCG) for unit pairs during spontaneous and stimulation epochs. Right: quantification of pairwise synchrony, showing no significant change (*P* = 0.100, paired *t*-test). Thus, unlike Sst-Chodl cells, Martinotti cells do not induce increases in spiking synchrony. N = 7 sessions across 3 mice; 361 units and 28,016 pairs. NS, not significant. Data are means; shading and bars indicate s.e.m. The mouse cartoons in **a**,**g** are adapted with permission from ref. ^53^, Cell Press. Source data

Finally, to evaluate the specificity of patterns of Sst-Chodl cell activity, we compared their state modulation with that of the broader Sst population and other neuronal classes using two publicly available datasets. First, using the Allen Brain Observatory dataset^28^, we examined the correlations between Sst cell activity and arousal metrics, including locomotion and pupil area state (Extended Data Fig. 5a). In this dataset, the whole Sst population was correlated predominantly with increased arousal (Extended Data Fig. 5b,c). Next, using an independent dataset^29^, we compared the activity of Sst-Chodl cells to that of other simultaneously recorded neuronal types (Extended Data Fig. 5d–f). In contrast to arousal-activated interneuron subtypes, including regular-spiking Sst cells, Sst-Chodl cells showed negative correlations with arousal (Extended Data Fig. 5d–f). Among IN populations, Sst-Chodl cells exhibited their strongest positive correlation with fast-spiking Sst subtypes, particularly a numerically minor Tac1-expressing population (Extended Data Fig. 5e,f).

Together, our results suggest that, unlike other Sst cell populations, Sst-Chodl cells closely mirror changes in arousal states in terms of their activity, becoming most active during highly synchronized cortical states.

## Sst-Chodl cells drive neocortical synchrony

**Fig. 3:**
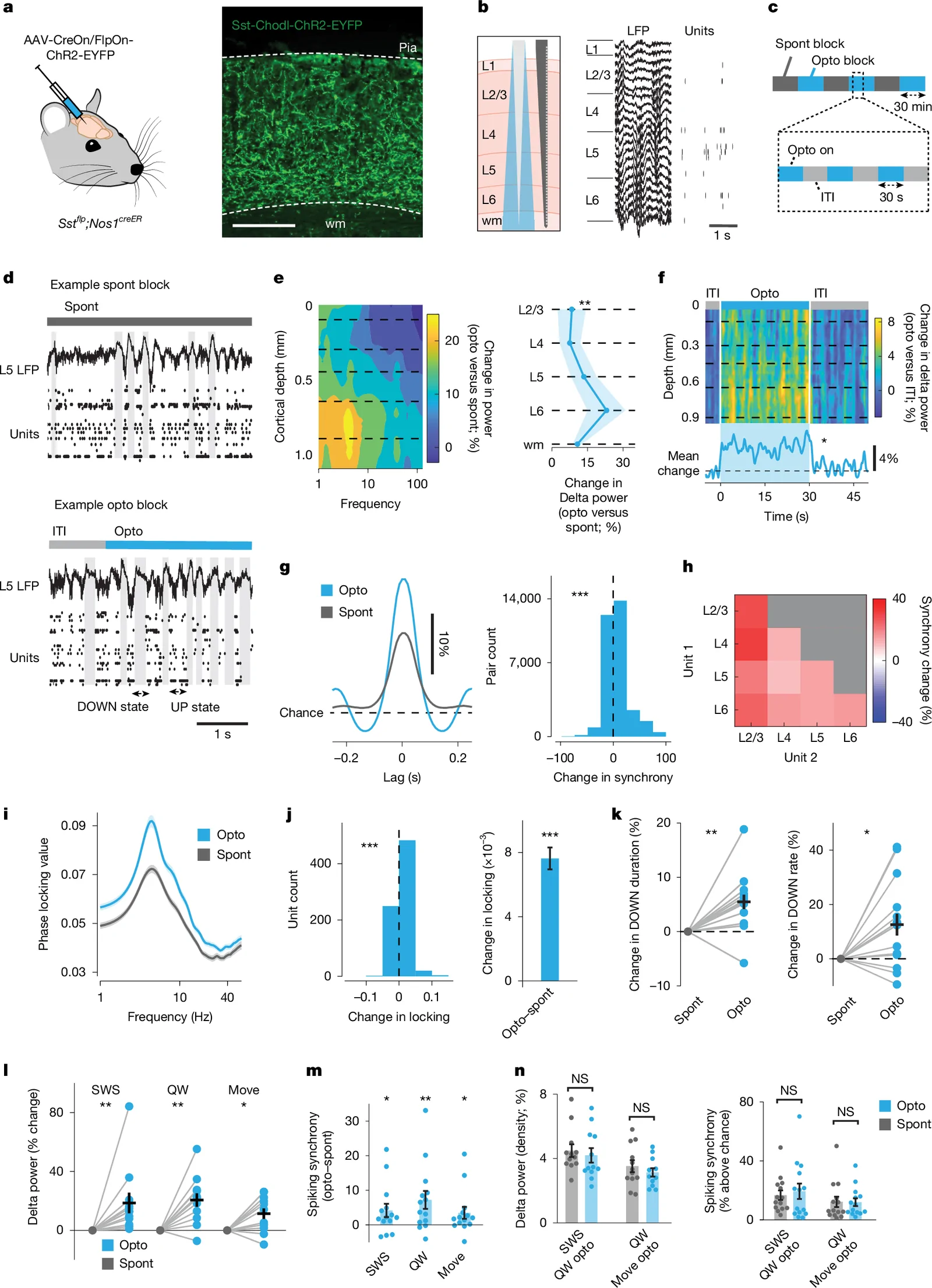
Optogenetic activation of Sst-Chodl cells induces robust neocortical network synchronization. **a**, Left, schematic of CreOn/FlpOn-ChR2-EYFP injections used to selectively express ChR2 in V1 Sst-Chodl cells. Right, example of ChR2 expression. Scale bar, 200 μm. **b**, Schematic of the 64-channel linear silicon probe with an attached tapered optical fibre, and example LFPs and isolated units aligned to cortical layer locations. **c**, Stimulation protocol consisting of spontaneous (spont; grey) and optogenetic stimulation (opto; blue) blocks. Opto blocks include repeated cycles of stimulation and ITIs. **d**, Example trials showing layer 5 LFPs and MUA during stimulation and ITIs. DOWN states are shaded. Stimulation increased the occurrence and duration of synchronized states. **e**, Left, mean LFP power change across depths (opto versus spont). Right, change in delta band (1–4 Hz) power during SWS (**P = 0.008, paired, two-sided *t*-test). This demonstrates that Sst-Chodl cell activation enhances low-frequency power throughout the cortical column. **f**, Average delta-band power change across layers, with mean change below (mean opto versus ITI; *P* = 0.004, paired two-sided *t*-test). Stimulation rapidly increases delta power relative to the onset of blue light. **g**, Normalized spike-train cross-correlograms (CCGs) during spontaneous and stimulated periods. Left, mean CCG relative to chance. Right, distribution of CCG peak changes for all neuron pairs (units: difference in chance-normalized coincident spikes, ***P < 10^−324^, paired two-sided *t*-test). Stimulation increases spiking synchrony across the population. **h**, Change in pairwise spiking synchrony across cortical layers, showing that synchrony increases broadly and is not layer specific. **i**, Spike phase coherence across the LFP frequency spectrum during spontaneous and stimulation blocks. Sst-Chodl cell activation enhances coherence in the delta band. **j**, Distribution (left) and mean (right) of unit-wise change in delta-band phase locking with stimulation (***P < 10^−324^, paired two-sided *t*-test). Spikes become more tightly locked to delta oscillations during stimulation. **k**, Increased DOWN-state duration and event rate during stimulation relative to spontaneous periods (**P = 0.002 and *P = 0.02, paired two-sided *t*-test). These changes indicate longer and more frequent synchronized OFF periods. **l**, Delta power change across SWS, QW and movement (move) (SWS, **P = 0.008; QW, **P = 0.001; move, *P = 0.027, paired two-sided *t*-test). Stimulation increases delta power across all states, even during movement, when delta power is typically low. **m**, Change in pairwise spiking synchrony across SWS, QW and movement (SWS, *P = 0.030; QW, **P = 0.004; move, *P = 0.035, linear mixed-effects model). Synchrony increases across all states with stimulation. **n**, Comparison of delta power (density) and spiking synchrony (% above chance level) during spontaneous and stimulated conditions for SWS and QW (repeated-measures ANOVA *P* < 10^−324^, with post-hoc two-sided Tukey’s HSD: delta: SWS–QW opto, *P* = 0.351 (NS); QW–move opto, *P* = 0.700 (NS); SWS–move opto, *P* = 0.003; SWS–QW, *P* = 0.003; QW opto–move opto, *P* = 0.034. Spiking synchrony: SWS–QW opto, *P* = 0.207 (NS); QW–move opto, *P* = 0.221 (NS); linear mixed-effects model). Stimulation during QW increases delta power and spiking synchrony to SWS-like levels. This shows that Sst-Chodl cell activation can drive synchrony to higher physiological levels. These experiments were performed in V1 of head-fixed mice. *n* = 14 sessions across 10 mice, *n* = 758 units and 32,710 pairs. ***P < 0.001, **P < 0.01, *P < 0.05; NS, not significant. All opto–spont comparisons were made during SWS. Data are mean ± s.e.m. (bars and shading). The mouse cartoon in **a** is adapted with permission from ref. ^53^, Elsevier. Source data

**Extended Data Fig. 6:**
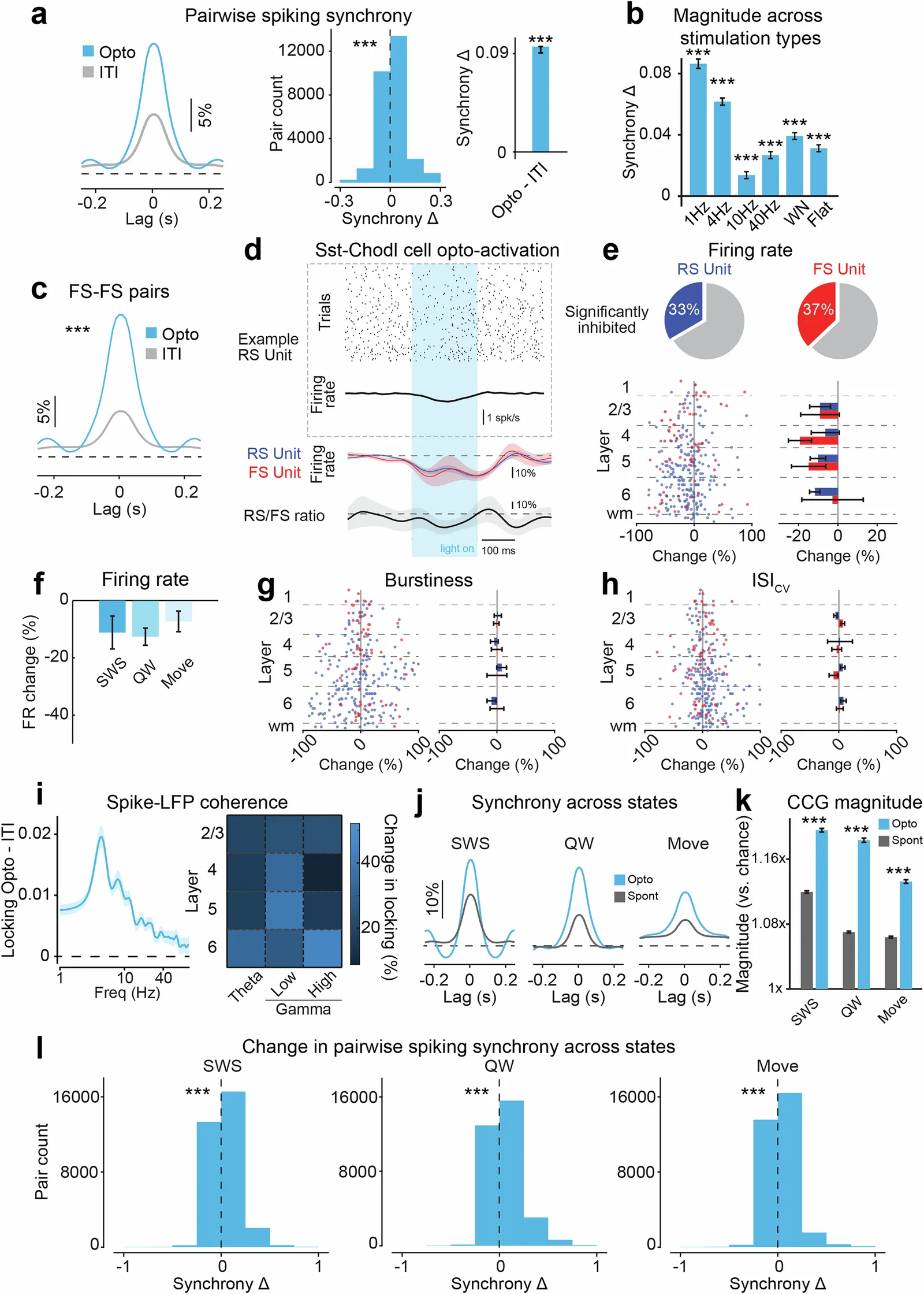
Sst-Chodl activation increases spiking synchrony across cell types and cortical layers. **a)** Normalized cross-correlograms (CCGs) comparing ITIs and stimulation periods. Distribution (left) and average (right) of CCG peak changes for all neuron pairs (*P* < 0.001, paired *t*-test). These analyses show that stimulation reliably increases pairwise spiking synchrony. **b)** Change in spiking synchrony across different stimulation protocols (*P* < 0.001, paired *t*-test; Opto vs. Spont), demonstrating that synchrony enhancement is robust across stimulation types. **c)** Spike-spike synchrony changes for fast-spiking (FS) cell pairs during Sst-Chodl cell stimulation ( *P*< 0.001, paired *t*-test; n = 19,918 pairs) revealing that FS interneurons also exhibit increased synchrony. **d)** Top: example regular-spiking (RS) unit response to Sst-Chodl cell stimulation. Bottom: average responses of RS and FS units to stimulation and RS:FS response ratio, indicating minor firing-rate modulation relative to synchrony changes. **e)** Top: proportion of significantly modulated RS and FS units (paired t-test, pre-stim vs post-stim). Bottom: firing-rate changes as a function of cortical depth (Opto vs. ITI). These results show small but systematic rate reductions in some units. **f)** Firing-rate changes across behavioural states (Opto vs ITI) confirming that synchrony increases cannot be explained by increased firing activity. **g)** Change in burstiness (autocorrelogram ratio 1.5-13/ 200-300 ms) across layers (Opto vs Spont). **h)** Change in coefficient of variation of interspike intervals across layers (Opto vs Spont). Together, these metrics indicate minimal changes in spike structure despite increased synchrony. **i)** Spike-LFP coherence across frequency bands and layers, showing significant increases during stimulation (Stimulation *P* < 0.001; no significant effects of Layer or Frequency band; linear mixed-effects model). **j**) Change in spiking synchrony across behavioural states (all cells, Opto vs Spont). **k)** Change in CCG peak synchrony across states (±50 ms window; ***P < 0.001 for all, paired *t*-test), indicating robust increases regardless of state. **l)** Distribution of change in spiking synchrony across behavioural states (Opto vs Spont). These experiments were performed in V1 of head-fixed mice. N = 14 sessions across 10 mice; n = 758 units and 32,710 pairs. ***P < 0.001. Data are means; bars indicate s.e.m. Source data

**Extended Data Fig. 7:**
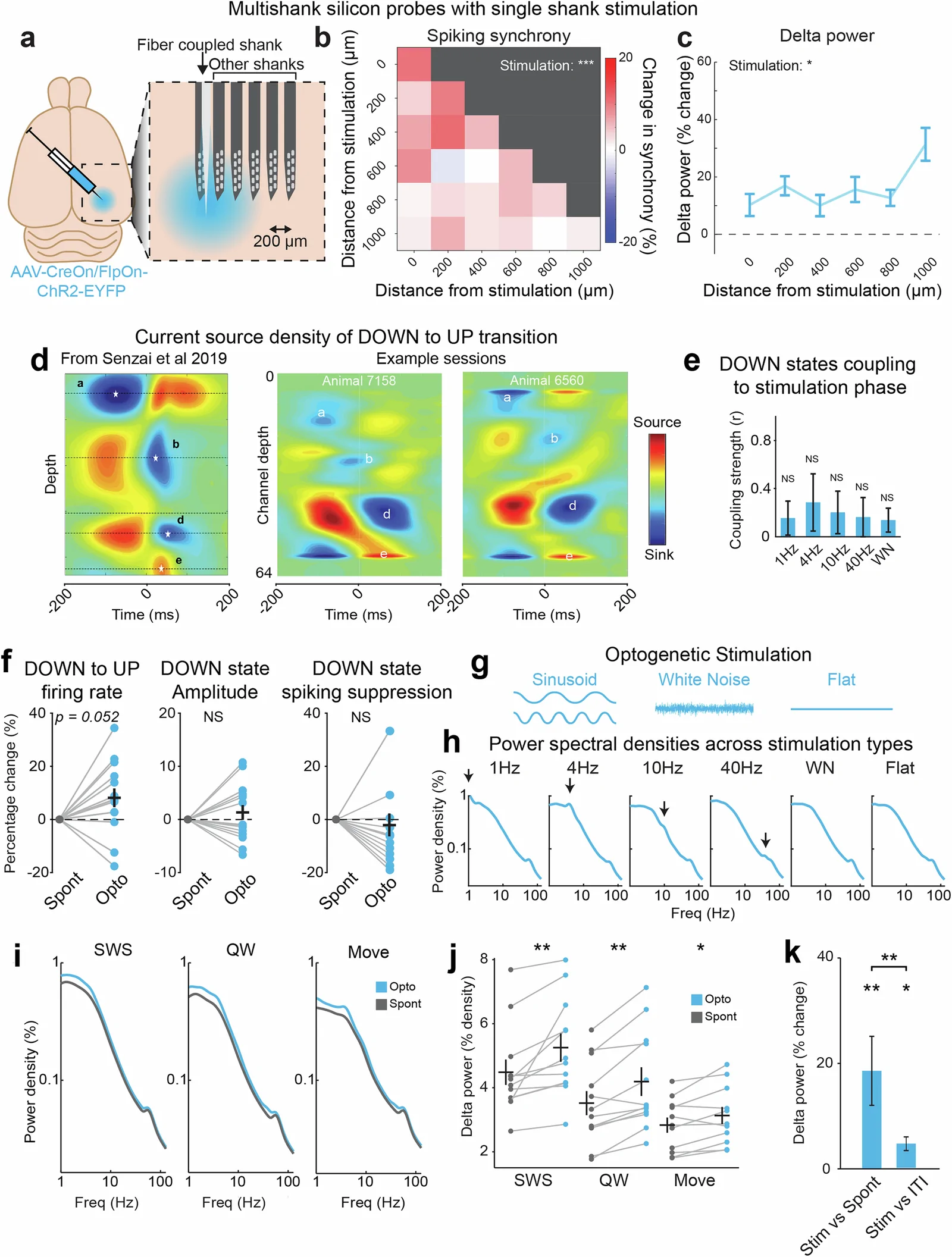
Sst-Chodl activation synchronizes activity across cortical sites and behavioural states. **a)** Schematic of the multishank 64-channel silicon probe inserted into visual cortex of head-fixed mice, with a tapered optical fibre attached to the most lateral (first) shank to enable single-shank optogenetic stimulation while recording units and LFPs across all shanks. **b)** Average change in cross-correlograms (CCGs) during optogenetic stimulation relative to spontaneous periods for unit pairs recorded on the stimulated shank (distance = 0) and on neighbouring shanks (spaced 200 µm apart). Stimulation significantly increased spiking synchrony across the array (linear regression model: stimulation effect ***P < 0.001, distance effect *P* = 0.133), indicating that the synchrony enhancement propagated well beyond the directly stimulated site. **c)** Change in delta-band (1–4 Hz) power across shanks during stimulation (linear mixed-effects model with post-hoc ANOVA: stimulation effect *P = 0.049, distance-stimulation interaction *P* = 0.270). Delta power increased across all shanks, showing that Sst-Chodl cell activation induces distributed low-frequency synchronization across visual cortex. N = 18 sessions from 8 mice. **d)** Left, average current source density (CSD) patterns during freely moving natural sleep from ref. ^70^. Right, example CSDs during head-fixed sleep in this study, showing sinks and sources labelled (a-e) corresponding to motifs described in^70^. These similarities indicate that DOWN-UP dynamics in the head-fixed preparation resemble natural sleep. **e)** Coupling strength of DOWN states to the phase of optogenetic stimulation (Rayleigh test for non-uniformity, NS, *P* > 0.05). DOWN states did not become phase-locked to the stimulation cycle, indicating that stimulation increased synchrony without imposing a fixed phase structure. **f)** Left: change in firing rate during DOWN to UP states; middle: change in DOWN-state LFP amplitude; right: suppression of spiking during DOWN states. None of these features differed significantly between stimulation and spontaneous periods (NS for all, Firing rate, *P* = 0.052; Amplitude, *P* = 0.496; Suppression, *P* = 0.361; paired *t*-tests), suggesting that Sst-Chodl cell activation does not strongly alter the intrinsic structure of DOWN-state events. **g)** Schematic of stimulation regimes used to probe frequency-dependent network responses. **h)** Power spectra for each stimulation protocol; arrows indicate stimulation frequencies. Optogenetic activation produces characteristic increases in low-frequency power. **i)** Change in LFP power spectra across states (SWS, QW, movement (Move) during stimulation of Sst-Chodl cells. **j)** Delta-band (1–4 Hz) power increases across all states during stimulation (SWS, **P = 0.008; QW, **P = 0.001; Move *P = 0.027; paired *t*-tests), demonstrating that Sst-Chodl cells elevate low-frequency power even when mice are awake or moving. **k)** Delta power change across different epochs when comparing stimulation, ITIs, and spontaneous periods (overall, **P = 0.008; Stim vs ITI, *P = 0.037; Stim vs Spont, **P = 0.005; paired *t*-tests). These analyses confirm that Sst-Chodl cell activation reliably increases delta power regardless of behavioural epoch. These experiments were performed in V1 of head-fixed mice. N = 14 sessions across 10 mice. **P < 0.01, *P < 0.05; NS, not significant. Data are means; shading and bars indicate s.e.m. Panel **d** is reproduced with permission from ref. ^70^, Elsevier, under a Creative Commons licence CC BY 4.0. Source data

To test whether the activity of Sst-Chodl cells causally promotes neocortical synchrony, we acutely inserted 64-channel linear silicon probes equipped with tapered optical fibres into the V1 of head-fixed mice virally expressing channelrhodopsin (ChR2) in Sst-Chodl cells (Fig. 3a,b). We quantified multiple complementary metrics of cortical synchrony derived from LFP and spiking activity to compare network states with and without optogenetic stimulation of Sst-Chodl cells (Fig. 3c–n and Extended Data Figs. 6 and 7).

We first examined changes in LFP power across cortical depth during stimulation relative to non-stimulation (spontaneous) periods. Optogenetic activation of Sst-Chodl cells led to a rapid and robust increase in delta band power, across all layers, with the largest effects observed in deeper layers (Fig. 3e,f). We next assessed spiking synchrony among well-isolated single units (putative single neurons) by computing spike train cross-correlations. Sst-Chodl cells activation increased the magnitude of spiking synchrony across all layers (Fig. 3g,h and Extended Data Fig. 6a), independently of stimulation frequencies (Extended Data Fig. 6b) or unit class (regular spiking versus fast spiking; Extended Data Fig. 6c). Although Sst-Chodl cell activation produced a slight reduction in the overall firing rate, spike-train structure and burst properties were mostly preserved (Extended Data Fig. 6d–h), indicating that enhanced synchrony did not result from increased firing but instead reflected changes in temporal coordination across neurons. Moreover, single-shank stimulation using a 6-shank (200-µm spacing), 64-channel silicon probe reliably increased delta power and spiking synchrony at the stimulated site. Notably, these effects propagated across all shanks, spanning around 1 mm of cortex (Extended Data Fig. 7a–c). This spatial spread indicates that activation of Sst-Chodl cells induces distributed synchronization across visual cortical networks.

Given the close relationship between LFP fluctuations and spiking activity, we next assessed spike–field coupling during stimulation by quantifying spike phase locking values. Under baseline conditions, spikes were preferentially locked to the delta phase of the LFP (Fig. 3i), and Sst-Chodl cell stimulation strongly enhanced this coupling (Fig. 3i,j and Extended Data Fig. 6i). As an extreme manifestation of phase locking, DOWN states became more frequent and longer-lasting during stimulation, with event rate and duration both significantly increased (Fig. 3k and Extended Data Fig. 7d–f).

We then asked whether Sst-Chodl cell activation modulates cortical synchrony across different states. Comparing stimulation epochs with matched spontaneous periods revealed significant increases in delta-band power during SWS, QW and movement (Fig. 3l and Extended Data Fig. 7i,j; REM was excluded owing to insufficient bout duration). Spiking synchrony was similarly enhanced across all three states (Fig. 3m and Extended Data Fig. 6j–l). Of note, synchrony increased even during movement, when Sst-Chodl cells are typically not active endogenously (Fig. 3l–n). When comparing the magnitude of stimulation-induced synchrony to physiological levels, we found that Sst-Chodl cell activation during QW increased delta power and spiking synchrony to levels comparable with those seen in spontaneous SWS, whereas stimulation during movement produced a delta power comparable with that observed in spontaneous QW (Fig. 3n and Extended Data Figs. 6j,k and 7i,j).

Together, these findings demonstrate that activation of Sst-Chodl cells robustly enhances neocortical synchrony, recapitulating key features of endogenous synchronized cortical states observed during SWS.

## Non-Chodl Sst cells do not drive synchrony

Next, we examined whether activation of a non-Chodl subtype of Sst interneurons is sufficient to induce cortical synchrony. To address this, we selectively expressed ChR2 in Martinotti cells, a major population of non-Chodl Sst cells, using an enhancer-driven viral strategy, and recorded cortical activity during optogenetic stimulation (Extended Data Fig. 5g). As expected, stimulation of Martinotti cells produced a reduction in the overall cortical firing rate (Extended Data Fig. 5h). However, in contrast to Sst-Chodl cell activation, this manipulation did not alter cortical synchrony, as measured by delta band LFP power or spiking synchrony (Extended Data Fig. 5i,j). These findings indicate that the capacity to promote neocortical synchrony is not shared across all Sst INs but is instead a distinguishing feature of Sst-Chodl cells.

## Sst-Chodl cells regulate distant cortical areas

**Fig. 4:**
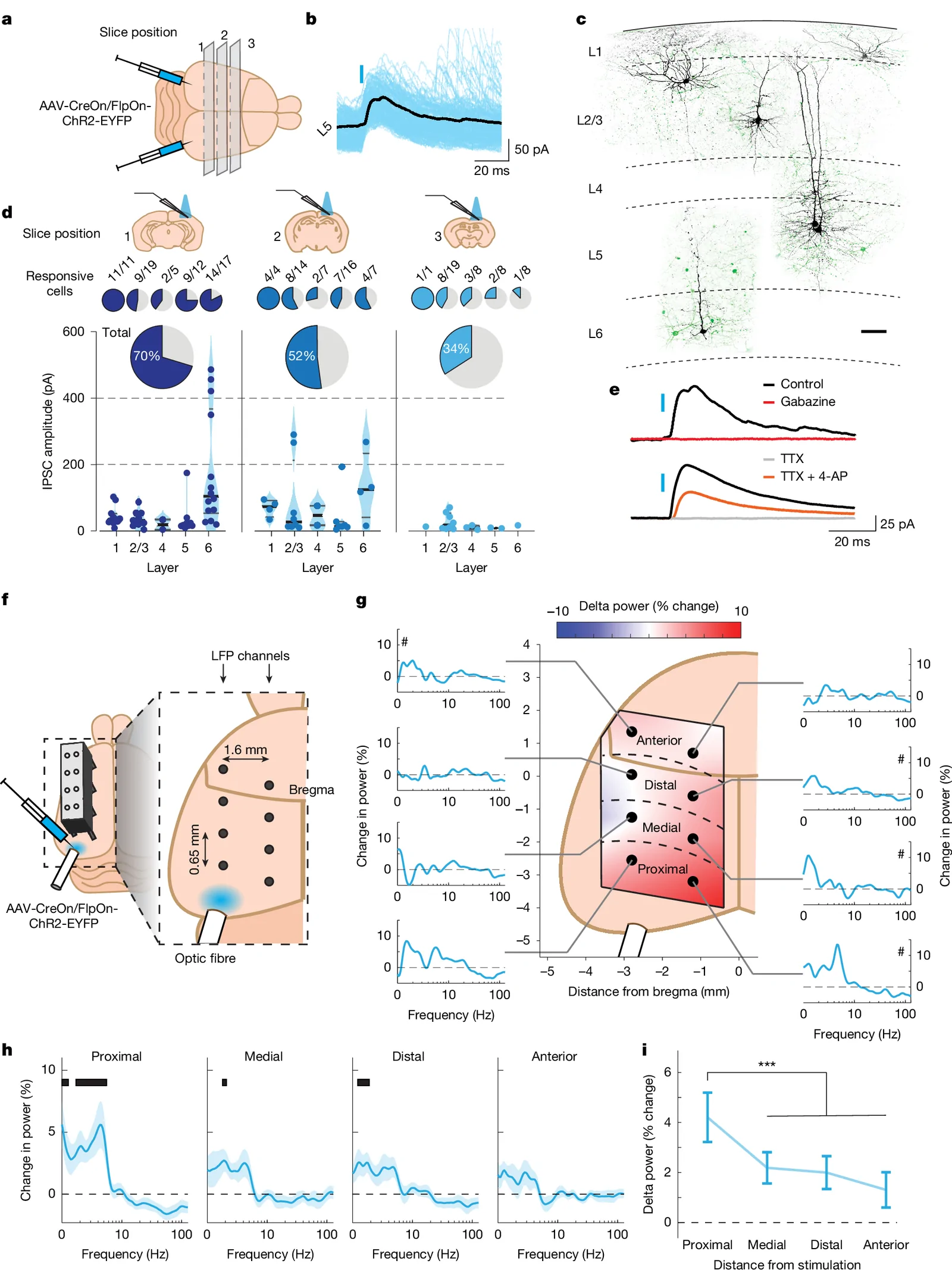
Sst-Chodl cells exert long-range inhibition and promote network synchronization across the neocortex. **a**, Illustration of the patch-clamp approach for identifying long-range postsynaptic targets of Sst-Chodl cells after expressing ChR2 in V1. **b**, Example oIPSCs (blue) and averaged response (151 traces; black) from a layer 5 pyramidal neuron aligned to the light stimulus (blue tick). These recordings confirm functional inhibitory synapses from Sst-Chodl axons. **c**, Schematic of cortical layers 1–6 near the injection site (V1) with representative anatomical fills of postsynaptic neurons (black) and ChR2-expressing Sst-Chodl fibres (green), highlighting the diversity of postsynaptic cell types encountered. Scale bar, 50 μm. **d**, Summary of responsive cells (blue) and oIPSC amplitudes across layers, grouped by anteroposterior distance from the injection site (anteroposterior distance from bregma, (1) −3.2 to −2.2; (2) −2.2 to −1.5; (3) −1.5 to −0.8; injection site is −3.2 mm). Horizontal lines indicate median and interquartile range. Pie charts indicate the percentage of neurons responding to Sst-Chodl cell stimulation. Although responsiveness declines with distance, nearly one-third of cells recorded about 2 mm from the injection site still showed oIPSCs, with layer 1 exhibiting the highest proportion of responsive neurons. Layer 6 responses showed the largest amplitudes. *n* = 16 mice (*Sst*^*flp*^*;Nos1*^*creER*^), *n* = 156 cells (44, 48 and 64 cells per distance bin). **e**, Top, representative oIPSC from a layer 5 cell under control conditions (black) and after application of gabazine (50 µM), confirming GABA_A_ receptor mediation (red).Bottom, tetrodotoxin (TTX; 0.5 µM) abolishes the oIPSC (grey), and addition of 4-aminopyridine (4-AP; 100 µM) restores it (orange), showing that these responses are monosynaptic. **f**, Illustration of the multi-site LFP recording strategy used to measure long-range network effects, with electrodes positioned around 2–6 mm from the V1 stimulation site. These experiments were performed on head-fixed mice. **g**, Example session showing stimulation-evoked changes in power spectra at each recording position (proximal, 2.1 mm; medial, 3.2–3.5 mm; distal, 4.4–4.8 mm; anterior, 5.6–6.1 mm from the injection site (all distances are approximate)). # denotes the channel exhibiting the largest delta band increase at each distance. **h**, Average power spectra from channels with the greatest delta increases at each distance. Black lines indicate frequency ranges with significant stimulation-induced changes (linear mixed-effects model with two-sided Bonferroni post-hoc tests, *P* < 0.05; see Source data for exact *P* values). **i**, Average change in delta power (1–4 Hz) as a function of distance from the stimulation site (repeated-measures ANOVA on linear mixed-effects model: distance effect *P* = 0.005; two-sided Bonferroni post-hoc tests: proximal versus medial, ***P = 7.9 × 10^−5^; proximal versus distal, ***P = 2.5 × 10^−5^; proximal versus anterior, ***P = 5.8 × 10^−5^; medial versus distal versus anterior, *P* = 1). Sst-Chodl cell activation induces widespread low frequency that diminishes with increasing distance from V1. *n* = 14 sessions from 7 mice. Data are mean ± s.e.m. (bars and shading). Source data

**Extended Data Fig. 8:**
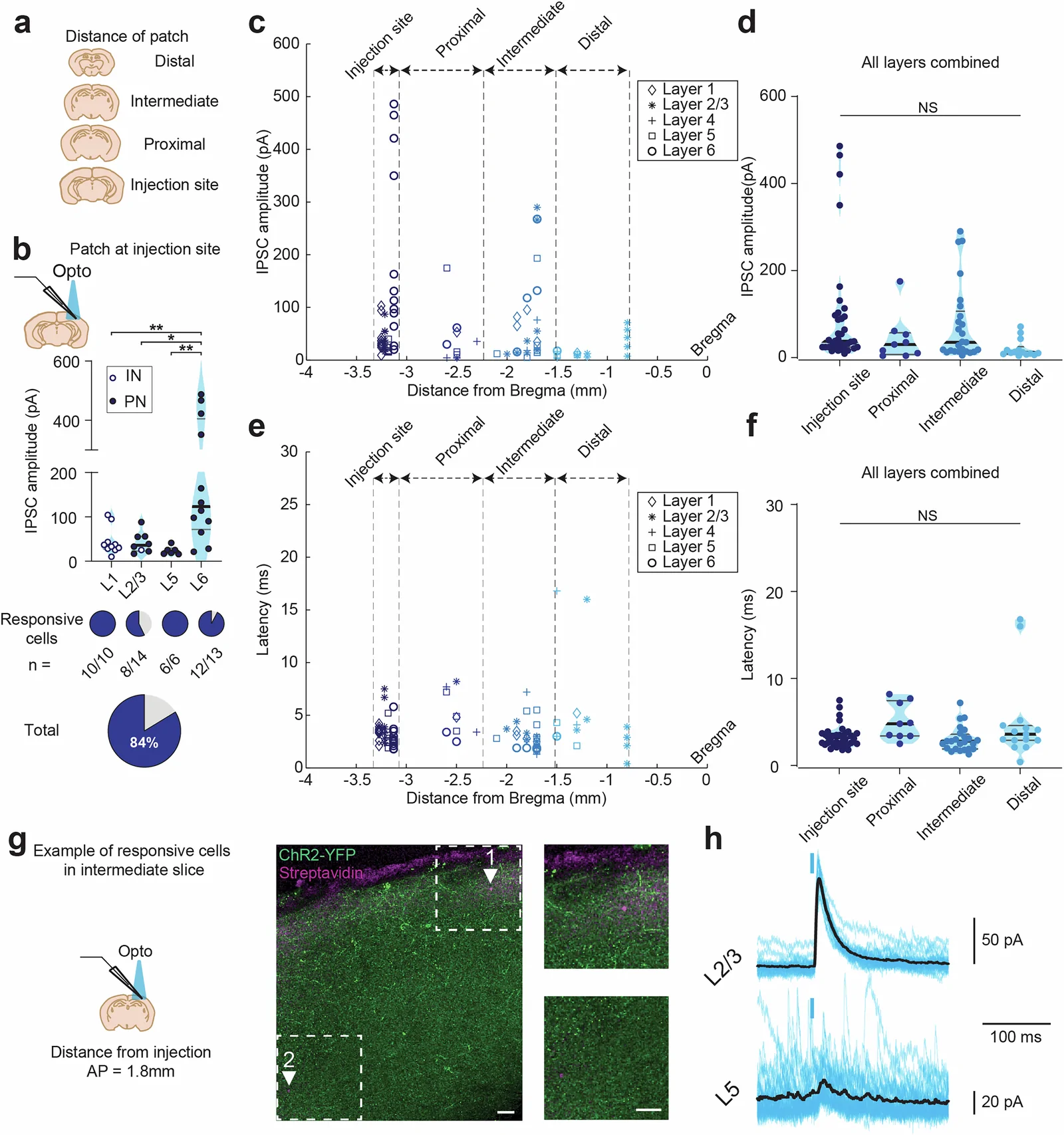
Sst-Chodl cells provide widespread monosynaptic inhibition across neocortical layers and distances. **a)** Schematic of acute slices recorded at different distances from the V1 injection site (injection site: −3.2 mm from bregma; Proximal: −3.19 to −2.21 mm; Intermediate: −2.2 to −1.51 mm; Distal: −1.5 to 0.79 mm). This strategy allowed assessment of long-range inhibitory connectivity of Sst-Chodl cells. **b)** oIPSC recorded at the injection site. Top: oIPSC amplitude across layers for putative interneurons (IN; blue/white circles) and putative pyramidal neurons (PN; black/blue circles). Bottom: pie charts showing the proportion of neurons in each layer responding to Sst-Chodl activation (One-way ANOVA interaction *P* = 0.001; L1 vs L6, **P = 0.005; L2/3 vs L6 *P = 0.013; L5 vs L6, **P = 0.009; other comparisons *P* > 0.9; n = 43 cells from N = 10 mice). Layer 1 exhibited the highest proportion of responsive cells. Layer 6 showed the largest amplitudes. These results confirm dense local inhibition across multiple layers. **c)** oIPSC amplitudes across all distances and layers. Dashed lines mark boundaries between distance categories (ANOVA interaction on linear mixed-effects model *P* = 0.794; Layer effect *P* < 0.001). oIPSC amplitudes varied by layer but remained detectable even at distal recording sites, consistent with widespread long-range inhibition. **d)** Mean oIPSC amplitude across layers and distances (Distance effect *P* = 0.795). **e**,**f)** Same analyses as in **b**,**d** but for oIPSC latency. Latency did not differ significantly across layers or distances (ANOVA interaction *P* = 0.906; Layer effect *P* = 0.321; Distance effect *P* = 0.604), indicating monosynaptic connectivity throughout the sampled neocortical territory. Horizontal lines indicate median (black) and interquartile range (grey). N = 16 mice, n = 156 cells (Injection site, 43 cells; Proximal, 21 cells; Intermediate, 48 cells; Distal, 44 cells). **g)** Example of a distant slice (AP −1.8 mm) showing two responsive cells filled with streptavidin (magenta) in L2/3 and L5 (arrows), together with ChR2-expressing Sst-Chodl fibres (green). These data demonstrate long-range innervation at distant cortical sites, including fibres within superficial layers, consistent with the anatomical projection pattern. Dashed squares are shown enlarged on the right (top, 1; bottom, 2). Scale, 50 µm. **h)** Individual oIPSCs (blue) with averaged responses (25-30 traces; black) aligned to the light stimulation (blue rectangle) from the cell filled in (**g**). Strong oIPSCs from distal locations further confirm long-range monosynaptic inhibition. N = 16 mice; n = 156 cells total (Injection site: 43; Proximal: 21; Intermediate: 48; Distal: 44). ***P < 0.001, **P < 0.01, *P < 0.05, NS = not significant. Source data

We next asked whether Sst-Chodl cells influence neuronal activity at more-distant neocortical sites, by determining the spatial extent of their synaptic targets. We expressed ChR2 in Sst-Chodl cells in V1 and performed whole-cell patch-clamp recordings from putative postsynaptic neurons in acute neocortical slices up to about 2.4 mm from the injection site (Fig. 4a,b and Extended Data Fig. 8). Optogenetically evoked inhibitory postsynaptic currents (oIPSCs) were observed in both pyramidal neurons and interneurons, with the highest response rates in V1 (the injection site), particularly in layers 1, 5 and 6 (Fig. 4c,d and Extended Data Fig. 8b–h). Although the proportion of responsive cells decreased with increasing distance (Chi-squared including layer 1 cells, *P* < 0.001; Chi-squared excluding layer 1 cells, *P* = 0.009), oIPSCs remained detectable in nearly one-third of neurons recorded around 2 mm from the injection site (Fig. 4d). Consistent with anatomical tracing (Fig. 1h and Extended Data Fig. 2c), layer 1 exhibited the highest proportion of responsive cells across all recording sites (Fig. 4d and Extended Data Fig. 8b), whereas layer 6 exhibited the largest response amplitudes (Fig. 4d and Extended Data Fig. 8b–c). Finally, in a subset of experiments, we confirmed that these oIPSCs were GABAergic and monosynaptic (Fig. 4e and Extended Data Fig. 8e,f). Together, these results align with our anatomical data and show that Sst-Chodl cells provide direct, long-range inhibition across neocortical layers.

Given the broad ipsilateral intracortical projections of Sst-Chodl cells, we next asked how far the synchronization induced by their activation propagates across the neocortex. To address this, we expressed ChR2 in Sst-Chodl cells in V1 and recorded LFPs from eight ipsilateral neocortical locations positioned well beyond the spatial extent sampled in our multishank recordings (Extended Data Fig. 7a–c), spanning distances of approximately 2–6 mm from the stimulation site (Fig. 4f). Compared with inter-trial intervals (ITIs), optogenetic activation of Sst-Chodl cells increased low-frequency LFP power across most of the recording sites, extending from the visual cortex to frontal motor areas (Fig. 4g–i). This effect was strongest at sites closest to the stimulation region, and declined progressively with increasing distance (Fig. 4i). Although we cannot exclude contributions from polysynaptic corticocortical propagation, these results suggest that Sst-Chodl cell activation can drive neocortical synchronization over millimetre-scale distances, with a graded decay consistent with long-range intracortical influence.

## Activation of Sst-Chodl cells promotes sleep

**Fig. 5:**
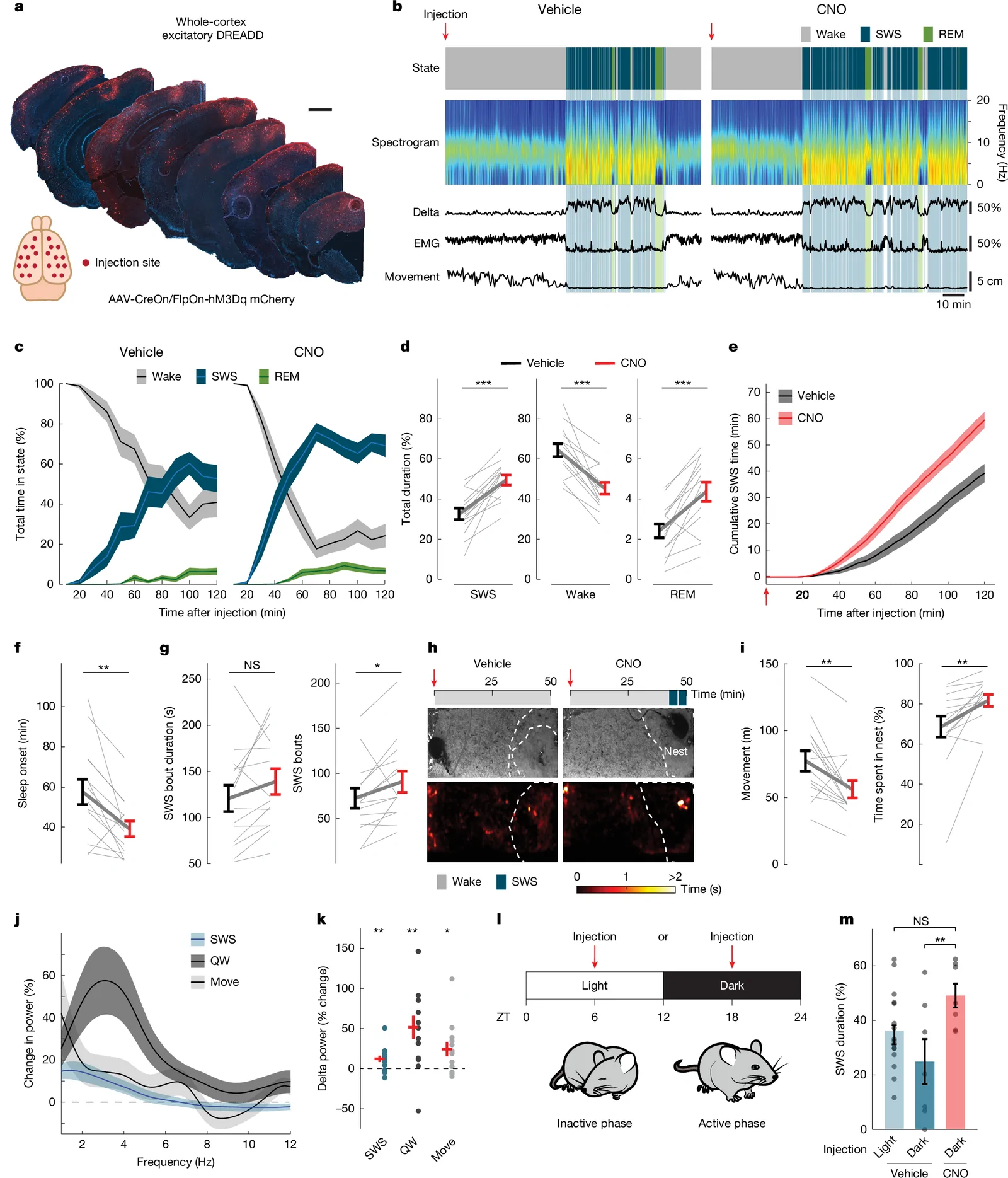
Chemogenetic activation of neocortical Sst-Chodl cells promotes sleep. **a**, Strategy for global activation of Sst-Chodl cells: an INTRSECT AAV-EF1a-fDIO-mCherry vector was injected at 22 neocortical sites (11 per hemisphere). Right, control brain showing broad expression of the CreOn/FlpOn-mCherry reporter. Scale bar, 1 mm. **b**, Example of freely moving recordings during the light (inactive) phase for one mouse after systemic injection of vehicle (left) or CNO (0.5 mg kg^−1^; right), showing sleep and wake states and associated physiological measures. CNO increased the time spent in sleep states. **c**, Percentage of time spent in wake, SWS and REM after injection of vehicle or CNO. **d**, Total time in each state across the 2 h post-injection window (SWS, ***P < 0.001; wake, ***P < 0.001; REM, ***P < 0.001, two-sided paired *t*-test). CNO increased both SWS and REM while reducing wake. **e**, Cumulative SWS time across sessions. **f**, Latency to sleep onset (**P = 0.003, two-sided paired *t*-test). **g**, SWS bout duration (*P* = 0.110 (NS), two-sided paired *t*-test) and number of SWS bouts (*P = 0.036, two-sided paired *t*-test). These data show that Sst-Chodl cell activation promotes sleep mainly by increasing bout number and reducing latency, rather than by lengthening individual bouts. **h**, Example mouse tracking from the first 50 min after injections. Top, sleep–wake scoring. Middle, mouse position and nest location (white dashed lines). Bottom: heat map of spatial occupancy. CNO-treated mice spent more time resting in the nest. **i**, Total distance travelled (**P = 0.005, two-sided paired *t*-test) and time in nest (**P = 0.003, two-sided paired *t*-test) over 2 h after injection. Reduced locomotion and increased nest occupancy mirror the behavioural shift toward sleep. **j**, LFP power changes recorded in V1 during SWS, QW and movement (Move). **k**, Delta-band (1–4 Hz) power changes across states (SWS, **P  0.008; QW, **P = 0.004; Move, *P = 0.018; two-sided paired *t*-tests). Chemogenetic activation increases low frequency across arousal states, consistent with the effects of optogenetic stimulation. **l**, Illustration of the experimental timing when mice were treated during the light (inactive) phase (zeitgeber time (ZT) 3–7) versus the dark (active) phase (ZT18). **m**, Comparison of SWS duration across light (inactive) and dark (active) conditions and for vehicle versus CNO. All data included (12 *Sst*^*flp*^*;Nos1*^*creER*^ mice, 4 *Sst*^*flp*^*;Chodl*^*cre*^ mice), light vehicle, *n* = 16, dark vehicle, *n* = 7, light CNO *n* = 7 mice; ANOVA on mixed-effects model, group effect *P* = 0.004; two-sided Bonferroni post-hoc tests: light vehicle versus dark vehicle, *P* = 0.214 (NS); light vehicle versus dark CNO, *P* = 0.100 (NS); dark vehicle versus dark CNO, **P = 0.003). CNO increased SWS in both phases, driving sleep even during the normally wake-dominant dark (active) period. These findings show that global activation of neocortical Sst-Chodl cells enhances cortical synchrony and promotes sleep across behavioural contexts and circadian phases. In **c**–**k**, *n* = 14 mice (11 *Sst*^*flp*^*;Nos1*^*creER*^ and 3 *Sst*^*flp*^*;Chodl*^*cre*^). ***P < 0.001, **P < 0.01, *P < 0.05; NS, not significant. Injections were performed during the light phase in **b**–**k**. Data are mean ± s.e.m. (bars and shading). The mouse cartoons in **l** are adapted with permission from ref. ^53^, Elsevier. Source data

**Extended Data Fig. 9:**
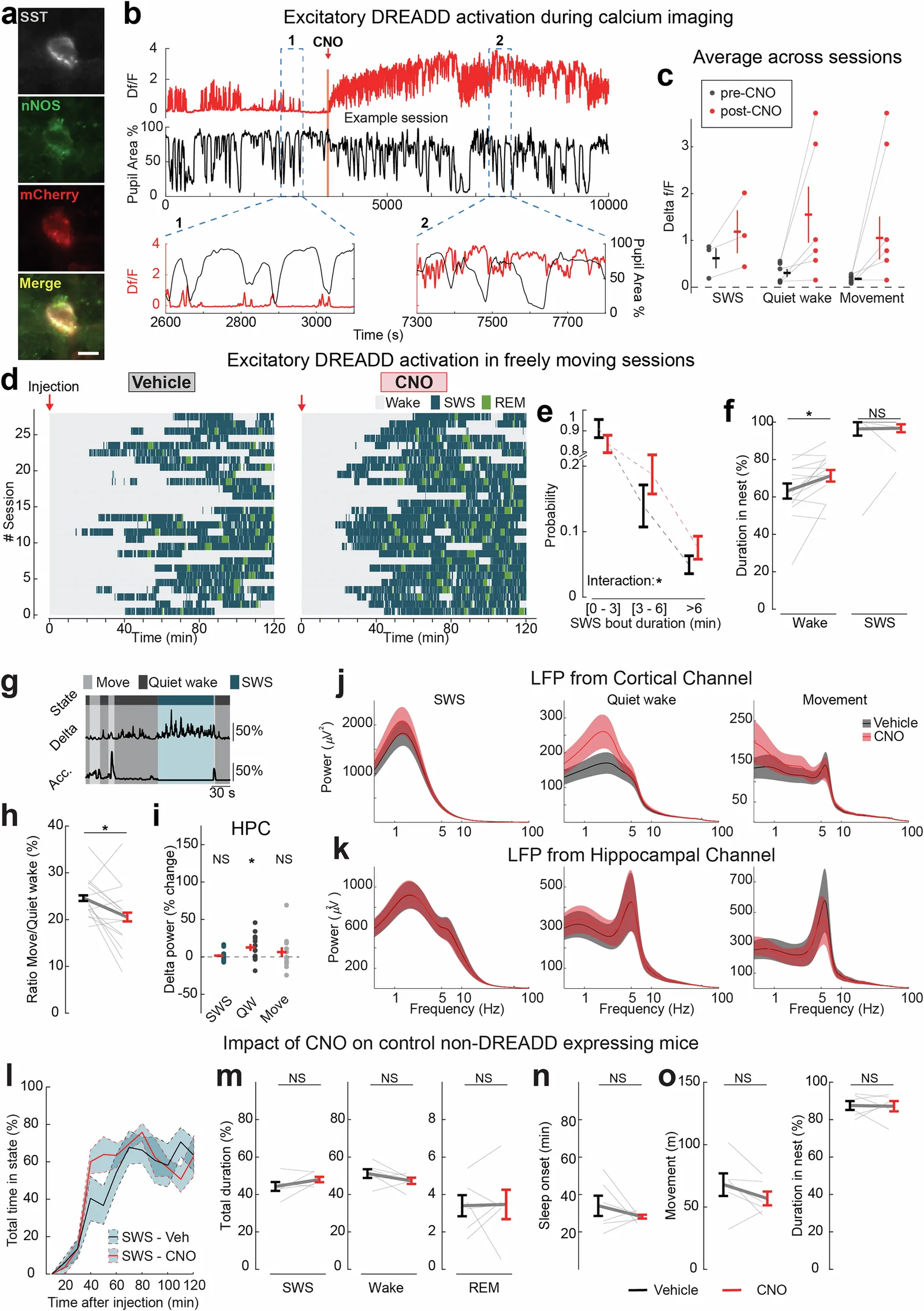
Neocortex-wide Sst-Chodl activation promotes sleep and increases cortical delta power. **a)** Histological example of a neocortical Sst-Chodl cell expressing the CreOn/FlpOn excitatory DREADD (hM3Dq–mCherry). Scale bar, 20 µm. **b)** Example two-photon recording showing increased calcium activity (red) in a DREADD-expressing Sst-Chodl cell following systemic CNO (0.5 mg kg^−1^), along with simultaneous pupil measurements (black). CNO increased activity regardless of arousal state. **c)** Mean calcium activity across behavioural states before (pre-CNO) and 15-60 min after CNO injection (post-CNO) (n = 6 cells from 4 mice). SWS was not present in sessions from the 3 recorded cells. CNO elevated Sst-Chodl cell activity broadly across states. **d)** Hypnogram from all sessions following Vehicle vs CNO injections, illustrating increased SWS and REM following Sst-Chodl cell activation. **e)** Distribution of SWS bout durations following pan-neocortical activation of Sst-Chodl cells (two-way ANOVA interaction *P = 0.040; 0–3 min, *P* = 0.091; 3–6 min, *P* = 0.118; >6 min, *P* = 0.373). Activation shifted the distribution mainly toward more bouts. **f)** Percentage of time spent in nest during Wake (*P = 0.013) and SWS (*P* = 0.864), showing that nest occupancy increases during CNO treatment (red). **g)** Example of delta amplitude and accelerometer signal across movement, QW and SWS. **h)** Ratio of movement to QW time following CNO injection (*P* = 0.028, paired *t*-test), indicating reduced wakeful activity. **i)** Delta-band (1–4 Hz) power change in the hippocampus (HPC) during SWS (NS, *P* = 0.376), QW (*P = 0.016), and movement (Move, *P* = 0.341). **j)** Power spectral densities (PSDs) during SWS (left), QW (centre), and movement (right) recorded from neocortical LFP channels after Vehicle or CNO injection. Chemogenetic activation of Sst-Chodl cells increased low-frequency power across behavioural states. **k**) Same analysis as in (**j**), but for HPC channels. Delta enhancements were weaker in HPC than in neocortex, indicating preferential cortical engagement by Sst-Chodl activation. **l)** Percentage of time spent in SWS after Vehicle or CNO injection in control mice that do not express DREADD. **m)** Total time in each state during the 2-hour window after injection for control mice (SWS, *P* = 0.146; Wake, *P* = 0.188; REM, *P* = 0.931; paired *t*-tests). CNO alone did not alter sleep architecture in controls. **n)** Latency to sleep onset in control mice (*P* = 0.344), showing no effect of CNO in the absence of DREADD. **o)** Total distance travelled (*P* = 0.145) and time spent in nest (*P* = 0.909) over 2 h in control mice. No behavioural changes occurred with CNO alone in control mice. No significant effects were observed, confirming that the sleep-promoting and synchronizing effects require activation of Sst-Chodl cells. NS = not significant; Two-sided paired *t*-test used for **f**,**h**,**i**,**m**–**o**. N = 14 mice for analyses in **d**–**k**; N = 6 mice for analyses in **l**–**o**. All injections were performed during the light (inactive) phase. *P < 0.05; NS = not significant. Data are means; shading and bars indicate s.e.m. Source data

**Extended Data Fig. 10:**
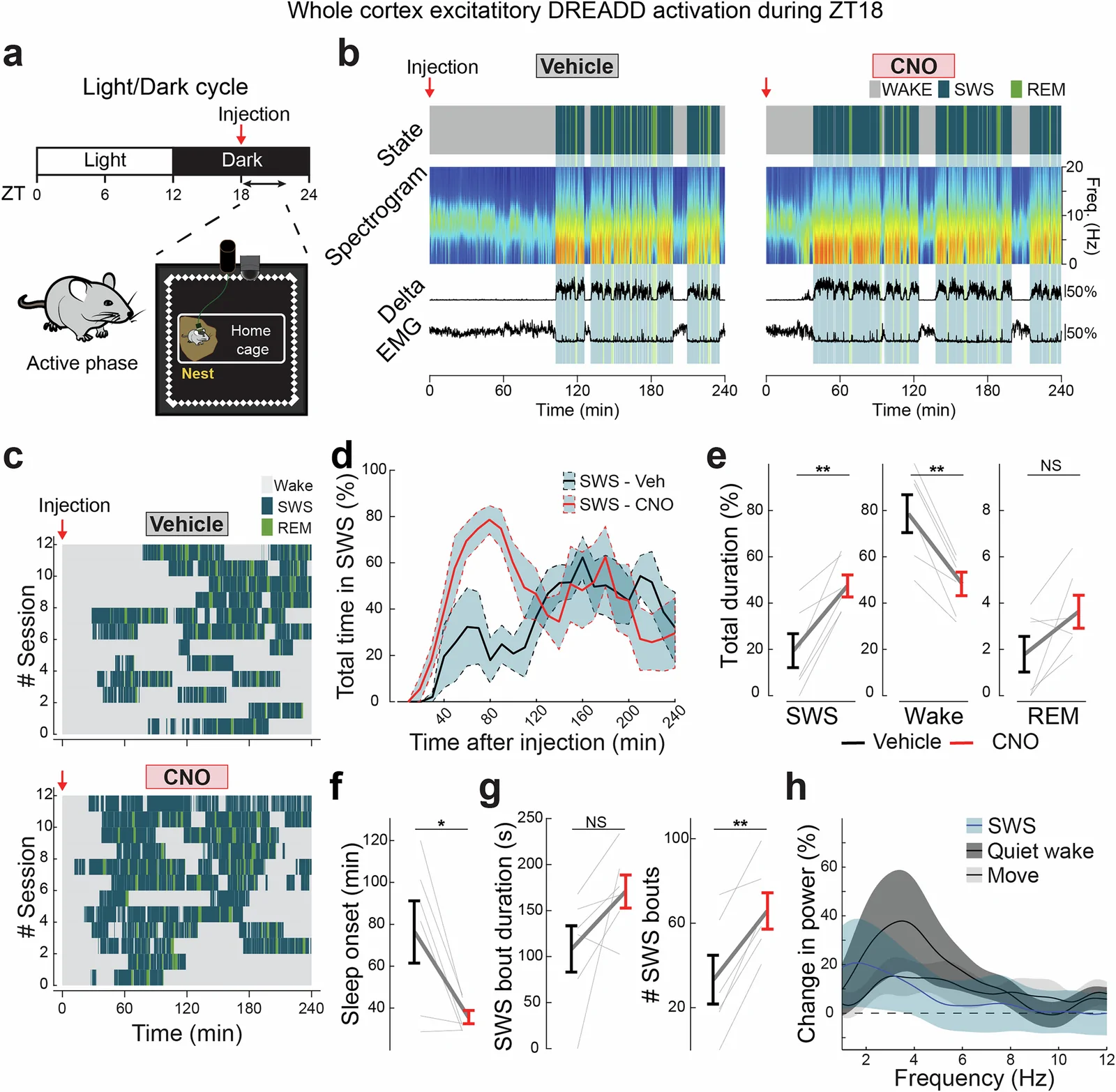
Sst-Chodl activation promotes sleep during the active phase. **a)** Experimental design for sleep monitoring during the dark (active) phase, with systemic injections performed at zeitgeber time 18 (ZT18), when mice are normally awake. **b)** Example recordings from one mouse during the dark (active) phase after Vehicle (left) or CNO (0.5 mg kg^−1^; right), showing sleep-wake scoring and associated physiological measurements. **c)** Hypnogram for all freely moving dark (active) phase sessions following Vehicle and CNO injections, illustrating a shift toward increased sleep during Sst-Chodl cell activation. **d)** Percentage of time spent in SWS after Vehicle vs CNO. **e)** Total time in each brain state during the 2-hour window after injection (SWS, **P = 0.003; Wake, **P = 0.002; REM, *P* = 0.077). CNO significantly increases SWS and reduces Wake, with a trend toward increased REM. **f)** Latency to sleep onset (*P = 0.025), showing faster transition into sleep when Sst-Chodl cells are activated. **g)** SWS bout duration (*P* = 0.093) and number of SWS bouts (**P = 0.002), showing that Sst-Chodl cell activation promotes sleep primarily by increasing bout number and reducing latency, rather than lengthening individual bouts. **h)** LFP power changes recorded in neocortical channels during SWS, QW and movement (Move) following CNO. Activation of Sst-Chodl cells increased low-frequency power across states. Together, these results are consistent with effects observed during the light (inactive) phase. **P < 0.01; *P < 0.05; NS = not significant. Paired *t*-test for all comparisons. N = 6 mice. Data are means; shading and bars indicate s.e.m. The mouse cartoon in **a** is adapted with permission from ref. ^53^, Cell Press. Source data

Previous work has suggested that during periods of intense SWS, putative Sst-Chodl cells are active throughout the neocortex^15^. Therefore, we hypothesized that widespread neocortical activation of these cells might not only enhance cortical synchrony (Figs. 3 and 4 and Extended Data Figs. 6 and 7), but also promote behavioural features of sleep. To test this, we used an INTRSECT-based chemogenetic approach to express an excitatory DREADD (designer receptors exclusively activated by designer drugs) in Sst-Chodl cells throughout the neocortex using 22 injected sites (11 per hemisphere) (Fig. 5a and Extended Data Figs. 9 and 10). Using two-photon imaging, we confirmed that systemic administration of a low dose of the DREADD agonist clozapine-*N*-oxide (CNO; 0.5 mg kg^−1^) increased the activity of Sst-Chodl cells, mostly independently of the arousal state of the mice (Extended Data Fig. 9b,c and Supplementary Videos 2 and 3). To test the effect of pan-neocortical Sst-Chodl cell activation on sleep behaviour, we performed counterbalanced systemic injections of vehicle or CNO and monitored LFP activity and movement in freely behaving mice in their home cage for 2 h during the light (inactive) phase of their circadian cycle (Extended Data Fig. 9d). Chemogenetic activation of Sst-Chodl cells increased the duration of both SWS and REM sleep, reduced sleep latency and increased the number of SWS bouts without affecting bout duration (Fig. 5b–g and Extended Data Fig. 9e). These changes were accompanied by decreased locomotion and increased nest occupancy (Fig. 5h,i and Extended Data Fig. 9f), consistent with a reduced proportion of time spent in movement relative to quiet wakefulness (Extended Data Fig. 9g,h) and with the observed increase in sleep in CNO-treated mice (Fig. 5b–g).

Similarly to optogenetic manipulation (Fig. 3), pan-neocortical activation of Sst-Chodl cells increased neocortical LFP power predominantly in low-frequency bands, and independently of behavioural state (Fig. 5j,k and Extended Data Fig. 9j). The effects were prominent in the neocortex but minimal in the hippocampus (Extended Data Fig. 9i–k), indicating a preferential neocortical engagement. Notably, treating control mice lacking DREADD expression with CNO did not alter their sleep–wake architecture (Extended Data Fig. 9l–o).

Because the original behavioural experiments were performed during the light (inactive) phase, when mice naturally spend more time asleep, we next tested whether activation of Sst-Chodl cells would have similar effects during the dark (active) phase (Fig. 5l and Extended Data Fig. 10). Indeed, mice that were treated with CNO during the dark (active) phase exhibited increased SWS duration and reduced sleep latency, compared with vehicle-treated mice (Extended Data Fig. 10b–g). Notably, the amounts of sleep induced by Sst-Chodl cell activation during the dark (active) phase were comparable to—and in some cases exceeded—baseline levels of sleep observed during the light (inactive) phase of vehicle-treated mice (Fig. 5m), indicating a robust sleep-promoting effect.

Together, these results indicate that, independent of circadian phase, global activation of neocortical Sst-Chodl cells is sufficient to promote cortical synchrony and sleep.

## Discussion

Here we show that long-range, neocortical Sst-Chodl cells—a unique neuronal subtype^7,9^ that is highly conserved across evolution^12^—are selectively active during low-arousal, synchronized states and promote patterns of synchronous neocortical activity. We propose that Sst-Chodl cells synchronize neocortical networks through their extensive local and long-range projections, positioning them as a key cortical component in the regulation of sleep.

The prevailing framework for sleep regulation emphasizes subcortical structures as primary drivers of sleep initiation and maintenance, with the cortex viewed mainly as a passive downstream recipient^30^. However, accumulating evidence supports a more active role for the neocortex in sleep–wake regulation. Our findings extend this framework by identifying Sst-Chodl cells as a distinct cortical population capable of shaping neocortical synchrony and sleep-related dynamics. In this context, we find that Sst-Chodl cells exhibit exceptionally dense projections and high synaptic connectivity across all cortical layers in the cortical region in which their cell bodies are located, making them well suited to promoting local cortical synchrony. This feature could be particularly relevant for forms of local sleep, in which circumscribed cortical regions exhibit sleep-like patterns in the absence of overt behavioural sleep^31^. In addition, we show that Sst-Chodl cells extend long-range axons that inhibit neurons millimetres away from their somata, suggesting a mechanism by which these cells contribute to synchronization across distant neocortical regions^32^. Notably, we find that Sst-Chodl neurons provide particularly strong synaptic inhibition and LFP modulation in deep cortical layers, in which pyramidal neurons associated with synchronized states are concentrated^33,34^. Pyramidal neurons in these layers have been shown to contribute to sleep regulation^22^, raising the possibility that Sst-Chodl cells influence sleep homeostasis by shaping the activity of deep-layer cortical circuits. Future studies will be required to determine whether Sst-Chodl cells outside sensory cortices receive inputs from, or project to, canonical sleep-promoting regions in the hypothalamus or midbrain, or whether their influence on sleep is mediated mainly through intracortical mechanisms.

Although cortical Sst-expressing INs as a group have been implicated in the regulation of cortical states and linked to sleep-related processes^20,21^, their functional roles have been difficult to reconcile across studies, with reported effects spanning gamma^35,36^, beta^37^, theta^38^ and delta^20^ rhythms. Single-cell transcriptomic profiling has revealed substantial heterogeneity in the Sst population, indicating that distinct Sst subclasses subserve divergent, and potentially opposing, functions^8,29^. In this context, differences in experimental approaches might preferentially engage different Sst subtypes, contributing to variability in reported outcomes. Consistent with this interpretation, we identified a minor population of cells within the *Sst-Nos1* intersection that were anti-correlated with arousal and probably correspond to non-Sst-Chodl, type II Nos1 cells expressing low levels of SST and nNOS (refs. ^8,26^). These cells might participate in neurovascular control during movement^39,40^, consistent with a functional role distinct from the promotion of synchronized cortical states. Our results highlight the importance of transcriptomically informed targeting strategies for dissecting IN subclass function and underscore the utility of this approach for understanding cortical-circuit mechanisms.

Beyond their synaptic inhibitory effects, Sst-Chodl cells express other signalling molecules that could contribute to their function. Notably, these neurons are likely to represent the largest neuronal source of nitric oxide in the neocortex^39,41^. Mice knockout for *Nos1* exhibit disrupted sleep architecture, including a diminished ability to sustain prolonged SWS bouts and an impaired homeostatic response to sleep deprivation^14^. Similarly, selective deletion of *Nos1* from Sst cells results in deficits in delta rhythms^42^. Potential targets of nitric oxide signalling from Sst-Chodl cells include parvalbumin-expressing INs^41,43^ and components of the neurovascular system^39^. The slow, synchronized electrical activity promoted by Sst-Chodl cell activation might therefore be mirrored in vascular dynamics, potentially supporting rhythmic fluid movement during glymphatic clearance in sleep^44^.

The mechanisms that govern the state-dependent activation and suppression of Sst-Chodl cells remain to be elucidated. Although thalamocortical circuits are well known to regulate cortical UP and DOWN state dynamics^1,30^, our monosynaptic tracing experiments indicate that Sst-Chodl cells receive predominantly neocortical inputs, with minimal thalamic contributions. Our findings show that the activity of Sst-Chodl cells increases before DOWN state onset, which suggests that these neurons participate in regulating UP–DOWN transitions, similar to other IN classes^45–47^. Activation of Sst-Chodl cells could arise through disinhibition mediated by VIP-expressing INs, consistent with our rabies tracing data, or through changes in arousal-related neuromodulatory tone, such as acetylcholine^48^. This interpretation is consistent with previous studies showing that Sst-Chodl cells receive extensive neuromodulatory inputs^7,29,41,48^ which might not be captured by our rabies-based tracing approaches^7,41,48,49^. Another possibility is that sleep-promoting substances (somnogens), such as adenosine, could activate these cells during synchronized cortical states^30^. In addition, substance P has been shown to activate Sst-Chodl cells, and local application of substance P promotes cortical slow-wave activity and electrophysiological signatures of SWS^50,51^, although its precise role in sleep regulation remains unclear. In this context, we find that Sst-Chodl cells receive input from both PV- and SST-expressing INs, which represent potential sources of substance P. Identifying additional G-protein-coupled receptors expressed by Sst-Chodl neurons will be important to elucidate the signalling pathways through which sleep- and arousal-related factors engage this cell population.

Overall, this work identifies Sst-Chodl cells as a key cortical circuit element associated with low-arousal neocortical states and sleep. The properties of this genetically distinct IN subtype indicate that cortical circuits make an active contribution to sleep-related dynamics, complementing the established roles of subcortical structures in sleep regulation. Together with previous studies^16–25^, our findings highlight an underappreciated role of the cortex in shaping sleep states. Given that sleep disturbances are a prominent feature of many neuropsychiatric, neurodevelopmental and neurological disorders, this highly conserved cell population could represent a useful entry point for future mechanistic and translational investigations^25^.

## Methods

### Mice

All mouse handling and maintenance was performed according to the regulations of the Institutional Animal Care and Use Committee of the Albert Einstein College of Medicine (protocol 00001393). *Sst*^*flp*+/+^; *Nos1*^*creER*+/−^ and *Sst*^*flp*+/−^;*Nos1*^*creER*+/−^ mice were used interchangeably in this study, with no differences detected between the two groups (*Nos1*^*creER*^, Jax 014541; *Sst*^*flp*^, Jax 031629). *Nos1*^*creER*+/−^ mice were crossed with *Sst*^*flp*+/+^ mice to obtain *Sst*^*flp*+/−^;*Nos1*^*creER*+/−^ mice and *Sst*^*flp*+/−^;*Nos1*^*creER*+/+^ were crossed with *Sst*^*flp*+/+^ mice to obtain *Sst*^*flp*+/+^;*Nos1*^*creER*+/−^ mice. *Sst*^*flp*+/+^;*Nos1*^*creER*+/−^ mice were crossed with *Ai210*^+/+^ mice (a gift from the Allen Institute)^52^ to obtain mice for in vivo imaging experiments. *Chodl*^*cre*+/+^ mice (a gift from the Allen Institute)^52^ were crossed with* Sst*^*flp*+/+^ or *Sst*^*flp*+/−^ mice. Adult male and female mice aged 50 days or older were used in this study. Mice were kept under a 12 h light–dark cycle (lights on 07:00 or lights on 16:00 for ZT18 chemogenetic experiments) under standard housing conditions (20–24 °C and 30–70% humidity).

### Cell type classification and sequencing

Data were retrieved from a previous study^52^.

### Abbreviations of brain regions

ACA, anterior cingulate area (d, dorsal); AIp, agranular insular area, posterior; APr, area prostriata; Aud, auditory cortical area (d, dorsal; p, primary; po, posterior; v, ventral); AuT, auditory and temporal cortical area; BS, brainstem; CB, cerebellum; CLA, claustrum; ECT, ectorhinal area; ENT, entorhinal area (l, lateral; m, medial dorsal); EPd, endopiriform nucleus, dorsal; FrM, frontomotor cortex; HPF, hippocampal formation; HVAs, higher visual areas; Mo, motor cortical area (p, primary; s, secondary); NC, neocortex; OB, olfactory bulb; Olf, olfactory areas; PAR, parasubiculum; PERl, perirhinal area; PIR, piriform area; POST, postsubiculum; PRE, presubiculum; RSP, retrosplenial cortical area (agl, lateral agranular; d, dorsal; v, ventral); RSPagl, retrosplenial area, lateral agranular; RSPd, retrosplenial area, dorsal; S1, primary somatosensory cortex; SS, somatosensory cortical area (bfd, barrel field; ll, lower limb; m, mouth; p, primary; ul, upper limb; tr, trunk; un, unassigned; s, supplemental); StP, striatum–pallidum; SUB, subiculum; TEa, temporal association area; V1, primary visual cortex; VIS, visual area (a, anterior; al, anterolateral; am, anteromedial; l, lateral; li, laterointermediate; p, primary; pl, posterolateral; pm, posteromedial; por, postrhinal; rl, rostrolateral); VISC, visceral area.

### Surgical procedures

Mice were anaesthetized with isoflurane (5% by volume for induction and 1–2% for maintenance), placed on a stereotaxic frame and kept warm with a closed-loop heating pad. For pain management, mice were given intraperitoneal (i.p.) meloxicam at 2.5 mg kg^−1^ and local lidocaine on the scalp before skin incision. The following coordinates are distance (in mm) from bregma and dorsoventral (DV) values refer to brain surface.

For viral injections, we minimized brain damage by performing burr holes, keeping a thin layer of the bone intact where the glass micropipettes could penetrate.

For wide-labelling morphological reconstructions, 50 nl viral vector was injected into a burr hole split across two levels (DV −0.25 and DV −0.55) at anteroposterior (AP) −3.3, mediolateral (ML) ±2.7 to target primary visual cortex (V1); or at AP −1, ML ±3 to target primary somatosensory cortex (S1).

For silicon probe-coupled optogenetic experiments, four burr holes were drilled centred over V1 (AP −3.3, ML ±2.7) spaced around 1 mm apart, and positioned to avoid blood vessels visible through the skull. Virus (500 nl) was injected into each burr hole split across two levels (DV −0.25 and DV −0.55) for a total of 2 µl virus.

For calcium imaging-related surgeries, mice were also administered dexamethasone at 4 mg kg^−1^ 1 h before craniotomy. A 3-mm craniotomy was made over V1, and, if applicable, virus was injected into the centre of this craniotomy while the brain was kept moist with hydrated gelatin surgical foam. Then a cranial window was placed over the opening and sealed to the skull with cyanoacrylate glue. For imaging Sst-Chodl cells in superficial layers, the cranial window consisted of a stack of three coverslips: two 3-mm #1 glass coverslips (Warner Instruments) attached with Norland Optical Adhesive 71 to a 5-mm glass coverslip, allowing the 3-mm glass to sit against the dura to discourage bone regrowth^54^. For imaging Sst-Chodl cells in deep layers, durotomy was performed before implantation of a 1.5-mm microprism (A1 coated, 4531-0023, Tower Optical) attached with Norland Optical Adhesive to a single 3-mm glass coverslip.

For pan-neocortex viral delivery, 22 burr holes were split bilaterally and 400 nl of viral vectors was injected at the following coordinates: AP +1, ML ±3, DV −1; AP +1, ML ±1.8, DV −0.4; AP −0.5, ML ±3.8, DV −1.5; AP −0.5, ML ±2.6, DV −0.4; AP −2, ML ±4, DV −1.2; AP −2, ML ±3.2, DV −0.3; AP −2, ML ±1.2, DV −0.3; AP −3.5, ML ±3.5, DV −0.3; and AP −3.5, ML ±2, DV −0.3; and 800 nl of viral vectors was injected at the following coordinates: AP 2.5, ML ±1.2, DV −1; and AP 0, ML ±1, DV −0.5.

For rabies tracing experiments, a total volume of 500 nl of the helper mixture (2:1 ratio optimized glycoprotein over avian tumour virus receptor A (TVA)) split across two levels (DV −0.25 and DV −0.55) was injected into a single burr hole at AP −3.3, ML ±2.7. After a minimum of four weeks to allow robust expression of the helper constructs, rabies vector was delivered as a single injection of 150 nl at the same AP–ML coordinates and DV −0.4. Mice were perfused 10 days after rabies delivery to capture the full extent of monosynaptic retrograde labelling while minimizing cytotoxicity.

For calcium imaging and whole-neocortex chemogenetic experiments, a separate burr hole was made in contralateral above V1 and a made-in-house microwire array connected to a 16-channel omnetic (A79038-001, Omnetics Connector), used for measuring LFPs, was inserted. The LFP wires consisted of five to eight tungsten wires, 50 µm in diameter, spanning from the middle layers of V1 area into cornu ammonis area 1 (CA1). For calcium imaging combined with flexible 32-channel probes (tetrode configuration, NeuralThread^55^), an electrode attached to a stainless steel pole with adhesive polyethylene glycol was inserted perpendicular to the brain at the border of the craniotomy. Then, the polyethylene glycol was dissolved by applying saline continuously for 10 min on top of the pole to ensure its disengagement from the electrode. After pulling the pole straight up out of the brain, the cranial window was positioned above the electrode.

For multi-site LFP and optogenetic experiments, eight burr holes were made at the following coordinates: anterior: AP+ 1.35, ML −2.8 and AP +0.7, ML −1.2; distal: AP +0.05, ML −2.8 and AP −0.6, ML −1.2; medial: AP −1.25, ML −2.8 and AP −1.9, ML −1.2; proximal: −2.55, ML −2.8 and −3.2, ML −1.2. A made-in-house microwire array consisting of eight tungsten wires of 200 µm in diameter, were aligned to the mouse skull, connected to a 16-channel omnetic and inserted in the surface of the brain. Virus (500 nl) was injected ipsilateral of the multi-LFPs at AP −2.7, ML −2.7 split across two levels (DV −0.25 and DV −0.55), and a fibre-optic cannulae of 400-µm diameter and 6-mm length was positioned at the entrance of the burr hole.

Injections were performed using a Nanoject III system at a rate of 1–2 nl s^−1^ through glass micropipettes that were pulled and then ground to bevel with 40-µm diameter using a Naragishe diamond wheel. For LFPs and flexible probes, a tungsten ground wire of 200 µm diameter attached to a gold pin was inserted into the cerebellum, and a headpost was implanted as previously described^56^.

All implants were secured to the skull using Optibond or Super-Bond followed by dental cement. When *Nos1*^*creER*+/−^ mice were used, tamoxifen (Thermo Fisher Scientific) was administered at least 2 weeks after surgery. A stock solution of 20 mg ml^−1^ tamoxifen in corn oil was injected i.p. at 0.1 mg tamoxifen per g body weight for 5 consecutive days. Mice were left for a minimum of 4 weeks for CreER-mediated recombination before experimental use.

The following AAV viral vectors were used in this study at concentrations of 10^12^–10^13^ vg ml^–1^ (written in the format AAV vector, serotype, source):

- Ef1a-CreOn/FlpOn-oScarlet, 8, provided by the laboratory of K.D.
- Ef1a-CreOn/FlpOn-GCaMP6m, 8, provided by the laboratory of K.D.
- hSyn-CreOn/FlpOn-ChR2-EYFP, DJ, UNC Vector Core
- EF1a-fDIO-mCherry, 5, Addgene (114471)
- nEF-CreOn/FlpOn-hM3Dq-mCherry, 8, provided by the laboratory of K.D.
- nEF-CreOn/FlpOn-TVA-mCherry WPRE, 8, Gene Vector and Virus Core (GVVC; AAV-197), Wu Tsai Neurosciences Institute, Stanford University
- Ef1a-CreOn/FlpOn-oG WPRE, 8, GVVC (AAV-198)
- EnvA-DG-Rabies-EGFP, not applicable (N/A), Salk Vector Core
- iSSTe4-ChR2-mCherry, 1, provided by the laboratory of G. Fishell^57^

### Immunohistochemistry

Mice were perfused transcardially with ice-cold 4% paraformaldehyde (PFA) and brains were dissected and post-fixed in 4% PFA for 1 h at 4 °C. After sucrose cryoprotection, brains were embedded in OCT, cryosectioned at 20 µm and adhered to glass slides. For staining, sections were incubated in a blocking solution containing 1.5% donkey serum, 1% Triton-x-100 for 1 h at room temperature. Primary antibodies were applied overnight at 4 °C, followed by washes and 1-h incubation in secondary antibodies. Tissue sections were mounted in Prolong Gold with DAPI (Thermo Fisher Scientific) and imaged on a Zeiss Axioscan slide scanner and a Zeiss LSM 880 confocal microscope for high-resolution imaging.

The following antibodies were used in this study (written in the format antigen target, concentration, vendor, product number):

- Somatostatin (SST), 1:250, Millipore, MAB354
- Somatostatin-14 (SST), 1:1,000, Peninsula, T-4103
- Parvalbumin (PV), 1:1,000, SYSY, 195004
- Vasoactive intestinal peptide (VIP), 1:250, SYSY, 443005
- Neuronal nitric oxide synthase (nNOS), 1:500, Abcam, ab1376
- CTIP2, 1:500, Abcam, ab18465
- SATB2, 1:500, SYSY, 327004
- GFP (reactive against EYFP), 1:500, Invitrogen, A-111222
- RFP (reactive against mCherry), 1:250, Chromotek, 5f8

### Tissue clearing and light-sheet imaging

Mice were prepared as described above for morphological reconstruction. Mice were then transcardially perfused with ice-cold 1× PBS followed by 4% PFA. Brains were dissected and post-fixed in 4% PFA at 4 °C for 24 h. Brains were processed with SHIELD reagents (LifeCanvas Technologies) before beginning active tissue clearing using the SmartBatch+ system (LifeCanvas Technologies) using manufacturer protocols. Tissues were refractive index-matched using EasyIndex (LifeCanvas Technologies) and imaged on a SmartSPIM light-sheet microscope (LifeCanvas Technologies) with a 3.9× magnification objective (NA: 0.2, manufactured by Thorlabs and modified by LifeCanvas Technologies). Data were aligned to the Allen Mouse Brain Common Coordination Framework (CCF) using NeuroInfo (MBF Bioscience).

### Whole-brain sparse labelling and reconstructions

Whole-neuron morphology data were generated using HortaCloud^58^, an open-source, cloud-based neuron reconstruction platform. Morphological reconstructions were performed on gene-defined, sparsely labelled individual neurons imaged across the entire mouse brain using two-photon fluorescent micro-optical sectioning tomography (fMOST)^59,60^. For each neuron, the full local and long-range axonal arbor and dendritic trees were manually identified and traced by an experienced annotator. A second independent annotator then proofread the initial reconstruction and added missed branches and removed incorrectly identified segments. All branch points and terminal endings were verified to ensure complete reconstruction of each neurite. Any discrepancies between annotators were resolved by consensus before the reconstructed cell was deemed final.

### Slice electrophysiology and oIPSCs

Acute coronal slices of the neocortex were prepared from adult *Sst*^*flp*+/+^;*Nos1*^*creER*+/−^ mice of either sex injected with AAV-CreOn/FlpOn-ChR2-EYFP (1 µl split across two depths) (DV −0.25 and DV −0.55) at AP −3.3, ML ±2.7, 5–8 weeks before recording. Mice were anaesthetized with isoflurane and transcardially perfused with ice-cold NMDG-based cutting solution containing (in mM) 92 NMDG, 2.5 KCl, 1.25 NaH_2_PO_4_, 30 NaHCO_3_, 20 HEPES, 25 glucose, 2 thiourea, 5 sodium ascorbate, 3 sodium pyruvate, 0.5 CaCl_2_·2H_2_O and 10 MgSO_4_·7 H_2_O, adjusted to pH 7.3–7.4 with 5 M hydrochloric acid. The brain was dissected rapidly and sectioned at 250–260 µm with a vibratome (VT1200S, Leica), and allowed to recover in the same solution for 5-10 min at 34 °C before transfer to warm artificial cerebrospinal fluid (ACSF) containing (in mM) 120 NaCl, 25 NaHCO_3_, 1.25 NaH_2_PO_4_, 3 KCl, 1 MgCl_2_, 1.5 CaCl_2_, 11 glucose, 3 sodium pyruvate and 1 sodium ascorbate equilibrated with O_2_ and 5% CO_2_. Slices were incubated for another 30 min and then kept at room temperature until recording for up to 8 h. For a subset of experiments, we used a sucrose-based cutting solution containing (in mM) 90 sucrose, 60 NaCl, 5 MgCl_2_, 2.75 KCl, 1.25 NaH_2_PO_4_, 1.1 CaCl_2_, 9 glucose, 26.5 NaHCO_3_, 3 sodium pyruvate, 1 sodium ascorbate, equilibrated with 95% O_2_ and 5% CO_2_. Slices were transferred directly to warm ACSF after cutting under these conditions.

We verified the presence of ChR2-EYFP-expressing fibres in each slice at various distances from the injection site before recording. Putative postsynaptic neurons were recorded across all cortical layers and at various distances from the injection site at near-physiological temperature (around 32 °C) with an internal solution containing (in mM) 15 CsCl, 120 caesium gluconate, 8 NaCl, 10 HEPES, 2 MgATP, 0.3 NaGTP, 0.2 EGTA and 2 mg ml^−1^ biocytin for post-hoc anatomical analysis (pH adjusted to 7.2 with CsOH; osmolarity adjusted to 290 mOsm). The chloride reversal potential was around −44 mV, and GABAergic currents were recorded at a holding potential of 0 mV. Visually guided whole-cell recordings were obtained with patch pipettes of around 3-MΩ resistance pulled from borosilicate capillary glass (BF150-86-10, Sutter Instrument). Electrophysiology data were acquired using a Sutter dPATCH amplifier (Sutter Instrument), digitized at 10 kHz and filtered at 5 kHz. oIPSCs were evoked with 0.5-ms whole-field pulses of blue light (CoolLED) at a frequency of 0.2 Hz. To isolate inhibitory currents in voltage-clamp, the following receptor antagonists were added to the bath solution: 2 µM (R)-CPP, 5  µM NBQX and 1.5 µM CGP to block NMDA, AMPA and GABA_B_ receptors, respectively. To confirm GABAergic identity, oIPSCs were blocked with 50 µM gabazine SR95531 in some experiments. To confirm that oIPSCs were monosynaptic, 0.5 µM TTX and 100 µM 4-AP were included in the bath solution in a subset of experiments. All drugs were purchased from Abcam, Tocris or HelloBio. After recording, the patch pipette was withdrawn slowly to allow resealing of the membrane and slices were fixed in 4% PFA overnight. Biocytin-filled neurons were labelled with streptavidin–Alexa 647 using standard protocols. Confocal *z*-stacks of streptavidin-labelled neurons and EYFP^+^ Sst-Chodl fibres were taken on a Zeiss LSM 880 microscope at 1–2-µm increments. *z*-stacks were processed in ImageJ and their positions in the cortex were recorded.

### Habituation and head restraint

Mice were briefly habituated to handling for several days before surgery. Mice were allowed to recover for 2 days before continuing habituation to handling. After a minimum of 1 week after implantation, mice were gradually exposed to the head-fixation apparatus (https://github.com/janelia-experimental-technology/Rodent-Belt-Treadmill), which consists of a low-friction rodent-driven belt treadmill. The head-fixation duration was gradually increased over 2 weeks until the mice were comfortable with multi-hour head fixation sessions.

To promote sleep while head-fixed, mice were given multiple habituation sessions on the recording rig before data collection. We ensured that the treadmill was oriented horizontally with no slope, with the distance between the head fixation level and the treadmill properly adjusted. The set-up was cleaned thoroughly to minimize odours from other mice, and a radiant heat lamp was provided.

### Videography

Videos of mice were acquired using FlyCapture 2 software, at 30 Hz using Blackfly machine-vision ethernet-enabled cameras (FLIR) equipped with a Basler lens (model c125-1620-5m) for head-fixed experiments and a Computar lens (TG4Z2813FCS-IR 0.33-Inch) for freely moving experiments. Rigs were illuminated with infrared LED arrays, similar to methods described previously^61^. The camera was configured to send transistor–transistor logic (TTL) pulses to synchronize videography with other data streams.

### In vivo two-photon calcium imaging

GCaMP was expressed either using an AAV-CreOn/FlpOn-GCaMP6m viral vector or through transgenic expression in the *Ai210* mouse line (a Cre- and Flp-dependent GCaMP7f). No substantial differences were observed between the two approaches. Mice were imaged on a custom Bergamo two-photon microscope coupled to a Ti:Sapphire laser (Mira 700, Coherent). Emitted light was collected through a 10× 0.5-NA long-working-distance objective (TL10X-2P, Thorlabs). Images were typically acquired with ThorImage software at 1.4 frames per second with a resolution of 512 × 512 pixels and, for experiments coupled with flexible probes, at 14.6 frames per second with a resolution of 128 × 128 pixels. For microprism data, cell positions were estimated from the distance of the soma relative to the visible-damage layer produced by microprism insertion, which was used as a reference for the cortical surface (estimation relative to the Allen Brain Atlas). Data streams were synchronized using TTL pulses collected on an RHD USB interface board (Intan Technologies) at 20 kHz. Before imaging, novel objects were placed in the mouse’s home cage, one per hour for up to 4 hours, to promote exploration and subsequently facilitate sleep during imaging.

### Acute in vivo electrophysiology and optogenetics

Mice were injected with AAV-CreOn/FlpOn-ChR2-EYFP and implanted with headposts as described above. After viral expression, tamoxifen induction and treadmill habituation, mice were administered dexamethasone (4 mg kg^−1^, i.p.) 1 h before craniotomy. An approximately 3-mm craniotomy was made over the injection site (V1).

A 64-channel linear silicon probe (H3 probe, Cambridge NeuroTech) physically coupled to a tapered optical fibre was slowly (1 µm s^−1^, over around 20 min) inserted into V1. The craniotomy was kept moisturized during the recording by placing a small volume of silicon oil on the surface of the brain. State measurements were made as described below. Between recording days, the craniotomy was protected with KWIK-CAST silicon elastomer (World Precision Instruments). Mice were recorded once per day for 3–4 days.

Recordings were split into blocks of around 30-min spontaneous periods (without stimulation) and around 30-minute periods of stimulation. Several types of stimulation were used, including sinusoids (1 Hz, 4 Hz, 10 Hz and 40 Hz), flat pulses and white noise, each delivered for 30 s followed by a 30-s ITI. Blue light was provided by a fibre-coupled LED (MF470F4, Thorlabs). Signals to control LED light intensity were generated using custom-written MATLAB software and delivered to an NI DAQ card (NI-PCIe-6323) to be output to the LED control box (LEDD1B, Thorlabs). No light artefacts were detected in our recordings.

Across more than 700 recorded single units, plus additional multi-units from 10 mice, no optotagged units were found, underscoring the extremely low abundancy of this neuronal population and the difficulty of capturing the cells directly in silicon probe recordings.

Data were acquired using an Intan RHD2000 interface board at 20 kHz.

### Multi-LFPs and optogenetics

Mice were injected with AAV-CreOn/FlpOn-ChR2-EYFP and implanted with headposts as described above. Recordings were split into blocks of around 30-min spontaneous periods (without stimulation) and around 30-min periods of stimulation using a 10-Hz sinusoid frequency. Similar to the acute recordings, the stimulation periods consisted of 30 s of blue light followed by a 30-s ITI. The optical and acquisition hardware used was the same as that described above.

### Freely moving behavioural assay

Mice were injected with AAV nEF-CreOn/FlpOn-hM3Dq-mCherry or control EF1a-fDIO-mCherry, implanted with LFP wires as described above and single-housed after surgery. Mice were habituated to wired tethering via a 16-channel digital headstage with an accelerometer (Intan Technologies) and connected to a motorized commutator (Doric Lenses or Neurotek). To minimize stress and noise distraction, mice were kept in their home cage in an acoustic foam box for 2 h per day for 3–5 days. Mice were also habituated to receiving systemic saline injections at the beginning of the session for at least 2 days before monitoring their behavioural state. A consistent time of day was used for each mouse during the light (inactive) phase (ZT3–ZT7) or during the dark (active) phase (ZT18), and this was maintained throughout the experiment. No differences in sleep behaviour were detected across specific ZT windows used within the light (inactive) phase, but sessions during the light (inactive) and the dark (active) phase were analysed separately.

After habituation, mice received i.p. injections of either vehicle (saline) or CNO (HelloBio) at 0.5 mg kg^−1^. This dose has been shown to have no effect on the sleep of control mice without DREADD treatment^62,63^, and we confirmed this in our control experiment (12 sessions from 6 mice during the light phase, Extended Data Fig. 9l–o). The initial treatment was counterbalanced across mice, and the alternative treatment was administered the following day. Thus, each session was paired with its opposite treatment for analysis (vehicle versus CNO or CNO versus vehicle). Mice completed two vehicle and two CNO sessions, and data for each treatment were averaged across the two sessions. To ensure sufficient wakefulness, only mice that remained awake for at least 60% of the time during vehicle sessions were included in the analysis within the light or dark phase. We obtained a total of 28 sessions during the light phase from 14 mice and 12 sessions during the dark phase from 6 mice.

LFP data were acquired at 1,250 Hz using an Intan RHD2000 interface board.

### Behavioural state scoring

Behavioural state was divided on the basis of a combination of the acquired state measurements. First, to score sleep states we used previously published and validated methods implemented in the Buzcode toolbox from the Buzsaki lab^64,65^. In brief, this method provides automatic state scoring by distinguishing SWS from wake using the slope of the power spectrum (a large slope occurs during high delta periods of sleep). REM sleep is identified as periods of high theta/delta ratio with low EMG. The automatic scoring is then visualized and manually refined by experts. For optogenetic and chemogenetic experiments, state was scored using LFPs that were recorded outside the neocortex, in the hippocampus, to provide an accurate measure of state. All sleep scoring was done blinded to stimulation conditions (optogenetic or chemogenetic).

The remaining periods of wakefulness were then divided into movement and quiet wake (QW) states. In head-fixed recordings, movement intervals were defined as periods of 5 s or longer in which facial motion energy (extracted using Facemap, binned at 1 s) exceeded the 90th percentile of the session’s distribution. QW was defined as the remaining wake intervals, with a minimum duration of 5 s. A similar procedure was applied in freely moving experiments, but accelerometer signals were used instead of the facial motion to classify movement and QW states. We noted that head-fixed mice exhibited substantially longer QW periods than did freely moving mice, and we confirm, consistent with previous reports, that head-fixed mice sleep with their eyes open, and exhibit typical LFP signatures (high delta power during SWS and high theta/delta ratio during REM)^66^. Although episodes of QW, REM and SWS in head-fixed mice might differ in meaningful ways from those recorded in freely sleeping mice, we used the same state terminology across head-fixed and freely moving experiments for consistency.

### Data analysis

Data were analysed using open-source software packages and custom-written MATLAB (2018b–2023b) and Python code.

### Whole-neuron morphology projection matrix

SWC files were merged and resampled so that spacing between nodes was uniform. Merged files were registered to the average mouse brain template of the Allen Mouse Brain CCF (v.3)^67^. CCF-registered reconstructions were translated so that all somas were positioned on the left hemisphere. To generate a projection matrix, we used two strategies: one included all axons present in an area (Fig. 1, Supplementary Table 1 and Extended Data Fig. 1), and the other required that a targeted structure contain at least one branch tip and one node (Supplementary Table 2). All projections were ipsilateral, and targeted structures are reported only for the ipsilateral hemisphere.

### Whole-brain fibre density and rabies

Mice were cryosectioned as described above, with uniform sampling throughout the entire brain. Imaged brain sections were aligned to the Allen Mouse Brain CCF using NeuroInfo (MBF Bioscience), with the BrainMaker workflow. Labelled neuronal arbor in aligned tissue was then reconstructed within single sections using Neurolucida 360 (MBF Bioscience). Density measurements for individual areas were made by calculating the total path length of reconstructed arbor within that area divided by the volume of the area (the two-dimensional area within section multiplied by the section thickness). The coordinates of each identified rabies-positive neuron were compiled to yield a complete whole-brain map for each mouse. To account for variability in labelling efficiency between mice, data from each brain were normalized to the total number of rabies-positive cells identified in that mouse. These normalized values were then used for quantification of layer distributions, regional localization and subregional visual cortex analyses.

### Ex vivo patch-clamp recordings

Electrophysiology data were analysed using SutterPatch v.2.4 (written in IgorPro, Wavemetrics) and AxoGraph v.1.7.6. oIPSCs were aligned to the light stimulus and 20–60 traces were averaged to calculate oIPSC amplitude and latency. Amplitude was measured as the maximum positive peak of the baseline-subtracted oIPSC, and latency was calculated as the time between the light stimulus and crossing a 2 s.d. threshold of the oIPSC from baseline.

### Extraction of calcium activity

Acquired images were processed using Suite2p (ref. ^68^) to extract regions of interest and fluorescence traces. Δ*F*/*F*_0_ values were computed by normalizing fluorescence to a moving 10th percentile within 10-min windows as the baseline (*F*_0_). Deconvolved calcium signals were obtained from Suite2p, and, owing to their higher temporal resolution, were used to examine the temporal relationships between calcium activity, delta power, state transition and DOWN states. To investigate state transition, calcium activity was evaluated only during states lasting a minimum of 10 s. The latency of calcium activation during SWS was defined as the earliest time point at which the deconvolved calcium signal exceeded 2 s.d. above baseline activity. When measuring calcium activity around DOWN states, calcium traces were corrected by subtracting chance-level calcium activity generated by circularly shifting the calcium signal (circshift in Matlab) 100 times within the 1-s window around the onset of DOWN states.

### Arousal score

Arousal score (Fig. 2b) was calculated using the pca function in MATLAB. It corresponds to the time-varying score for the first principal component of the measured state metrics. Principal component loadings were calculated for each individual recording to provide robustness against measurement error for any singular state metric.

### Classification of low-arousal- and high-arousal-correlated cells

Cell types were determined on the basis of the correlation of Δ*F*/*F* traces with state metrics. *k*-means clustering (*n* = 2) was performed on these correlation coefficients. Separately, we calculated the correlation coefficient of the Δ*F*/*F* signal with the arousal score. Cells with negative correlation coefficients (low-arousal-correlated cells) overlapped completely with the *k*-means low-arousal cluster.

### High- versus low-Sst-Chodl cell activity epochs

Δ*F*/*F* traces were averaged within each recording and then *z*-scored. High-activity epochs were defined as periods in which activity was more than 2 s.d. from the mean, and low-activity epochs were defined as those in which activity was less than 0.5 s.d.

### Single units and LFP extraction

Single units were isolated using Kilosort2 (ref. ^69^). Figures contain data from both broad-spiking and narrow-spiking units, because no differences were found between these groups. LFP signals were collected from the microwire array or from silicon probes and were downsampled to 1,250 Hz. EMG signals were obtained by band-pass-filtering the raw signal (more than 200 Hz) followed by a Hilbert transform.

### Calculation of power spectra

Power spectra were calculated using a wavelet-based spectrogram method (Morlet wavelets, five-cycle width). These wavelets were generated for 100 log-spaced frequencies from 1 Hz to 128 Hz. For freely moving optogenetic experiments, changes in power spectra were assessed by comparing the 30-s stimulation periods to the 30-s ITIs. To investigate changes in power spectra across the neocortex as a function of distance from the injection site, we selected, at each distance (proximal to anterior), the channel that showed the largest delta band power increase. This approach was implemented to minimize the confounding effects of asymmetrical projections in which Sst-Chodl fibres might be absent on one side but present on the other at a single distance, given the substantial variation in individual projection patterns (see Fig. 1).

### Determination of cortical depth

As shown previously, high-frequency power (more than 500 Hz) peaks in mid-layer 5 (ref. ^70^). We interpolated between this layer 5 channel and the first channel outside of the brain (1.3 mm probe inserted 1.2 mm into the brain), assuming the remaining distance, without the mechanical deformation of the brain caused by insertion of the probe, to be 700 µm. With these interpolated pseudo-depths, we then defined layer boundaries at 100, 300, 450, 650 and 900 µm.

### Spiking synchrony

The cross-correlation was calculated for each unit pair using default parameters of the CCG.m function from the Buzcode package. The spike times of these units were shuffled, and the cross-correlation was recalculated 100 times to estimate a chance level. The real cross-correlation was then normalized by this chance level to quantify spiking co-activation.

### Phase locking

The Fourier spectrum of the LFP was calculated for each spike from each unit using the ft_spiketriggeredspectrum function from the FieldTrip toolbox. The circular mean of the phase values at each frequency was calculated as the phase locking value. Although this measure is sensitive to spike count, with higher spikes leading to higher phase locking values, we do not see this as an issue, because stimulation leads to slightly lower firing rates compared with baseline.

### DOWN state detection

DOWN states were detected using a previously published method^27^. In brief, the method detects a confluence of a large positive deflection in LFP, a drop in gamma-band power and a sharp drop in the multi-unit firing rate.

### Linear regression model

Deconvolved calcium signals were used to predict the *z*-scored delta amplitude after constructing a time-lagged design matrix spanning a 20-s temporal window, sampled every 0.2 s. The kernel was centred on the present time point, allowing both past and future lags to contribute to the prediction. Ordinary least-squares regression was fitted in a fivefold cross-validation scheme, and model performance was quantified by the cross-validated coefficient of determination (*R*^2^). The resulting regression weights were averaged across folds to obtain a mean temporal kernel describing the influence of calcium activity at different lags on the predicted delta signal.

### Mouse tracking

Cage position and mouse nest area were delimited manually using a compilation of frames extracted every 10 min from each video. Mouse position was detected from the body centroid of the mouse using the DeepLabCut open-source system^71^. The distance moved by the mouse was extracted according to the registered position of the cage. The duration spent in the nest was estimated by calculating the time that the mouse was present in the nest according to the total duration of the experiment.

### Allen Brain Observatory data analysis

Sst-expressing cell state correlation data were retrieved using the Allen SDK and querying the database for all experiments performed with *Sst*^*cre*^ and Cre-dependent GCaMP mice. Pearson’s correlations were calculated between activity traces and state metrics after binning both signals into 3-s windows. Data on the proportions of Sst-Chodl cells within the Sst population were retrieved from a previously published dataset^8^ from the web-based Brain Knowledge Platform (https://knowledge.brain-map.org/data).

### Analysis of previously published data

Data were retrieved from https://doi.org/10.6084/m9.figshare.19448531 (ref. ^29^), and analysed from spontaneous recordings without visual stimulation.

The following software packages were used to analyse the data presented in this paper (written in the format package name, URL, publication):

- AllenSDK, https://github.com/AllenInstitute/AllenSDK, N/A
- Buzcode, https://github.com/buzsakilab/buzcode, N/A
- Facemap, https://github.com/MouseLand/facemap, ref. ^72^
- Suite2p, https://github.com/MouseLand/suite2p, ref. ^68^
- Kilosort2, https://github.com/MouseLand/Kilosort, ref. ^69^
- FieldTrip, https://www.fieldtriptoolbox.org/, ref. ^73^
- DeepLabCut, https://github.com/DeepLabCut/DeepLabCut, ref. ^71^
- Phy v.2.0 beta, https://github.com/cortex-lab/phy, N/A
- SutterPatch v.2.4, https://www.sutter.com/amplifiers/sutterpatch, N/A
- AxoGraph v.1.7.6, https://axograph.com/, N/A
- HortaCloud, https://doi.org/10.1101/2025.03.13.642887, N/A
- MBF Bioscience, https://www.mbfbioscience.com, N/A
- MATLAB v.2018b–2023b, https://www.mathworks.com, N/A

### Statistical analysis

MATLAB (MathWorks, v.2018b–2023b) was used for statistical analysis. No power calculations were used to predetermine sample sizes or to formally assess normality. The sample size (mice, sessions, cells and units) is consistent with similar studies in the field^21,22,39^ and reflects the technical complexity of the manipulations and recordings, and their yield. Chemogenetic experimental mice were randomly assigned to receive CNO or vehicle injection in the first session. Other experimental conditions were defined by recording site, genetic targeting or stimulation protocols independent of the group assignment. Comparisons were performed using two-tailed parametric *t*-test or one-way, two-way (anova1) or repeated-measures ANOVA, with post-hoc Bonferroni corrections for multiple comparisons (unless stated otherwise). Differences in proportions were assessed using Chi-squared tests (chi2cdf). For optogenetic experiments (linear probes, multi-LFPs and patch clamp) and comparison of SWS duration between light and dark sessions, a linear mixed-effects model was implemented in MATLAB (fitlme) and the model significance was assessed by ANOVA, which tests for the contribution of each fixed factor and their interaction while accounting for unequal sample sizes. Values and statistical tests used are reported in the text and data are represented as mean ± s.e.m. unless stated otherwise. Significance was set with α = 0.05 and is represented on graphs as *P < 0.05, **P < 0.01 and ***P < 0.001.

### Reporting summary

Further information on research design is available in the Nature Portfolio Reporting Summary linked to this article.

## Online content

Any methods, additional references, Nature Portfolio reporting summaries, source data, extended data, supplementary information, acknowledgements, peer review information; details of author contributions and competing interests; and statements of data and code availability are available at https://doi.org/10.1038/s41586-026-10876-y.

## Supplementary information

Reporting Summary

Supplementary Table 1 Single-cell projection density of Sst-Chodl neurons in V1 and higher visual areas (HVAs). Summary of axonal projection patterns for individually reconstructed Sst-Chodl cells. For each neuron, the table reports soma location and the total axonal length contained in each neocortical area. All values are from the hemisphere ipsilateral to the soma, as no contralateral projections were detected. This table provides quantitative support for the broad, intracortical, long-range arborization of Sst-Chodl neurons.

Supplementary Table 2 Branching and terminal projection densities of Sst-Chodl neurons. For each reconstructed Sst-Chodl cell, this table reports the neocortical areas that contain both axonal branch points and terminal endings, following conventions from prior anatomical studies^59^. Values include soma location and the total axonal length within each such area. All data are from the hemisphere ipsilateral to the soma, as no contralateral projections were observed. This table highlights the specific regions where Sst-Chodl axons form local arbor and terminal projections.

Supplementary Table 3 Complete list of area projection density map of Sst-Chodl neurons  labelled by intersectional oScarlet AAV injected in V1. This table reports the projection density (axon path length per unit volume) of reconstructed Sst-Chodl cells across all neocortical regions as well as hippocampal formation, defined in the Allen Mouse Brain CCF, in which projections were detected. This provides a full quantitative overview of the long-range intracortical innervation patters of the population of Sst-Chodl cells .

Supplementary Table 4 Presynaptic partner counts of Sst-Chodl neurons across brain areas. This table lists the number of rabies-labelled presynaptic neurons detected in each brain region, annotated according to the Allen Mouse Brain CCF. Counts include all areas in which retrogradely labelled cells were found, providing a comprehensive map of the upstream inputs to Sst-Chodl cells.

Peer Review File

Supplementary Video 1 Whole-brain visualization of Sst-Chodl cells labelled in primary somatosensory cortex (S1). Three-dimensional rendering of Sst-Chodl cells labelled by intersectional oScarlet AAV injected into S1 and imaged in a whole-mount cleared brain. The video illustrates the dense local arborization and long-range intracortical projections of Sst-Chodl cells across neocortical regions.

Supplementary Video 2 Imaging of a DREADD-expressing Sst-Chodl cell before CNO administration. Simultaneous facial videography and two-photon calcium imaging of a neocortical Sst-Chodl cell expressing excitatory DREADD. Pupil diameter (yellow) and calcium activity (red) are shown along with a real-time indicator (blue line). During periods of reduced arousal, reflected by pupil constriction, the Sst-Chodl cell exhibits increased activity, illustrating the natural state dependence of these neurons before chemogenetic activation.

Supplementary Video 3 Chemogenetic activation disrupts the normal arousal–activity relationship in Sst-Chodl cells. Simultaneous facial videography and two-photon imaging of a neocortical Sst-Chodl cell expressing excitatory DREADD (same cell as in Supplementary Video 2) after systemic CNO administration. Pupil diameter (yellow) and calcium activity (red) are shown along with a real-time indicator (blue line). After CNO injection, the cell becomes persistently active, and the typical coupling between low arousal (pupil constriction) and increased Sst-Chodl cell activity is abolished, demonstrating effective chemogenetic activation.

## Source data

Source Data Fig. 1

Source Data Fig. 2

Source Data Fig. 3

Source Data Fig. 4

Source Data Fig. 5

Source Data Extended Data Fig. 1

Source Data Extended Data Fig. 2

Source Data Extended Data Fig. 3

Source Data Extended Data Fig. 4

Source Data Extended Data Fig. 5

Source Data Extended Data Fig. 6

Source Data Extended Data Fig. 7

Source Data Extended Data Fig. 8

Source Data Extended Data Fig. 9

Source Data Extended Data Fig. 10

## Data Availability

Data underlying all figures are provided with this paper. We also analysed two publicly available datasets: the Allen Brain Observatory (https://knowledge.brain-map.org/data) and the dataset described in ref. ^29^ (https://doi.org/10.6084/m9.figshare.19448531). The raw data generated during this study comprise large multimodal datasets, including two-photon calcium imaging recordings, light-sheet microscopy datasets, in vivo electrophysiological recordings, electroencephalogram and EMG recordings, behavioural videos and associated metadata. Owing to the size and heterogeneous nature of these datasets, and the ongoing consolidation of laboratory data after the transfer of the laboratory between institutions, the raw data are currently undergoing curation and standardization. The complete raw datasets are available from the corresponding authors upon reasonable request for the purpose of reproducing or extending the findings reported in this study. Source data are provided with this paper.

## Code availability

Data processing and analysis were performed using a combination of publicly available software packages, including Suite2p, together with custom MATLAB and Python scripts. General-purpose custom analysis scripts are available through the laboratory’s GitHub repository (https://github.com/Batista-BritoLab/Ratliff-Terral-et-al.-2026---Nature). Additional project-specific scripts used to generate the results reported in this study are available from the corresponding authors upon reasonable request. Owing to their dependence on the organization of the raw datasets and intermediate processing steps, assistance with implementation and use of these scripts will be provided upon request.
