# Supplementary material for: Neocortical long-range inhibition promotes cortical synchrony and sleep
Source: Nature. 2026 Sep 9;658(8135):442–53. doi: 10.1038/s41586-026-10876-y (PMC13645642; doi:10.1038/s41586-026-10876-y)
Supplement: Supplementary file 1 [file 41586_2026_10876_MOESM1_ESM.pdf]

nature portfolio

Corresponding author(s): Renata Batista-Brito
Geoffrey Terral

Last updated by author(s): Jun 24, 2026

Reporting Summary

Nature Portfolio wishes to improve the reproducibility of the work that we publish. This form provides structure for consistency and transparency in reporting. For further information on Nature Portfolio policies, see our Editorial Policies and the Editorial Policy Checklist.

Statistics

For all statistical analyses, confirm that the following items are present in the figure legend, table legend, main text, or Methods section.

n/a	Confirmed	
☐	☒	The exact sample size (*n*) for each experimental group/condition, given as a discrete number and unit of measurement
☐	☒	A statement on whether measurements were taken from distinct samples or whether the same sample was measured repeatedly
☐	☒	The statistical test(s) used AND whether they are one- or two-sided<br>*Only common tests should be described solely by name; describe more complex techniques in the Methods section.*
☒	☐	A description of all covariates tested
☐	☒	A description of any assumptions or corrections, such as tests of normality and adjustment for multiple comparisons
☐	☒	A full description of the statistical parameters including central tendency (e.g. means) or other basic estimates (e.g. regression coefficient) AND variation (e.g. standard deviation) or associated estimates of uncertainty (e.g. confidence intervals)
☐	☒	For null hypothesis testing, the test statistic (e.g. *F*, *t*, *r*) with confidence intervals, effect sizes, degrees of freedom and *P* value noted<br>*Give P values as exact values whenever suitable.*
☒	☐	For Bayesian analysis, information on the choice of priors and Markov chain Monte Carlo settings
☒	☐	For hierarchical and complex designs, identification of the appropriate level for tests and full reporting of outcomes
☒	☐	Estimates of effect sizes (e.g. Cohen's *d*, Pearson's *r*), indicating how they were calculated

Our web collection on statistics for biologists contains articles on many of the points above.

Software and code

Policy information about availability of computer code

Data collection	In vivo calcium imaging data was acquired using ThorImage 4.3 software. Facial video was collected using FlyCapture 2 from Teledyne/FLIR. Custom written code was used to provide optogenetic stimulation of varying shapes. A customized version of the RHD2000 interface was used to collect in vivo ephys data (https://github.com/SjulsonLab/intan_neurosuite). In vitro ephys data were acquired using a dPatch amplifier and a SutterPatch software from Sutter Instruments
Data analysis	Bulk arbor reconstruction and rabies data was analyzed using MBF Bioscience tools including NeuroInfo and Neurolucida 360. Single-cell anatomical reconstruction were annotated with HortaCloud (https://doi.org/10.1101/2025.03.13.642887). Allen Brain Observatory data were analyzed using allensdk (https://github.com/AllenInstitute/AllenSDK). In vivo imaging data was analyzed using Suite2p (https://github.com/MouseLand/suite2p) and custom written scripts on Matlab 2018b-2023b. Facial motion was analyzed using Facemap (https://github.com/MouseLand/facemap). In vivo electrophysiology was analyzed using several software packages including Kilosort2 (https://github.com/MouseLand/Kilosort) and Phy2 (https://github.com/cortex-lab/phy) for spike sorting in addition to fieldtrip (https://github.com/fieldtrip/fieldtrip), buzcode (https://github.com/buzsakilab/buzcode), and custom written Matlab code. Animal tracking was done with deeplabcut (https://github.com/DeepLabCut/DeepLabCut). In vitro ephys data were analyzed using the SutterPatch 2.4 analysis software and Axiograph 1.7.6.

For manuscripts utilizing custom algorithms or software that are central to the research but not yet described in published literature, software must be made available to editors and reviewers. We strongly encourage code deposition in a community repository (e.g. GitHub). See the Nature Portfolio guidelines for submitting code & software for further information.

Data

Policy information about availability of data

All manuscripts must include a data availability statement. This statement should provide the following information, where applicable:

- Accession codes, unique identifiers, or web links for publicly available datasets
- A description of any restrictions on data availability
- For clinical datasets or third party data, please ensure that the statement adheres to our policy

Data processing and analysis were performed using a combination of publicly available software packages, including Suite2p, together with custom MATLAB and Python scripts. General-purpose custom analysis scripts are available through the laboratory's GitHub repository (https://github.com/Batista-BritoLab/Ratliff-Terral-et-al.-2026---Nature). Additional project-specific scripts used to generate the results reported in this study are available from the corresponding author upon reasonable request. Owing to their dependence on the organization of the raw datasets and intermediate processing steps, assistance with implementation and use of these scripts will be provided upon request.
Source data underlying all figures are provided with this paper. This study also analyzed two publicly available datasets: the Allen Brain Observatory (https://knowledge.brain-map.org/data) and the dataset described in Ref.29 (https://doi.org/10.6084/m9.figshare.19448531).
The raw data generated during this study comprise large multimodal datasets, including two-photon calcium imaging recordings, light-sheet microscopy datasets, in vivo electrophysiological recordings, EEG/EMG recordings, behavioral videos, and associated metadata. Owing to the size and heterogeneous nature of these datasets, and the ongoing consolidation of laboratory data following the transfer of the laboratory between institutions, the raw data are currently undergoing curation and standardization. The complete raw datasets are available from the corresponding author upon reasonable request for the purpose of reproducing or extending the findings reported in this study.

Research involving human participants, their data, or biological material

Policy information about studies with human participants or human data. See also policy information about sex, gender (identity/presentation), and sexual orientation and race, ethnicity and racism.

Reporting on sex and gender	n/a
Reporting on race, ethnicity, or other socially relevant groupings	n/a
Population characteristics	n/a
Recruitment	n/a
Ethics oversight	n/a

Note that full information on the approval of the study protocol must also be provided in the manuscript.

Field-specific reporting

Please select the one below that is the best fit for your research. If you are not sure, read the appropriate sections before making your selection.

☒ Life sciences ☐ Behavioural & social sciences ☐ Ecological, evolutionary & environmental sciences

For a reference copy of the document with all sections, see nature.com/documents/nr-reporting-summary-flat.pdf

Life sciences study design

All studies must disclose on these points even when the disclosure is negative.

Sample size	The sample size (animals/sessions/cells/units) is consistent with similar studies in the field (e.g., Krone et al., Nat. Neurosci. 2021; Tossell et al., Nat. Neurosci. 2023; Ruff et al., Cell Rep. 2024) and reflects the technical complexity of the manipulations, recordings, and their yield.
Data exclusions	In vivo electrophysiology sessions were excluded if no single units were recorded. In vivo imaging data was excluded when no transients were located in the data. For chemogenetic experiments, to ensure sufficient wakefulness (in paired Vehilve session) to assess the effects of the CNO on sleep, only animals that remained awake for at least 60% of the time during Vehicle sessions were included in the analysis within light/dark phase. A total of 2 animals during the light/inactive phase and 1 animal during the dark/active were excluded from figure 5 c-k, Extended Data figure 9-10, but all data were included in figure 5m (as indicated in figure legend). For single cell reconstruction, axon targets were excluded from the projection matrix when we could not reliably verify its registration.
Replication	Acute in vivo optogenetic results were replicate across 2 cohorts of animals. Multi-LFPs with optogenetic results were conducted across 2 cohorts of mice. In vivo imaging was conducted first in Sst-Flp ; Nos1-CreER mice then replicated in Sst-Flp ; Chodl-Cre mice with a total of 11 and 9 independent experiments, respectively. Freely moving chemogenetics results were replicated across two cohorts of animals, first conducted in Sst-Flp ; Nos1-CreER mice then replicated in both Sst-Flp ; Nos1-CreER and Sst-Flp ; Chodl-Cre mice. All attempts were successful at replication.

Randomization	Chemogenetic experimental mice were randomly counterbalanced to receive CNO or vehicle injection on the first session. Other experimental conditions were defined by recording site, genetic targeting or stimulation protocols independent of the group assignment.
Blinding	All sleep scoring was done blinded to stimulation conditions (optogenetic/chemogenetic)

Reporting for specific materials, systems and methods

We require information from authors about some types of materials, experimental systems and methods used in many studies. Here, indicate whether each material, system or method listed is relevant to your study. If you are not sure if a list item applies to your research, read the appropriate section before selecting a response.

Materials & experimental systems

n/a	Involved in the study
☐	☒ Antibodies
☒	☐ Eukaryotic cell lines
☒	☐ Palaeontology and archaeology
☐	☒ Animals and other organisms
☒	☐ Clinical data
☒	☐ Dual use research of concern
☒	☐ Plants

Methods

n/a	Involved in the study
☒	☐ ChIP-seq
☒	☐ Flow cytometry
☒	☐ MRI-based neuroimaging

Antibodies

Antibodies used	rat anti-somatostatin (Millipore, MAB354), rabbit anti-somatostatin (Penninsula, T-4103), goat anti-Nos1 (Abcam, ab1376), rabbit anti-GFP (Invitrogen, A-111222), mouse anti-RFP (Chromotek, 5f8), rat anti-Ctip2 (Abcam, ab18465), guinea pig anti-Satb2 (Sysy, 327004), guinea pig anti-paravalbumin (Sysy, 195004), guinea pig anti-vasoactive intestinal peptide (Sysy, 443005)
Validation	All antibodies were tested with serial dilutions to test for off target staining. The monoclonal (YC7) rat anti-somatostatin (RRID: AB_2255365) and polyclonal rabbit anti-somatostatin-14 (RRID: AB_518614) are both validated for immunohistochemistry (IHC) according to the manufacturers, although mouse is not explicitly listed as a reactive species. Their use in mouse tissue is supported by anatomical specificity and staining patterns consistent with known distributions of somatostatin-expressing interneurons (DOI: 10.1016/j.neuron.2023.11.013). The goat polyclonal anti-nNOS (RRID: AB_300614) is validated by the manufacturer for immunocytochemistry in human samples; however, its use in mouse IHC is supported by evidence showing complete overlap with a mouse-reactive anti-nNOS antibody from Invitrogen (DOI: 10.1093/cercor/bhx102). The following antibodies are reported by their manufacturers to be validated for IHC in mouse tissue: rabbit polyclonal anti-GFP (RRID: AB_221569), rat monoclonal (5F8) anti-RFP (RRID: AB_2336064), rat monoclonal (25B6) anti-Ctip2 (RRID: AB_2064130), guinea pig polyclonal anti-SATB2 (RRID: AB_2620070), guinea pig polyclonal anti-VIP (RRID: AB_2832228) and guinea pig polyclonal anti-Parvalbumin (RRID: AB_2156476).

Animals and other research organisms

Policy information about studies involving animals; ARRIVE guidelines recommended for reporting animal research, and Sex and Gender in Research

Laboratory animals	Sstflp +/+ ; Nos1creER +/- and Sstflp +/- ; Nos1creER +/- animals were used interchangeably in this study with no differences detected between the two groups (Nos1creER: Jax # 014541, Sstflp: Jax # 031629). Nos1creER +/- animals were crossed with Sstflp +/+ animals to obtain Sstflp +/- ; Nos1creER +/- animals and Sstflp +/- ; Nos1creER +/+ were crossed with Sstflp +/+ animals to obtain Sstflp +/+ ; Nos1creER +/- animals. Sstflp +/+ ; Nos1creER +/- animals were crossed with Ai210 +/+ animals (gift from Allen Institute), to obtain animals for in vivo imaging experiments. Chodlcre +/+ animals (gift from Allen Institute) were also used for in vivo imaging experiments and crossed with Sstflp +/+ or Sstflp +/- animals. Both adult male and female mice above P50 were used in this study.
Wild animals	No wild animals were used in the study
Reporting on sex	This study uses both male and female animals. No differences were observed between these groups.
Field-collected samples	No field collected samples were used in the study.
Ethics oversight	All animal handling and maintenance was performed according to the regulations of the Institutional Animal Care and Use Committee of Albert Einstein College of Medicine (Protocol # 00001393).

Note that full information on the approval of the study protocol must also be provided in the manuscript.

Plants

Seed stocks	n/a
Novel plant genotypes	n/a
Authentication	n/a
