# Supplementary material for: Neocortical long-range inhibition promotes cortical synchrony and sleep
Source: Nature. 2026 Sep 9;658(8135):442–53. doi: 10.1038/s41586-026-10876-y (PMC13645642; doi:10.1038/s41586-026-10876-y)
Supplement: Supplementary file 2 [file 41586_2026_10876_MOESM6_ESM.pdf]

nature portfolio

Peer Review File

Neocortical long-range inhibition promotes cortical synchrony and sleep

Corresponding Author: Dr Renata Batista-Brito

Any redactions in this file are there to maintain patient confidentiality, the confidentiality of unpublished data, or to remove third-party material.

This file contains all reviewer reports in order by version, followed by all author rebuttals in order by version.

Attachments originally included by the reviewers as part of their assessment can be found at the end of this file.

Version 0:

Reviewer comments:

Referee #1

(Remarks to the Author)
Current dominant theories of sleep propose that it is regulated by the activity of subcortical nuclei with widespread connections with the neocortex and the rest of the brain. However, recent evidence suggest that the neocortex can also play a leading role in sleep state regulation. This work follows that direction to tackle an important and unsolved question, whether there are specialized cortical cell types that regulate the level of cortical network synchrony and promote sleep. A hallmark of non-REM (NREM) sleep are the highly synchronized cortical dynamics that fluctuate at ~1Hz between states of high (UP) and low (DONW) activity. This manuscript reports a previously unknow cellular mechanism that promotes widespread cortical synchronization. The authors identified a specific subset of interneurons in the mouse primary visual cortex (V1), within the broad family of somatostatin expressing cells, termed Sst-Chodl cells, with dense local and long-range cortical projections. Sst-Chodl cells were preferentially active during periods of low arousal and cortical synchronization, unlike many other interneuron subtypes. Remarkably opto- and chemogenetic activation of Sst-Chodl cells induced cortical synchronization and promoted sleep (by reducing the latency and increased the duration of NREM episodes). The manuscript features an impressive combination of different experimental approaches to thoroughly characterize the properties of Sst-Chodl cells at the morphological, physiological and behavioral levels. Experimental and analytical methods are sound, and the results presented support the author's interpretation on the causal role Sst-Chodl cells in promoting high cortical synchrony. Overall, this is a rigorous and comprehensive study that shines new light into a poorly understood aspect of sleep, the contribution of local cortical mechanisms, and pinpoints a sparse and highly homogenous cell type as a novel key element in this process.

• One of the main hypotheses of the paper is that Sst-Chodl cells play a key role in promoting NREM sleep (or Slow Wave Sleep). The data provided (for example in Fig. 2) clearly supports that Sst-Chodl cells are preferentially active during low arousal states. Fig. 2d suggests that the increase in activity of Sst-Chodl cells is delayed respect the onset of SWS. How do the authors interpret this observation in relation to their proposed functional role? Could Sst-Chodl cells role be to promote the initiation of synchronized states (i.e. transition from REM to SWS) or rather the maintenance of SWS state? A more sensitive analysis to further explore this question would be to calculate the latency to activation from SWS onset for each Sst-Chodl cell. Another could be to use a time-resolved GLM, or other similar approach, to test the predictive power of Sst-Chodl activity on different state transitions (i.e., does the change in Sst-Chodl population activity predict the onset or offset of SWS episodes?). Likewise, it would be possible to test whether Sst-Chodl activity is correlated with the maintenance of synchronized states in a similar manner. For example, is the level of Sst-Chodl activity correlated with/ is predictive of the duration of SWS bouts? Similar analysis for stillness vs movement could also be useful (and some were already provided) but in that case the interpretation is more complicated due to other contributing factors.

• An analysis that would strengthen the uniqueness of the activation pattern of Sst-Chodl cells is to calculate their correlation with other cell types. This can be done in the Allen dataset by calculating cross-correlograms of putative Sst-Chodl cells against the rest of the population. This may show a strong anti-correlation of activity, while doing the same analysis with other Sst cells (or other cell types) will show a positive correlation.

• Were Sst-Chodl cells identified in the silicon probe recording experiments (through optogenetic-tagging)? This would offer the possibility to provide important additional characterization of these cells, including their firing, burstiness and waveform properties. The analysis mentioned in the previous point will be even more convincing using opto-tagged Sst-Chodl cells.

• SWS is not a homogenous state but rather consists in the alternation between periods of high (UP) and low (DOWN) activity. The authors briefly mention this in Fig. 3 but surprisingly did not perform any analysis of Sst-Chodl cells activity in relation to this fundamental feature of SWS. Such analyses are needed to better understand the role this cell type. Since delta waves (reflecting UP-DOWN transitions) were detected together with 2-photon imaging, it would be possible to analyze Sst-Chodl activity in relation to this two substates: are Sst-Chodl active durign DOWN states contrary to most other cortical cells?, what is the timing of activation of Sst-Chodl cells respect to the DOWN to UP / UP to DOWN transition?, does the activity of Sst-Chodl predict the onset of duration of a subsequent DOWN state?

• To better understand the network effect of optogenetic stimulation of Sst-Chodl cells the authors should perform peri-stimulation analysis rather than just analyze the changes between stimulation and non-stimulation blocks. That this, they can plot LFP power changes across the depth profile triggered on stimulation pulses, peri-pulse firing histograms (PSTH), etc. If discrete (e.g., single square pulses) pulses were not delivered, similar analysis could be performed with the phase of sinusoidal stimulation pulses. Some of these analyses can also be done with the freely moving EEG/ LFP recordings, which are currently only minimally analyzed.

• Optogenetic stimulation of Sst-Chodl cells increases the spike coupling to delta waves (Fig. 3g-h). However, the current analyses do not allow to evaluate whether similar or opposite changes happen with other frequency bands. This may be particularly relevant for the high gamma-frequency band (~90-150 Hz) characteristic of deep cortical layers, the same that show the strongest changes in delta power. Both gamma and delta power are associated with UP-DOWN states. Did the stimulation affected the spiking structure of these states (e.g., unit firing timing within UP states, cell participation and magnitude of activation withing UP states, suppression of firing during DOWN states, etc.).

• The authors report minimal changes in firing rates due to optogenetic stimulation of Sst-Chodl, but were the spiking dynamics on individual cells affected? This can be evaluated by computing the coefficient of variation of single unit spike trains, the propensity of bursting, etc. Was the excitatory/ inhibitory balance affected?

• An accurate way of determined cortical layers is by calculating delta wave-triggered current source density (CSD) maps. This could be done with the silicon probe recordings and have the additional advantage that will allow to compare if the CSD profile during head-fixed recordings has differences to that of the delta waves during natural sleep.

• A recent paper (Valero et al., Nat Neurosci, 2021) reported a subset of interneurons (neurogliaform cells) in deep cortical layers that were specifically active during SWS DOWN states and shared other similitudes with the pattern of activation of Sst-Chodl cells. These results seem relevant to the present story.

• In the description of the state-scoring for head-fixed recordings it would be appropriate to acknowledge that, despite their similitudes, these REM and NREM episodes are not durign natural sleep and may therefore have meaningful differences with the equivalent states in freely sleeping animals.

Antonio Fernandez-Ruiz

(Remarks on code availability)

Referee #2

(Remarks to the Author)
Ratliff et al. investigated the role of cortical long-range projecting SST-positive and Chodl-positive GABAergic cells in awake and sleep states. To address this topic, the authors employed various techniques, including molecular genetic tools, optogenetic and chemogenetic manipulation, calcium imaging, electrophysiological recordings, and behavioral state analyses. The authors demonstrated that a subset of SST cells (SST+, Chodl+) projects to other brain areas, SST-Chodl cells are active during slow-wave sleep, activation of SST-Chodl cells increases delta band in cortical rhythms and induce sleep states.
While most cortical inhibitory cells make synapses locally, a small fraction of neocortical and hippocampal GABAergic cells project to other brain areas. Multiple groups have reported that SST-Chodl or SST-Nos cells are among the cortical projecting GABAergic cells. These projecting cortical GABAergic cells have received much attention due to their unique projection patterns that can potentially influence global cortical activity. Indeed, several studies have provided evidence that one of the roles of this group may be related to sleep state. Using various methods, the current study demonstrates that this long-range projecting subset of SST cells can increase cortical synchrony in the low-frequency band and induce sleep state. While the current research elegantly demonstrates how SST-Chodl cells control cortical states and sleep behavior, it builds on prior findings that SST-Chodl GABAergic cells project to other brain areas and are associated with slow-wave sleep. This

study advances our understanding of projecting GABAergic cells by providing not only correlational but also causal evidence, demonstrating that manipulating these cells affects both cortical and behavioral states. Despite the study's convincing results, there is a concern that this study could be perceived as somewhat lacking in novelty.
The results presented in this manuscript are clear and convincing, and the manuscript is, for the most part, well-written. However, there are a few major and several minor issues. Please see my detailed comments below.
Major comments
1. The current study demonstrates two unique properties of SST-Chodl GABAergic cells: first, these neurons project to other brain areas despite being GABAergic, and second, their activity can influence cortical synchrony in the delta band range and slow-wave sleep (SWS) states, even though this group constitutes less than 1% of cortical GABAergic cells. However, it is unclear how these two features are interconnected. My understanding is that the long-range axonal projections of these neurons allow them to regulate broad cortical areas despite their small number. However, this study does not establish a direct link between their projection to other brain areas and the control of widespread cortical activity. Connecting these two properties is crucial for understanding the role of the long-range axonal projections. Alternatively, it is possible that delta band synchrony and SWS states are not induced by long-range SST-Chodl cell innervation but rather by local arborization and synapses from these cells. To address this, I suggest investigating how the activation of SST-Chodl cells in one cortical area affects delta band power in other cortical areas.
2. The authors performed slice electrophysiology to map how SST-Chodl cells locally target other cells within V1 across cortical layers. Related to Major Comment 1, understanding how SST-Chodl cells target neurons in other cortical areas is crucial for elucidating their role in intercortical interactions.
3. The authors reported that SST-Chodl cells project to other cortical areas in a non-uniform manner, with uneven innervation patterns; for example, there is dense innervation in the RL area (Figure 1f, g). However, the injection sites in V1 are spatially biased, predominantly located in the anterior part of V1. To determine whether this uneven innervation is due to injection bias or reflects the inherent projection pattern of SST-Chodl cells, it would be useful to label SST-Chodl cells in different parts of V1 (posterior or lateral) and analyze their axonal innervation in other cortical areas.
4. Figure 1: The data in Figure 1 and from other studies demonstrated that SST-Chodl cells innervate other cortical regions. In current study, the analysis of SST-Chodl cell axonal innervation is performed at the population level. However, it is difficult to understand the extent to which individual SST-Chodl cells project to other cortical areas. How many SST-Chodl cells were labeled at each injection site? How does a single SST-Chodl cell innervate other brain regions?
5. Supplemental Figure 4: It is interesting to note that L6 pyramidal neurons receive strong inhibition from SST-Chodl cells. Please provide the distribution of ChR-expressing SST-Chodl cells to ensure that this finding is not due to injection bias.
6. Figure 2: Please provide the imaging depth for the 97 SST-Chodl cells that were imaged. Is there any relationship between the recording depth and the $\Delta f/F$ of each SST-Chodl cell?
7. Figure 3: The optogenetic stimulation of SST-Chodl cells induces cortical synchrony. Is this function unique to SST-Chodl cells or other SST-positive, Chodl-negative cells share a similar function. Please provide evidence to demonstrate that increasing cortical synchrony is a specific feature of SST-Chodl cells.
8. Figure 3, Supplemental figure 7: The slice electrophysiology results demonstrate that inhibition from SST-Chodl cells is layer-dependent, with stronger inhibition observed in layer 6 pyramidal cells. Given this finding, please provide the analysis of the spike-LFP coherence based on cortical layers (Figure 3) and the firing rate changes based on layers (Supplemental figure 7).
9. Figure 3, Supplemental Figure 7: The duration and amplitude of down-states were increased during optogenetic stimulation, yet the event rate remained unchanged. If the duration of down-states is increased, the event rate should typically decrease. Please clarify this discrepancy.
10. Figures 3, 4: Optogenetic stimulation increased delta power during slow-wave sleep (SWS) and quiet wakefulness (QW), while chemogenetic stimulation increased delta power during SWS and wakefulness. It appears that chemogenetic stimulation has a stronger effect on delta power during wakefulness compared to optogenetic stimulation. Please provide a separate analysis of delta power changes during QW and movement under chemogenetic stimulation.
11. Supplemental Figure 9: It appears that the calcium activity of individual SST-Chodl cells was recorded using 2-photon (2PT) calcium imaging under head-fixed conditions. Please specify the imaging conditions clearly. Since panel ‘a’ shows data from a freely moving home cage condition, this could be confusing. If the data is with 2PT imaging under head-fixed conditions, please provide a population activity analysis of SST-Chodl cells following CNO injection.
Minor comments
Figure 1: Please provide the distribution of SST-Chodl cells across cortical layers in V1.
Figure 3: It appears that this set of experiments was conducted under head-fixed conditions. Please clearly state the experimental conditions used.
Figure 3dleft: please label x-axis.
Figure 3g: It seems that the increased spike-LFP coherence extends beyond the delta band range. Please provide statistical analysis on that.
Figure 4: Please indicate that the LFP recordings are from V1.
Supplemental figure 9f: it appears that SWS data is missing.

(Remarks on code availability)

Referee #3

(Remarks to the Author)
This is an interesting and potentially important manuscript reporting on the function of a rare neocortical cell type, GABAergic Sst/Chodl/NOS1 cells, which send long range projections throughout the cortex. For some years, researchers

have been speculating on the function of these rather exotic cells, and based on the work of one of the co-authors (TS Kilduff) there was a strong anticipation that this particular cell type would be involved in generating sleep. But these cells have been difficult to study because of their sparseness and the requirement for intersectional genetics (to set them apart from all the other sst cell types). Ratliff, Terral et al report on an impressive technical demonstration that the cells can be genetically targeted and functionally investigated. The overall findings reported are interesting, but the key observations presented in Figure 4, that these cells promote NREM sleep, however, seem quite subtle. The data are not very persuasive that these cells are major players in generating sleep. Alternatively, perhaps there is just too much variation in the current data set, or that there are subtypes of Sst/Chodl/NOS1 that do different things. These issues should be resolved in any revised manuscript.

Specific points:

1. Numbers and variability. No power calculations were done. However, in general, some of the n numbers are particularly low, but at the same time, the experimental setting is complex. For example, for the experiments with the tamoxifen-independent conditional Cre recombination mice, using just 3 or 4 mice for in vivo experiments seems low. Although the overall statistics appear significant in key points, the individual plots, before and after, consistently show high variability throughout the optogenetics and chemogenetic experiments.
2. Chronic optogenetics with Ca2+-permeable CatCh by delivering LED stimulation above a thinned skull is an interesting approach; however, this was done in only 3 animals in one brain area. Furthermore, at least, in the example trace in Suppl Fig8b, the delta power is already looking like its increasing even before stimulation. Giving 1s 25mW LED pulses at 0.5Hz for over 1 hour would likely heat up the area generally. Therefore, increasing the cohort size, as well as using negative control experiments in different parts of the cortex would be necessary.
3. For the “whole cortex excitatory DREADD experiment”, the authors injected AAV in multiple sites of cortex to cover wide areas. A technically impressive thing to do. The results in Fig. 4, however, are not compelling that these cells are a major driver of sleep (even though I appreciate that the overall stats show significance). Following CNO, the total duration of sleep is extended by only about 10 mins (Fig. 4c and d), and the time course of this accumulation is slow. These are hardly large effects. While the example trace given in Fig 4b seems to be a striking effect on sleep behaviour induction after CNO injection, including sleep onset shift by around 20mins, the individual plots in Fig4e shows only half the case of rough 20min changes. In fact, individual before-after plots in Fig 4d-h appear the effect is widely variable. This variability is also clear in Suppl Fig 9d.
5. In Fig. 4d, SWS plot, it looks like maybe eight instances of total time in SWS are decreasing following CNO administration. Similarly, in the wake graph, there are 7 or 8 instances of wake increasing. This might suggest that there are two subtypes of Sst-Chodl cell? One promoting wake, the other SWS? The authors should try to resolve this.
6. In Fig. 4c (cumulative sleep time), in addition to the saline control, it would be good to see the effect of CNO on sleep into the Cre/Flp mice that are not expressing the CNO-DREADD receptors.
7. The authors state that no differences in sleep behaviour were detected between the time of the day used for the DREADD experiments (line 746-7). However, they selected injection timing only at ZT3, 5, and 7. While the animals are likely sleepy at these time points, what happens if Sst-Chodl cells are selectively activated in the (lights-off) wakeful time period of the mice?
8. Another possibility: Perhaps the Sst-Chodl cells are following NREM sleep rather than being primary generators? In Fig. 2d, the calcium trace in SWS has a profile which slowly increases during SWS; this does not really look like the profile of a “driver cell”. In this regard, it is interesting to speculate what activates these cells? Presumably glutamate? Is it possible they might receive excitatory inputs from thalamic relay cells, which are active during SWS, which then drive Sst-Chodl to further synchronize cortical activity? In that sense, the SWS-active Sst-Chodl cells would be acting to reinforce neocortical synchrony, but not be primary drivers. Could this be examined by looking at inputs to the Sst-Chodl cells e.g. with rabies-type methods?

Minor points:

9. Please consider adding NOS1 somewhere into the name of these cells, so instead of calling them “Sst-Chodl”, they might, for example, be called “Sst-NOS1-Chodl” instead. This makes the name easier to relate to the large body of previous work by Kilduff et al on NOS1 cells in cortex and sleep.
10. Line 311. Sst-expressing cells and sleep prep behaviour (ref 60). The authors write: “It remains to be seen whether those Sst cells correspond to the Sst-Chodl cells”. It seems likely that they do not. The fos-trapped sampled prefrontal cortex cells in that paper (ref 60) were reported as all Sst positive, all Chodl negative and only a few as Nos1-positive (and also had the capacity to fire at a relatively fast rate); whereas, as a positive control, chodl expression was found in randomly sampled Sst cells.
11. Last part of the Intro is misleading. For the last parts of the Introduction relevant to sleep, I would suggest to rewrite to better reflect current findings, specifically mentioning in the Intro previous work on sst cells, layer V pyramidal cells, REM sleep generation by the cortex, and setting the scene with the historical context, otherwise one gets a bit of a false impression that nothing else has been done in this area (sleep generation in the cortex).
12. The following paper should probably be discussed somewhere. Valero M et al 2021, Sleep-down state-active ID2/Nkx2.1 interneurons in the neocortex. Nat Neurosci 24 (Nos-1-positive, sst-negative cells).

(Remarks on code availability)

Version 2:

Reviewer comments:

Referee #1

(Remarks to the Author)
This is a revision of an original manuscript by Ratliff, Terral and colleagues. This work main focus is the demonstration of a previously unknown role of a sparse and understudied subclass of cortical interneurons, Sst-Chodl cells, in promoting widespread cortical synchronization during non-REM sleep. The paper showcases and impressive combination of complementary experimental and analytical approaches to characterize the properties of Sst-Chold cells in behaving mice and functional role during sleep. The main finding of the paper is that Sst-Chodl cells exert widespread inhibitory modulation of cortical networks to promote synchronization and induce sleep. These exciting findings point to and important and under appreciated role of cortico-cortical inhibition in sleep regulation.

The authors have performed and outstanding job with this revision. In my original assessment I raised several issues that have been fully addressed in the revised manuscript. The authors performed several new experiments and included extensive new data in this revision. One of the new experiments is particularly remarkable as they recorded electrophisiological activity with a laminar flexible probe in the same window were 2-photon imaging was performed. This dual recording enabled new analysis of the temporal relationship between Sst-Chodl cells and cortical sleep dynamics. The analysis of this new dataset clearly demonstrates that Sst-Chodl cell activity reliably tracks NREM sleep episodes and Up-Down fluctuations within them. Other new experiments and analysis include the analysis of the activity of different cortical cell types in a large public dataset, a better characterization of the effects of optogenetic stimulation on Sst-Chodl cell activity and comparison with the effects of stimulating a different subpopulation of Sst interneurons.

Overall the new experimental and analytical evidence provided with this revision reinforced and expanded the previous results and provided strong support for the authors conclusions. I do not have more issues to raise and believe this paper will be an important contribution to the field.

(Remarks on code availability)
NA

Referee #2

(Remarks to the Author)
The authors have added significant new data that solidify the main finding that Sst-Chodl GABAergic neurons are long-range projecting and that their manipulation causally promotes sleep-related cortical synchrony and behavioral state changes.
In terms of novelty of the current study, previous studies have largely established correlations between Sst-Chodl neurons and sleep-related cortical states. The present work provides a causal link, offering more direct evidence that manipulating this sparse subtype can influence low-frequency synchrony and behavioral state. A study published in Neuron last year from Khodosevich’s group reported similar findings, demonstrating sleep disturbances and rescue via chemogenetic manipulation of Sst-Chodl neurons (PMID: 40997796), which is conceptually aligned with the current study.

Specific Comments

1. Single-cell reconstructions (Figure 1b–e)
The manuscript analyzes axon path length and projection density, but axon presence does not necessarily imply synaptic arborization. Are the quantified measures reflecting true terminal arborization or simply axonal trajectories? Given that Sst-Chodl axons travel via superficial layers, how were fibers of passage distinguished from arborization? Clear definitions of these metrics would strengthen interpretation.

2. Contribution of long-range projections to delta synchrony (Figure 4)
The new multisite LFP recordings show that optogenetic stimulation in V1 increases low-frequency power at distant cortical sites, with strongest effects near the stimulation area. However, alternative interpretations remain possible. Light spread could contribute to stronger proximal effects. Alternatively, distal delta power changes could arise indirectly through polysynaptic corticocortical propagation rather than direct long-range inhibitory terminals. A subset of V1 neurons increases firing during stimulation, which may facilitate indirect network spread. More direct evidence would be stimulation of Sst-Chodl terminals in distal cortical regions where they innervate, or analyses that more definitively separate direct long-range synaptic effects from secondary network propagation.

(Remarks on code availability)

Referee #3

(Remarks to the Author)
I think the authors have responded very well to all my points with extensive new data and revisions. The work is done to an impressive technical standard. The data are now much more convincing (e.g. Fig. 5, sleep induction in whole cortex DREADD experiments). In summary, this is an important study characterizing how a special subset of somatostatin-chodl cells coordinates cortical activity and can prompt wake to sleep transitions. The work will interest both sleep physiologists and those working on cortical network function.

Happy to sign as: William Wisden

(Remarks on code availability)

Open Access This Peer Review File is licensed under a Creative Commons Attribution 4.0 International License, which permits use, sharing, adaptation, distribution and reproduction in any medium or format, as long as you give appropriate credit to the original author(s) and the source, provide a link to the Creative Commons license, and indicate if changes were made.
In cases where reviewers are anonymous, credit should be given to 'Anonymous Referee' and the source.
The images or other third party material in this Peer Review File are included in the article's Creative Commons license, unless indicated otherwise in a credit line to the material. If material is not included in the article's Creative Commons license and your intended use is not permitted by statutory regulation or exceeds the permitted use, you will need to obtain permission directly from the copyright holder.
To view a copy of this license, visit https://creativecommons.org/licenses/by/4.0/

Point-by-point response to the referees

We thank the reviewers for the thoughtful and constructive criticism and suggestions. Addressing the mentioned issues will strengthen the paper and we are very much looking forward to do so. Below, **in blue**, we outline point by point how we plan to address reviewers suggestions.

Referee #1 (Remarks to the Author):

Current dominant theories of sleep propose that it is regulated by the activity of subcortical nuclei with widespread connections with the neocortex and the rest of the brain. However, recent evidence suggest that the neocortex can also play a leading role in sleep state regulation. This work follows that direction to tackle an important and unsolved question, whether there are specialized cortical cell types that regulate the level of cortical network synchrony and promote sleep. A hallmark of non-REM (NREM) sleep are the highly synchronized cortical dynamics that fluctuate at ~1Hz between states of high (UP) and low (DONW) activity. This manuscript reports a previously unknow cellular mechanism that promotes widespread cortical synchronization. The authors identified a specific subset of interneurons in the mouse primary visual cortex (V1), within the broad family of somatostatin expressing cells, termed Sst-Chodl cells, with dense local and long-range cortical projections. Sst-Chodl cells were preferentially active during periods of low arousal and cortical synchronization, unlike many other interneuron subtypes. Remarkably opto- and chemogenetic activation of Sst-Chodl cells induced cortical synchronization and promoted sleep (by reducing the latency and increased the duration of NREM episodes). The manuscript features an impressive combination of different experimental approaches to thoroughly characterize the properties of Sst-Chodl cells at the morphological, physiological and behavioral levels. Experimental and analytical methods are sound, and the results presented support the author's interpretation on the causal role Sst-Chodl cells in promoting high cortical synchrony. Overall, this is a rigorous and comprehensive study that shines new light into a poorly understood aspect of sleep, the contribution of local cortical mechanisms, and pinpoints a sparse and highly homogenous cell type as a novel key element in this process.

• One of the main hypotheses of the paper is that Sst-Chodl cells play a key role in promoting NREM sleep (or Slow Wave Sleep). The data provided (for example in Fig. 2) clearly supports that Sst-Chodl cells are preferentially active during low arousal states. Fig. 2d suggests that the increase in activity of Sst-Chodl cells is delayed respect the onset of SWS. How do the authors interpret this observation in relation to their proposed functional role? Could Sst-Chodl cells role be to promote the initiation of synchronized states (i.e. transition from REM to SWS) or rather the maintenance of SWS state? A more sensitive analysis to further explore this question would be to calculate the latency to activation from SWS onset for each Sst-Chodl cell. Another could be to use a time-resolved GLM, or other similar approach, to test the predictive power of Sst-Chodl activity on different state transitions (i.e., does the change in Sst-Chodl population activity predict the onset or offset of SWS episodes?). Likewise, it would be possible to test

whether Sst-Chodl activity is correlated with the maintenance of synchronized states in a similar manner. For example, is the level of Sst-Chodl activity correlated with/ is predictive of the duration of SWS bouts? Similar analysis for stillness vs movement could also be useful (and some were already provided) but in that case the interpretation is more complicated due to other contributing factors.

We plan to specifically test the hypothesis of Sst-Chodl cells role in promoting vs maintaining synchronized states using several methods suggested by the reviewer including:

- Using time resolved GLM methods suggested by the reviewer to examine how the temporal activation patterns of Sst-Chodl cells are predictive of state transitions, with particular interest in QW activity preceding a NREM transition.
- Testing hypotheses of maintenance of SWS by Sst-Chodl cell activity by regressing Sst-Chodl cell activity by time remaining in SWS bouts

• An analysis that would strengthen the uniqueness of the activation pattern of Sst-Chodl cells is to calculate their correlation with other cell types. This can be done in the Allen dataset by calculating cross-correlograms of putative Sst-Chodl cells against the rest of the population. This may show a strong anti-correlation of activity, while doing the same analysis with other Sst cells (or other cell types) will show a positive correlation.

We plan to take two routes to address the uniqueness of the activation patterns of Sst-Chodl cells:

- Calculate the cross-correlation between putative Sst-Chodl cells and other Sst cells in the Allen datasets, as suggested by the reviewer.
- Perform dual imaging experiments with GCaMP in all Sst cells and a red fluorophore in Sst-Chodl cells.

• Were Sst-Chodl cells identified in the silicon probe recording experiments (through optogenetic-tagging)? This would offer the possibility to provide important additional characterization of these cells, including their firing, burstiness and waveform properties. The analysis mentioned in the previous point will be even more convincing using opto-tagged Sst-Chodl cells.

Amongst ~450 recorded cells no optotagged units were found highlighting the difficulty of locating this scarce population in silicon probe recordings. Regardless, we plan to pursue several avenues towards this fruitful analysis:

- Examine additional collected data (not yet included in this manuscript) to look for tagged units.
- Mine the Allen Institute optotagging datasets (with approximately 600 tagged Sst cells) to look for cells with Sst-Chodl like activation patterns.

- Attempt additional optotagging experiments with higher recording site density probes in collaboration with the Allen Institute.

• SWS is not a homogenous state but rather consists in the alternation between periods of high (UP) and low (DOWN) activity. The authors briefly mention this in Fig. 3 but surprisingly did not perform any analysis of Sst-Chodl cells activity in relation to this fundamental feature of SWS. Such analyses are needed to better understand the role this cell type. Since delta waves (reflecting UP-DOWN transitions) were detected together with 2-photon imaging, it would be possible to analyze Sst-Chodl activity in relation to this two substates: are Sst-Chodl active durign DOWN states contrary to most other cortical cells?, what is the timing of activation of Sst-Chodl cells respect to the DOWN to UP / UP to DOWN transition?, does the activity of Sst-Chodl predict the onset of duration of a subsequent DOWN state?

We plan to perform additional analyses to determine the relationship between Sst-Chodl cells and DOWN states as suggested by the reviewer:

- Image additional Sst-Chodl cells increasing the temporal resolution using line scans and then analyzing the activity of Sst-Chodl cells relative to the peak of DOWN states and the transitions between UP and DOWN states.
- We will correlate preceding Sst-Chodl cell activity with the strength of DOWN states (e.g. duration, amplitude).
- Examining DOWN state occurrence relative to phase of sinusoidal stimulation of Sst-Chodl cells

• To better understand the network effect of optogenetic stimulation of Sst-Chodl cells the authors should perform peri-stimulation analysis rather than just analyze the changes between stimulation and non-stimulation blocks. That this, they can plot LFP power changes across the depth profile triggered on stimulation pulses, peri-pulse firing histograms (PSTH), etc. If discrete (e.g., single square pulses) pulses were not delivered, similar analysis could be performed with the phase of sinusoidal stimulation pulses. Some of these analyses can also be done with the freely moving EEG/ LFP recordings, which are currently only minimally analyzed.

We will perform the peri-stimulus analyses suggested by the reviewer examining LFP power changes, spiking activity, and DOWN states. We will utilize both short pulses and analyze relative to sinusoidal phase.

• Optogenetic stimulation of Sst-Chodl cells increases the spike coupling to delta waves (Fig. 3g-h). However, the current analyses do not allow to evaluate whether similar or opposite changes happen with other frequency bands. This may be particularly relevant for the high gamma-frequency band (~90-150 Hz) characteristic of deep cortical layers, the same that show the strongest changes in delta power. Both gamma and delta power are associated with UP-DOWN states. Did the stimulation affected the spiking structure of these states (e.g., unit firing timing within UP states, cell participation and magnitude of activation withing UP states, suppression of firing during DOWN states, etc.).

We plan to perform the following additional analyses to address this aspect of the data:

- Extend the analysis of spike-LFP coherence to high gamma band
- Perform statistical analysis of spike-LFP coherence in other frequency band (theta, gamma, high gamma, etc)
- Analyze the magnitude of delta band and high gamma band coupling in relationship to layer location.
- Include additional analysis of spiking structure during Sst-Chodl cell stimulation:
    a. Unit participation rate in UP state transients
    b. Magnitude of firing rate change around DOWN to UP transitions
    c. Sequence of activation around DOWN to UP transitions
    d. Magnitude of suppression of firing rate during DOWN states
    e. ISI and "burstiness" of cells during stimulation

• The authors report minimal changes in firing rates due to optogenetic stimulation of Sst-Chodl, but were the spiking dynamics on individual cells affected? This can be evaluated by computing the coefficient of variation of single unit spike trains, the propensity of bursting, etc. Was the excitatory/ inhibitory balance affected?

We plan include several analyses to address the reviewer's comments around single cell spiking dynamics in relationship to Sst-Chodl stimulation:

- Analyzing auto-correlograms of single units
- Calculating the "burstiness" of units
- Examining the distribution of inter-spike intervals
- Examining the E-I balance

• An accurate way of determined cortical layers is by calculating delta wave-triggered current source density (CSD) maps. This could be done with the silicon probe recordings and have the additional advantage that will allow to compare if the CSD profile during head-fixed recordings has differences to that of the delta waves during natural sleep.

We will compare the CSD profile of DOWN-UP transitions with respect to Sst-Chodl cell stimulation.

• A recent paper (Valero et al., Nat Neurosci, 2021) reported a subset of interneurons (neurogliaform cells) in deep cortical layers that were specifically active during SWS DOWN states and shared other similitudes with the pattern of activation of Sst-Chodl cells. These results seem relevant to the present story.

We apologize for the oversite in missing this very relevant manuscript that will now be included.

• In the description of the state-scoring for head-fixed recordings it would be appropriate to acknowledge that, despite their similitudes, these REM and NREM episodes are not durign natural sleep and may therefore have meaningful differences with the equivalent states in freely

sleeping animals.

We will clarify in the text stating that “REM and NREM episodes are not during natural sleep and may therefore have meaningful differences with the equivalent states in freely sleeping animals“ and include supplementary figure comparing head-fixed and non-head fixed sleep scoring in the same animal.

Referee #2 (Remarks to the Author):

Ratliff et al. investigated the role of cortical long-range projecting SST-positive and Chodl-positive GABAergic cells in awake and sleep states. To address this topic, the authors employed various techniques, including molecular genetic tools, optogenetic and chemogenetic manipulation, calcium imaging, electrophysiological recordings, and behavioral state analyses. The authors demonstrated that a subset of SST cells (SST+, Chodl+) projects to other brain areas, SST-Chodl cells are active during slow-wave sleep, activation of SST-Chodl cells increases delta band in cortical rhythms and induce sleep states.
While most cortical inhibitory cells make synapses locally, a small fraction of neocortical and hippocampal GABAergic cells project to other brain areas. Multiple groups have reported that SST-Chodl or SST-Nos cells are among the cortical projecting GABAergic cells. These projecting cortical GABAergic cells have received much attention due to their unique projection patterns that can potentially influence global cortical activity. Indeed, several studies have provided evidence that one of the roles of this group may be related to sleep state. Using various methods, the current study demonstrates that this long-range projecting subset of SST cells can increase cortical synchrony in the low-frequency band and induce sleep state. While the current research elegantly demonstrates how SST-Chodl cells control cortical states and sleep behavior, it builds on prior findings that SST-Chodl GABAergic cells project to other brain areas and are associated with slow-wave sleep. This study advances our understanding of projecting GABAergic cells by providing not only correlational but also causal evidence, demonstrating that manipulating these cells affects both cortical and behavioral states. Despite the study's convincing results, there is a concern that this study could be perceived as somewhat lacking in novelty.
The results presented in this manuscript are clear and convincing, and the manuscript is, for the most part, well-written. However, there are a few major and several minor issues. Please see my detailed comments below.

We thank the reviewer for these thoughtful comments. While this is the first work that specifically targeted Sst/Chodl cells and analyzed their *in vivo* function, some of the reported results may have been anticipated based on previous research. In the revised version we will clarify what is novel about this work and will include new experiments that will strengthen the novelty of this work. In particular we will clarify that:

- While somewhat unemphasized in the previous version of this manuscript, Sst-Chodl cell stimulation has profound impacts on cortical synchrony causing changes similar in magnitude to those seen during wake to sleep transitions.
- These cells have never been imaged *in vivo* previously, and while imaging results are in agreement with previous cFos results, our results expand substantially on the predicted activation during sleep.
- There have been no direct, targeted, clean manipulations of Sst-Chodl cells before this work (previous work includes whole brain nNOS KO, manipulation of larger Sst or nNOS cell populations of which Sst-Chodl is a minority)
- No work has previously linked the activity of such a small and distinct population of cells in the cortex to sleep. The experiments here challenge a dogma in the field of subcortical control of sleep.

In addition to the mentioned clarifications we plan to include several in progress new experiments to strengthen the novelty of this work and extend it further beyond what might have been previously hypothesized. In particular, we will investigate what are Sst-Chodl cells inputs, and we will be completing experiments to examine the role of the long-range projections of Sst-Chodl cells and to perform causal experiments relating these cells to sleep homeostasis.

Major comments

1. The current study demonstrates two unique properties of SST-Chodl GABAergic cells: first, these neurons project to other brain areas despite being GABAergic, and second, their activity can influence cortical synchrony in the delta band range and slow-wave sleep (SWS) states, even though this group constitutes less than 1% of cortical GABAergic cells. However, it is unclear how these two features are interconnected. My understanding is that the long-range axonal projections of these neurons allow them to regulate broad cortical areas despite their small number. However, this study does not establish a direct link between their projection to other brain areas and the control of widespread cortical activity. Connecting these two properties is crucial for understanding the role of the long-range axonal projections. Alternatively, it is possible that delta band synchrony and SWS states are not induced by long-range SST-Chodl cell innervation but rather by local arborization and synapses from these cells. To address this, I suggest investigating how the activation of SST-Chodl cells in one cortical area affects delta band power in other cortical areas.

We thank the reviewer for this constructive comment. We will be adding two complementary experiments to address this:

- We will perform slice electrophysiology experiments to determine the impact of long-range Sst-Chodl axons on cells recorded far away from Sst-Chodl cell bodies.
- We will perform additional silicon probe recordings with multishank probes that span more than 1mm of cortical space. Stimulation will be directed to the first shanks and we will analyze, across shanks, the spatial features of Sst-Chodl cell induced synchronization.

2. The authors performed slice electrophysiology to map how SST-Chodl cells locally target other cells within V1 across cortical layers. Related to Major Comment 1, understanding how SST-Chodl cells target neurons in other cortical areas is crucial for elucidating their role in intercortical interactions.

See above comment where the same issue is addressed

3. The authors reported that SST-Chodl cells project to other cortical areas in a non-uniform manner, with uneven innervation patterns; for example, there is dense innervation in the RL area (Figure 1f, g). However, the injection sites in V1 are spatially biased, predominantly located in the anterior part of V1. To determine whether this uneven innervation is due to injection bias or reflects the inherent projection pattern of SST-Chodl cells, it would be useful to label SST-Chodl cells in different parts of V1 (posterior or lateral) and analyze their axonal innervation in other cortical areas.

We will characterize the differences between projection patterns of Sst-Chodl cells labelled with anterior, posterior, and lateral injections in V1.

4. Figure 1: The data in Figure 1 and from other studies demonstrated that SST-Chodl cells innervate other cortical regions. In current study, the analysis of SST-Chodl cell axonal innervation is performed at the population level. However, it is difficult to understand the extent to which individual SST-Chodl cells project to other cortical areas. How many SST-Chodl cells were labeled at each injection site? How does a single SST-Chodl cell innervate other brain regions?

We will characterize the single cell projections patterns of Sst-Chodl cells in whole brain 3D reconstructions.

We will also clarify the number of Sst-Chodl cells labeled in each brain and relate this to subsequent arborization.

5. Supplemental Figure 4: It is interesting to note that L6 pyramidal neurons receive strong inhibition from SST-Chodl cells. Please provide the distribution of ChR-expressing SST-Chodl cells to ensure that this finding is not due to injection bias.

We will add additional characterization of the distribution of the ChR2-eYFP labelled Sst-Chodl axonal arbor in our recordings.

6. Figure 2: Please provide the imaging depth for the 97 SST-Chodl cells that were imaged. Is there any relationship between the recording depth and the $\Delta f/F$ of each SST-Chodl cell?

To address the relationship between layer depth and function Sst-Chodl cells, we will:

- Analyze and/or perform additional experiments to determine the relationship between the signal activity and imaging depth.
- Compare intrinsic properties of Sst-Chodl cells across layers

7. Figure 3: The optogenetic stimulation of SST-Chodl cells induces cortical synchrony. Is this function unique to SST-Chodl cells or other SST-positive, Chodl-negative cells share a similar function. Please provide evidence to demonstrate that increasing cortical synchrony is a specific feature of SST-Chodl cells.

To address the uniqueness of Sst-Chodl cells in generating low arousal synchrony, we will perform optogenetic manipulation of Martinotti cells (a specific class of Chodl-negative Sst cells) using recently published enhancer viruses and compare their activity to Sst-Chodl cells.

8. Figure 3, Supplemental figure 7: The slice electrophysiology results demonstrate that inhibition from SST-Chodl cells is layer-dependent, with stronger inhibition observed in layer 6 pyramidal cells. Given this finding, please provide the analysis of the spike-LFP coherence based on cortical layers (Figure 3) and the firing rate changes based on layers (Supplemental figure 7).

We will divide spike-LFP coherence analysis by layer and by frequency as well as dividing firing rate analysis by layer.

9. Figure 3, Supplemental Figure 7: The duration and amplitude of down-states were increased during optogenetic stimulation, yet the event rate remained unchanged. If the duration of down-states is increased, the event rate should typically decrease. Please clarify this discrepancy.

We will clarify in the text why rate and duration are able to change independently and change below the detection level of statistical significance.

10. Figures 3, 4: Optogenetic stimulation increased delta power during slow-wave sleep (SWS) and quiet wakefulness (QW), while chemogenetic stimulation increased delta power during SWS and wakefulness. It appears that chemogenetic stimulation has a stronger effect on delta power during wakefulness compared to optogenetic stimulation. Please provide a separate analysis of delta power changes during QW and movement under chemogenetic stimulation.

We will provide analysis of synchrony in optogenetic and chemogenetic manipulation across states and clarify differences in effect size between the two conditions keeping in mind that many additional cells are activated by chemogenetic manipulation.

11. Supplemental Figure 9: It appears that the calcium activity of individual SST-Chodl cells was recorded using 2-photon (2PT) calcium imaging under head-fixed conditions. Please specify the imaging conditions clearly. Since panel ‘a’ shows data from a freely moving home cage condition, this could be confusing. If the data is with 2PT imaging under head-fixed conditions, please provide a population activity analysis of SST-Chodl cells following CNO injection.

We will include a schematic for this figure to indicate head fixation and also add a population analysis of effects of CNO activation.

Minor comments
Figure 1: Please provide the distribution of SST-Chodl cells across cortical layers in V1.

We will provide this information.

Figure 3: It appears that this set of experiments was conducted under head-fixed conditions. Please clearly state the experimental conditions used.

We will add a clear schematic and text clarifying the experimental conditions.

Figure 3dleft: please label x-axis.

We will add this

Figure 3g: It seems that the increased spike-LFP coherence extends beyond the delta band range. Please provide statistical analysis on that.

We will provide this analysis (please see above)

Figure 4: Please indicate that the LFP recordings are from V1.

Recordings are from V1 and we will make this clarification.

Supplemental figure 9f: it appears that SWS data is missing.

We apologize for the lack of clarity in the graph. Because of the absence of variability, the representation seems to not show data. We will provide another representation of the data.

Referee #3 (Remarks to the Author):

This is an interesting and potentially important manuscript reporting on the function of a rare neocortical cell type, GABAergic Sst/Chodl/NOS1 cells, which send long range projections throughout the cortex. For some years, researchers have been speculating on the function of these rather exotic cells, and based on the work of one of the co-authors (TS Kilduff) there was a strong anticipation that this particular cell type would be involved in generating sleep. But these cells have been difficult to study because of their sparseness and the requirement for intersectional genetics (to set them apart from all the other sst cell types). Ratliff, Terral et al report on an impressive technical demonstration that the cells can be genetically targeted and functionally investigated. The overall findings reported are interesting, but the key observations

presented in Figure 4, that these cells promote NREM sleep, however, seem quite subtle. The data are not very persuasive that these cells are major players in generating sleep. Alternatively, perhaps there is just too much variation in the current data set, or that there are subtypes of Sst/Chodl/NOS1 that do different things. These issues should be resolved in any revised manuscript.

We thank the reviewer for their comments. We will add several additional pieces of data as well as clarifications as to the effect. We would like to point out several experiment factors that might be responsible of the variability and subtle effect in our dataset:

- We use multisite injections to target specifically the neocortex, while avoiding other brain regions. While our coordinates were chosen because of the maximal coverage of the neocortex, it is likely that not all cortical Sst-Chodl neurons were transfected and thus activated by CNO, and the efficiency of targeting varies from animal to animal.
- We use a low dose of CNO to avoid off-targeted effects. While we were aware that such low CNO dose, 2 to 10 times less concentrated CNO than other studies (Varin et al. 2018; Traut et al., 2023), is likely to not be very robust in activating all DREAD expressing Sst-Chodl cells, we decided on such dose to be sure that any observed effects were not due to CNO.
- The sleep-wake cycle of mice is variable between individuals but also between days within a single animal. We will clarify this point by showing population analysis of effects of CNO activation. In addition we will extend the tome window of when we analyze sleep.

Specific points:

1. Numbers and variability. No power calculations were done. However, in general, some of the n numbers are particularly low, but at the same time, the experimental setting is complex. For example, for the experiments with the tamoxifen-independent conditional Cre recombination mice, using just 3 or 4 mice for in vivo experiments seems low. Although the overall statistics appear significant in key points, the individual plots, before and after, consistently show high variability throughout the optogenetics and chemogenetic experiments.

We agree with the reviewer’s comment on sample size and will augment our optogenetics and chemogenetics datasets adding several mice.

2. Chronic optogenetics with Ca2+-permeable CatCh by delivering LED stimulation above a thinned skull is an interesting approach; however, this was done in only 3 animals in one brain area. Furthermore, at least, in the example trace in Suppl Fig8b, the delta power is already looking like its increasing even before stimulation. Giving 1s 25mW LED pulses at 0.5Hz for over 1 hour would likely heat up the area generally. Therefore, increasing the cohort size, as well as using negative control experiments in different parts of the cortex would be necessary.

We performed control experiments (CreER animals which never received tamoxifen) concurrently with the presented animals and did not observe any effect of LEDs on the delta power. We chose to omit this data to be concise. We do not believe this experiment to be

optimal/crucial to study the pan-neocortical delta changes for the reason provided by the reviewer, but rather supports other data presented. We can clarify in the text the potential issues raised by the reviewer and include the control group data or remove it from the manuscript if the reviewer prefers.

3. For the “whole cortex excitatory DREADD experiment”, the authors injected AAV in multiple sites of cortex to cover wide areas. A technically impressive thing to do. The results in Fig. 4, however, are not compelling that these cells are a major driver of sleep (even though I appreciate that the overall stats show significance). Following CNO, the total duration of sleep is extended by only about 10 mins (Fig. 4c and d), and the time course of this accumulation is slow. These are hardly large effects. While the example trace given in Fig 4b seems to be a striking effect on sleep behaviour induction after CNO injection, including sleep onset shift by around 20mins, the individual plots in Fig4e shows only half the case of rough 20min changes. In fact, individual before-after plots in Fig 4d-h appear the effect is widely variable. This variability is also clear in Suppl Fig 9d.

Agreed but it is likely that only a small portion of cortical Sst-Chodl neurons were transfected and thus activated by CNO. Additionally we used the minimal dose of CNO, and while this prevents off-target effects, it likely does not activate Sst-Chodl cells to the same degree that higher concentrations would. We are exploring different avenues to address the reviewer comment including:

- Use another example for 4b more “representative”
- Reformulate our hypothesis with the cortex not as a sleep driver, but as a sleep regulator. To test this hypothesis we will compare unperturbed sleep to recovery sleep.
- Perform new experiments on sleep homeostasis
- Repeat the same experiment at different time that likely gives us stronger effects (to get stronger effect, ZT18)
- Increase length of recording during the sleep experiment to 4-5h
- Clarify our data by showing population analysis of effects of CNO activation
- Perform additional experiments for minimizing the effect variability

5. In Fig. 4d, SWS plot, it looks like maybe eight instances of total time in SWS are decreasing following CNO administration. Similarly, in the wake graph, there are 7 or 8 instances of wake increasing. This might suggest that there are two subtypes of Sst-Chodl cell? One promoting wake, the other SWS? The authors should try to resolve this.

We agree with the reviewer that our data show some variability (see above). To clarify as to whether sessions with decreased sleep are driven by different subtypes of Sst-Chodl cells, we will include new analysis showing the animal average effect of CNO injection. This shows that each animal included here on average spends increased time sleeping.

That being said the reviewer raises an interesting point about potential subtypes of Sst-Chodl cells. Indeed, we have found that a small proportion of cells in our imaging dataset have

opposite pattern of activity, being correlated with high arousal states. To provide further explanation of potential subtypes of Sst-Chodl cells we will perform new experiments to investigate the relationship between Sst-Chodl cell activity and their molecular profile. In particular we will image Sst-Chodl cells across depths using implanted microprisms to determine if different "activity types" exist across layers. In addition, we will combine imaging of Sst-Chodl cells with post-hoc immunohistochemistry to determine cell type identify of imaged cells with particular respect to arousal correlated vs anti-correlated cells.

6. In Fig. 4c (cumulative sleep time), in addition to the saline control, it would be good to see the effect of CNO on sleep into the Cre/Flp mice that are not expressing the CNO-DREADD receptors.

Please find this data presented in Supplemental Figure 9g

7. The authors state that no differences in sleep behavior were detected between the time of the day used for the DREADD experiments (line 746-7). However, they selected injection timing only at ZT3, 5, and 7. While the animals are likely sleepy at these time points, what happens if Sst-Chodl cells are selectively activated in the (lights-off) wakeful time period of the mice?

We will add experiments testing activation of Sst-Chodl cells at different times of day. We hypothesize that the sleep promoting effect may be greater during periods with greater sleep need at later ZTs.

8. Another possibility: Perhaps the Sst-Chodl cells are following NREM sleep rather than being primary generators? In Fig. 2d, the calcium trace in SWS has a profile which slowly increases during SWS; this does not really look like the profile of a "driver cell". In this regard, it is interesting to speculate what activates these cells? Presumably glutamate? Is it possible they might receive excitatory inputs from thalamic relay cells, which are active during SWS, which then drive Sst-Chodl to further synchronize cortical activity? In that sense, the SWS-active Sst-Chodl cells would be acting to reinforce neocortical synchrony, but not be primary drivers. Could this be examined by looking at inputs to the Sst-Chodl cells e.g. with rabies-type methods?

We have performed rabies tracing from these cells showing cortical and thalamic input without input from the thalamic reticular nucleus. We will include this new data. In regards to the idea of "driving" vs "maintaining" sleep, we have detailed several analyses above to characterize this.

Minor points:

9. Please consider adding NOS1 somewhere into the name of these cells, so instead of calling them "Sst-Chodl", they might, for example, be called "Sst-NOS1-Chodl" instead. This makes the name easier to relate to the large body of previous work by Kilduff et al on NOS1 cells in cortex and sleep.

While we acknowledge that this cells also express Nos, we prefer to call them Sst-Chodl in order to keep consistent with previous papers that used this terminology. However, we have revised the text in order to make the clarification requested by the reviewer

10. Line 311. Sst-expressing cells and sleep prep behaviour (ref 60). The authors write: "It remains to be seen whether those Sst cells correspond to the Sst-Chodl cells". It seems likely that they do not. The fos-trapped sampled prefrontal cortex cells in that paper (ref 60) were reported as all Sst positive, all Chodl negative and only a few as Nos1-positive (and also had the capacity to fire at a relatively fast rate); whereas, as a positive control, chodl expression was found in randomly sampled Sst cells.

We will revise.

11. Last part of the Intro is misleading. For the last parts of the Introduction relevant to sleep, I would suggest to rewrite to better reflect current findings, specifically mentioning in the Intro previous work on sst cells, layer V pyramidal cells, REM sleep generation by the cortex, and setting the scene with the historical context, otherwise one gets a bit of a false impression that nothing else has been done in this area (sleep generation in the cortex).
We will revise.

12. The following paper should probably be discussed somewhere. Valero M et al 2021, Sleep-down state-active ID2/Nkx2.1 interneurons in the neocortex. Nat Neurosci 24 (Nos-1-positive, sst-negative cells).
We will add this paper.

Point-by-point response to the referees

We sincerely thank all the reviewers for the thoughtful, constructive, and insightful comments. We found the feedback highly valuable, and addressing the points raised has substantially strengthened the manuscript. In response, we have carried out additional experiments and analyses, expanded several experiments, and revised key sections of the text to improve clarity and rigor.

The revised manuscript includes 1 new main figure, 7 new supplemental figures and multiple updates to existing figures and analyses. We have also refined the narrative throughout to better highlight the conceptual advances and ensure that all methodological details are fully transparent.

Below, we provide a detailed, point-by-point response to each reviewer comment. Reviewer comments are shown in black, and our responses are shown in blue.

In the revised manuscript, substantive changes (new data, analyses, or interpretations, and text modified in direct response to reviewer requests) are highlighted in blue. In this response document, new or modified manuscript text is reproduced in quotation marks to facilitate cross-referencing with the revised manuscript. Minor editorial and stylistic edits made for clarity and consistency are not highlighted.

Referee #1 (Remarks to the Author):

Current dominant theories of sleep propose that it is regulated by the activity of subcortical nuclei with widespread connections with the neocortex and the rest of the brain. However, recent evidence suggest that the neocortex can also play a leading role in sleep state regulation. This work follows that direction to tackle an important and unsolved question, whether there are specialized cortical cell types that regulate the level of cortical network synchrony and promote sleep. A hallmark of non-REM (NREM) sleep are the highly synchronized cortical dynamics that fluctuate at ~1Hz between states of high (UP) and low (DONW) activity. This manuscript reports a previously unknow cellular mechanism that promotes widespread cortical synchronization. The authors identified a specific subset of interneurons in the mouse primary visual cortex (V1), within the broad family of somatostatin expressing cells, termed Sst-Chodl cells, with dense local and long-range cortical projections. Sst-Chodl cells were preferentially active during periods of low arousal and cortical synchronization, unlike many other interneuron subtypes. Remarkably opto- and chemogenetic activation of Sst-Chodl cells induced cortical synchronization and promoted sleep (by reducing the latency and increased the duration of NREM episodes). The manuscript features an impressive combination of different experimental approaches to thoroughly characterize the properties of Sst-Chodl cells at the morphological, physiological and behavioral levels. Experimental and analytical methods are sound, and the results presented support the author's interpretation on the causal role Sst-Chodl cells in promoting high cortical synchrony. Overall, this is a rigorous and comprehensive study that shines new light into a poorly understood aspect of sleep, the contribution of local cortical mechanisms, and pinpoints a sparse and highly homogenous cell type as a novel key element in this process.

• One of the main hypotheses of the paper is that Sst-Chodl cells play a key role in promoting NREM sleep (or Slow Wave Sleep). The data provided (for example in Fig. 2) clearly supports that Sst-Chodl cells are preferentially active during low arousal states. Fig. 2d suggests that the

increase in activity of Sst-Chodl cells is delayed respect the onset of SWS. How do the authors interpret this observation in relation to their proposed functional role? Could Sst-Chodl cells role be to promote the initiation of synchronized states (i.e. transition from REM to SWS) or rather the maintenance of SWS state? A more sensitive analysis to further explore this question would be to calculate the latency to activation from SWS onset for each Sst-Chodl cell. Another could be to use a time-resolved GLM, or other similar approach, to test the predictive power of Sst-Chodl activity on different state transitions (i.e., does the change in Sst-Chodl population activity predict the onset or offset of SWS episodes?). Likewise, it would be possible to test whether Sst-Chodl activity is correlated with the maintenance of synchronized states in a similar manner. For example, is the level of Sst-Chodl activity correlated with/ is predictive of the duration of SWS bouts? Similar analysis for stillness vs movement could also be useful (and some were already provided) but in that case the interpretation is more complicated due to other contributing factors.

We thank the reviewer for these constructive insights into the temporal coupling between Sst-Chodl cell activity and sleep state transitions. To examine this question with greater precision, we performed additional experiments using a flexible 32-channel probe implanted beneath the cranial window used for 2-photon experiments. This approach enabled simultaneous measurement of Sst-Chodl calcium signals, local spiking activity, and LFP dynamics within the same cortical volume. We also increased the temporal resolution of our calcium imaging to better capture sub-second UP/DOWN fluctuations.

To directly address the reviewer's suggestions, we have added new analysis and figure panels (Fig 2 e-k, Supp Fig 7). The following text has been incorporated into the revised manuscript:

"To examine the temporal relationship between Sst-Chodl cell activity and cortical dynamics, we simultaneously recorded Sst-Chodl calcium signals and LFPs from the same cortical region (ipsilateral V1). Flexible 32-channel probes[64] were implanted beneath the imaging window, enabling high-temporal resolution 2-photon imaging of Sst-Chodl cell activity alongside electrophysiological recordings (see Methods; Fig 2e-k). Sst-Chodl cells exhibited state-dependent changes in activity that closely tracked fluctuations in LFP in delta power (Fig 2f and Supp Fig 6l). As mice entered SWS, both Sst-Chodl cell activity and delta power increased and remained elevated throughout sleep bouts. Conversely, transitions into desynchronized states (wake or REM sleep) were accompanied by concurrent decreases in both Sst-Chodl cell activity and delta power (Fig 2f). Sst-Chodl cell activity closely co-varied with delta power rather than anticipating future changes in cortical synchrony. Consistent with this interpretation, a time-resolved generalized linear model showed that Sst-Chodl cell activity did not predict changes in delta power beyond 5 sec time window (Fig 2g; Supp Fig 7a). Cross-correlation analysis further revealed that coupling between calcium activity and delta power peaked at a short positive lag, consistent with near-simultaneous co-variation rather than predictive lead–lag relationships (Fig 2h). In addition, neither the mean activity of Sst-Chodl cells during SWS bouts nor other examined features were correlated with SWS bout duration (Supp Fig 7b-c). Together, these results suggest that Sst-Chodl cell activity reflects ongoing sleep-related cortical dynamics rather than anticipating forthcoming state transitions."

In addition, as the reviewer suggested, we explored whether more advanced modeling could reveal predictive relationships between Sst-Chodl activity and sleep transitions. We implemented time-resolved GLMs to test whether Sst-Chodl calcium activity predicts the onset of SWS episodes or periods of elevated delta power, and we applied tree-based survival models to estimate the remaining bout duration. Consistent with the strong zero-lag relationship

described above, these models did not show predictive power for future SWS onset, high-delta intervals, or bout duration (see Figure_Reviewer).

Because of the low number of state transitions within each imaging session, classical GLM transition-prediction models could not be robustly fit. As these analyses did not provide additional insight beyond what is now included in the revised manuscript, we have not incorporated them into the manuscript figures.

Together, these new experiments and analyses reinforce the conclusion that Sst-Chodl cells reliably track ongoing synchronized cortical dynamics but do not precede or predict upcoming sleep state transitions. We appreciate the reviewer's suggestions, which significantly improved the clarity and depth of our analysis.

• An analysis that would strengthen the uniqueness of the activation pattern of Sst-Chodl cells is to calculate their correlation with other cell types. This can be done in the Allen dataset by calculating cross-correlograms of putative Sst-Chodl cells against the rest of the population. This may show a strong anti-correlation of activity, while doing the same analysis with other Sst cells (or other cell types) will show a positive correlation.

We thank the reviewer for this excellent suggestion. To further assess the uniqueness of Sst-Chodl cells, we pursued two complementary strategies to compare their activity to other cortical cell types.

First, we expanded our analyses of publicly available datasets. While our original examination of the Allen dataset was confined on the Sst cells and did not capture the diversity of subtypes within this population, we now leveraged the dataset from Bugeon et al. 2022, which contains an expanded and well-defined set of molecularly annotated cell classes, including among others, Sst-Chodl, other Sst subtypes, Vip, Lamp5, and Sncg interneurons as well as excitatory neurons. Consistent with the reviewer's prediction, Sst-Chodl cell activity showed minimal correlation with neuronal populations typically engaged during locomotion, including regular-spiking Sst cells, Vip interneurons, Sncg interneurons, and excitatory neurons (Supp Fig 8). This cross-population comparison highlights the distinct activation signature of Sst-Chodl cells relative to other inhibitory and excitatory cell types in V1.

Second, to test whether other Sst subtypes might exert similar circuit-level effects, we performed additional optogenetic experiments targeting Martinotti neurons, a major subtype of Sst cells that does not express Chodl. In contrast to Sst-Chodl stimulation, activation of Martinotti cells did not induce cortical network synchronization (Supp Fig 12). This functional dissociation further supports the idea that Sst-Chodl neurons form a uniquely specialized Sst subtype capable of promoting cortical synchrony.

Together, these analyses strengthen the conclusion that Sst-Chodl cells exhibit distinctive activation patterns and circuit-level functions not shared by other major Sst populations or by other neurons.

• Were Sst-Chodl cells identified in the silicon probe recording experiments (through optogenetic-tagging)? This would offer the possibility to provide important additional characterization of these cells, including their firing, burstiness and waveform properties. The analysis mentioned in the previous point will be even more convincing using opto-tagged Sst-Chodl cells.

We agree that optogenetic identification of Sst-Chodl cells during silicon probe recordings would offer valuable electrophysiological characterization. In practice, however, this proved technically difficult due to the extremely low abundance of Sst-Chodl neurons. Amongst >700 well-isolated single units and additional multi-unit clusters tested in our recordings, we did not detect clearly identifiable opto-tagged Sst-Chodl cells. This is consistent with their very sparse distribution and with the inherent challenge of targeting rare populations using acute linear probes.

Importantly, we note that an independent collaborative effort led by Josh Siegel at the Allen Institute attempted to opto-tag Sst-Chodl cells using Neuropixels 2.0 multishank probes. Across 8 recording sessions using two NP2.0 probes per experiment, the team obtained 1,738 well-isolated units (an average of ~108 units per probe per insertion). Although two light-evoked responses were detectable in the population, none of the well-isolated units met criteria for opto-tagging. This independent result further underscores the technical difficulty of identifying this extremely sparse neuronal population with current in vivo probe-based approaches.

Although our recordings did not yield tagged units, recent large-scale surveys combining electrophysiology and morphology (e.g., Gouwens et al., 2020) have begun to characterize intrinsic properties of Sst-Chodl neurons, providing useful complementary context for the functional studies presented here.

• SWS is not a homogenous state but rather consists in the alternation between periods of high (UP) and low (DOWN) activity. The authors briefly mention this in Fig. 3 but surprisingly did not perform any analysis of Sst-Chodl cells activity in relation to this fundamental feature of SWS. Such analyses are needed to better understand the role this cell type. Since delta waves (reflecting UP-DOWN transitions) were detected together with 2-photon imaging, it would be possible to analyze Sst-Chodl activity in relation to this two substates: are Sst-Chodl active durign DOWN states contrary to most other cortical cells?, what is the timing of activation of Sst-Chodl cells respect to the DOWN to UP / UP to DOWN transition?, does the activity of Sst-Chodl predict the onset of duration of a subsequent DOWN state?

We thank the reviewer for raising this important point. UP/DOWN dynamics are indeed a defining feature of SWS sleep and understanding how Sst-Chodl cells relate to these fluctuations is essential for clarifying their functional role. As the reviewer notes, our initial dataset posed technical limitations for performing such analyses: the electrophysiological recordings acquired during two-photon imaging did not include local spiking activity which is necessary for accurate DOWN-state detection—and the available LFP signal was recorded contralateral to the imaging site, limiting precise temporal alignment with calcium activity.

To directly address the reviewer's questions, we conducted new experiments using flexible probes implanted within the 2-photon cranial window (as described above). This configuration allowed us to measure Sst-Chodl calcium signals, local UP/DOWN transitions, and multi-unit activity within the same cortical location and with the necessary temporal precision.

These new recordings revealed that Sst-Chodl cells show a distinct temporal profile relative to UP/DOWN transitions. Unlike multi-unit activity, Sst-Chodl cells increase their activity just before UP-to-DOWN transitions. This enabled a series of additional analyses, now incorporated into the manuscript. The corresponding text added to the revised manuscript is provided verbatim below:

“We examined how Sst-Chodl cell activity relates to sleep dynamics on a shorter timescales. Slow-wave sleep is not a homogeneous state but rather consists of rhythmic alternation between “DOWN” phases, characterized by near-complete suppression of spiking during upward deflections of the cortical LFP (DOWN states), and “UP” phases of elevated spiking activity (UP states; Fig 2i)[65]. Aligning Sst-Chodl cell activity to DOWN-state transitions revealed that these cells increased their activity preceding DOWN-state onset, whereas other neurons, simultaneously recorded as multi-unit spiking activity from the same recording site, exhibited heterogeneous responses with prominent rebound firing following DOWN-to-UP transitions (UP onset; Fig 2j-k and Supp Fig 7d). Notably, Sst-Chodl cell activity was not correlated with either the duration or amplitude of the subsequent DOWN state (Supp Fig 7e-f), suggesting that these neurons are preferentially engaged near the termination of cortical UP states rather than during quiescent DOWN phase itself.”

We note that while Sst-Chodl cell activity consistently precedes UP-to-DOWN transitions, we did not find evidence that this activity predicts the duration or amplitude of subsequent DOWN states. This distinction is important, as temporal alignment with a state transition does not necessarily imply predictive control over its future properties. Moreover, predicting DOWN-state parameters from calcium signals is inherently challenging due to the slow kinetics of calcium indicators and the probabilistic nature of UP/DOWN transitions. For these reasons, we interpret our findings as indicating a role for Sst-Chodl cells in the timing of state transitions rather than in determining the properties of the ensuing DOWN state.

• To better understand the network effect of optogenetic stimulation of Sst-Chodl cells the authors should perform peri-stimulation analysis rather than just analyze the changes between stimulation and non-stimulation blocks. That this, they can plot LFP power changes across the depth profile triggered on stimulation pulses, peri-pulse firing histograms (PSTH), etc. If discrete (e.g., single square pulses) pulses were not delivered, similar analysis could be performed with the phase of sinusoidal stimulation pulses. Some of these analyses can also be done with the freely moving EEG/ LFP recordings, which are currently only minimally analyzed.

We thank the reviewer for this helpful suggestion. In response, we performed a set of peri-stimulus analyses to more precisely characterize the network dynamics elicited by Sst-Chodl activation. These new analyses are now included in the revised manuscript.

Specifically, we examining peri-stimulus LFP power changes across cortical depth (Fig 3f), peri-stimulus spiking responses (Supp Fig 9), and the coupling of DOWN-state relative to phase of sinusoidal stimulation (Supp Fig 11a). Across all analyses, we observed a rapid increase in cortical synchrony following stimulation onset. Importantly, this effect was consistent across stimulation types, including square-pulse and sinusoidal photostimulation and did not depend on the phase of sinusoidal stimulation.

Together, these results support the conclusion that Sst-Chodl activation induces a robust and rapid transition into a synchronized cortical state, and that this effect reflects the intrinsic circuit-level influence of Sst-Chodl neurons rather than the specific temporal structure of the stimulation pattern.

• Optogenetic stimulation of Sst-Chodl cells increases the spike coupling to delta waves (Fig. 3g-h). However, the current analyses do not allow to evaluate whether similar or opposite changes happen with other frequency bands. This may be particularly relevant for the high gamma-frequency band (~90-150 Hz) characteristic of deep cortical layers, the same that show the strongest changes in delta power. Both gamma and delta power are associated with UP-

DOWN states. Did the stimulation affected the spiking structure of these states (e.g., unit firing timing within UP states, cell participation and magnitude of activation withing UP states, suppression of firing during DOWN states, etc.).

We appreciate the reviewer's insightful comments regarding potential effects of Sst-Chodl stimulation on additional frequency bands and on the fine structure of UP/DOWN state spiking. In response, we have expanded our analyses substantially, and these new results are now included in the revised manuscript (Supp Fig 9 and 11).

First, we quantified spike-LFP coherence across a broader range of frequency bands, including beta, low gamma, and high gamma (90–150 Hz), and examined these effects across cortical layers. This analysis revealed how Sst-Chodl stimulation modulates the coupling of neuronal spiking to multiple oscillatory components, complementing our original delta-band findings (Supp Fig 9i).

Second, we analyzed DOWN-state coupling strength during optogenetic stimulation, enabling a direct comparison of how Sst-Chodl activation influences the timing and probability of DOWN-state onset (Supp Fig 11a).

Third, we examined spiking structure relative to UP/DOWN transitions, including firing rate dynamics around UP onset, UP offset, and DOWN periods. These analyses allowed us to evaluate unit suppression and rebound relative to DOWN states under Sst-Chodl stimulation (Supp Fig 11b).

Together, these additional analyses provide a more complete picture of how Sst-Chodl activation shapes cortical oscillatory dynamics and the microstructure of UP/DOWN cycles. The results extend and reinforce the conclusion that Sst-Chodl cells promote a highly synchronized network state with coordinated changes across multiple frequency bands and spiking motifs.

- The authors report minimal changes in firing rates due to optogenetic stimulation of Sst-Chodl, but were the spiking dynamics on individual cells affected? This can be evaluated by computing the coefficient of variation of single unit spike trains, the propensity of bursting, etc. Was the excitatory/ inhibitory balance affected?

We thank the reviewer for this thoughtful question regarding potential changes in single-unit spiking dynamics during Sst-Chodl stimulation. To address these points, we have added several new analyses to the revised manuscript (Supp Fig 9).

First, we quantified peri-stimulus firing rate changes, burst propensity, and the coefficient of variation of inter-spike intervals for individual neurons. Across these measures, we found that Sst-Chodl stimulation did not induce systematic changes in spike-timing irregularity or bursting behavior. This result is consistent with the minimal changes in overall firing rates reported in the manuscript.

Second, we analyzed putative excitatory and inhibitory units cross cortical layers to assess whether Sst-Chodl activation alters the excitatory–inhibitory balance. These comparisons likewise revealed no selective or layer-specific changes in either population.

Together, these analyses indicate that while Sst-Chodl activation increases network synchrony, it does so without altering the intrinsic spiking statistics or excitatory–inhibitory balance of local cortical neurons. These findings further support the conclusion that Sst-Chodl cells influence

cortical state primarily through modulation of temporal coordination across neurons rather than by driving changes in firing rate or single-cell spiking structure.

• An accurate way of determined cortical layers is by calculating delta wave-triggered current source density (CSD) maps. This could be done with the silicon probe recordings and have the additional advantage that will allow to compare if the CSD profile during head-fixed recordings has differences to that of the delta waves during natural sleep.

We appreciate the reviewer's suggestion to use delta wave–triggered CSD analysis to refine layer determination and to compare state-dependent profiles across recording conditions. Following this recommendation, we computed CSD maps aligned to DOWN to UP transition from our head-fixed silicon probe recordings. These examples are now included in the revised manuscript (see Supp Fig 11c-d).

The resulting CSD profiles show the expected sink–source pattern associated with UP-state onset and closely resemble those reported in freely moving animals (e.g., Senzai et al., 2019). This alignment confirms both the accuracy of our laminar assignment and the consistency of delta-associated current flow patterns across head-fixed and freely moving preparations.

• A recent paper (Valero et al., Nat Neurosci, 2021) reported a subset of interneurons (neurogliaform cells) in deep cortical layers that were specifically active during SWS DOWN states and shared other similitudes with the pattern of activation of Sst-Chodl cells. These results seem relevant to the present story.

We thank the reviewer for bringing this important study to our attention. The findings from Valero et al. (Nat Neurosci, 2021), describing deep-layer neurogliaform cells active during DOWN states, are indeed highly relevant to our work. We have now incorporated this reference into the revised manuscript and discuss its relationship to the activation patterns and functional properties of Sst-Chodl cells.

• In the description of the state-scoring for head-fixed recordings it would be appropriate to acknowledge that, despite their similitudes, these REM and NREM episodes are not during natural sleep and may therefore have meaningful differences with the equivalent states in freely sleeping animals.

We appreciate the reviewer's point. We have now clarified the Methods section to explicitly note that REM- and NREM-like episodes in head-fixed preparations, while sharing key electrophysiological features with natural sleep, may differ in meaningful ways from freely sleeping states. This distinction is now clearly acknowledged in the revised text.

Referee #2 (Remarks to the Author):

Ratliff et al. investigated the role of cortical long-range projecting SST-positive and Chodl-positive GABAergic cells in awake and sleep states. To address this topic, the authors employed various techniques, including molecular genetic tools, optogenetic and chemogenetic manipulation, calcium imaging, electrophysiological recordings, and behavioral state analyses. The authors demonstrated that a subset of SST cells (SST+, Chodl+) projects to other brain areas, SST-Chodl cells are active during slow-wave sleep, activation of SST-Chodl cells increases delta band in cortical rhythms and induce sleep states.
While most cortical inhibitory cells make synapses locally, a small fraction of neocortical and hippocampal GABAergic cells project to other brain areas. Multiple groups have reported that

SST-Chodl or SST-Nos cells are among the cortical projecting GABAergic cells. These projecting cortical GABAergic cells have received much attention due to their unique projection patterns that can potentially influence global cortical activity. Indeed, several studies have provided evidence that one of the roles of this group may be related to sleep state. Using various methods, the current study demonstrates that this long-range projecting subset of SST cells can increase cortical synchrony in the low-frequency band and induce sleep state. While the current research elegantly demonstrates how SST-Chodl cells control cortical states and sleep behavior, it builds on prior findings that SST-Chodl GABAergic cells project to other brain areas and are associated with slow-wave sleep. This study advances our understanding of projecting GABAergic cells by providing not only correlational but also causal evidence, demonstrating that manipulating these cells affects both cortical and behavioral states. Despite the study's convincing results, there is a concern that this study could be perceived as somewhat lacking in novelty.
The results presented in this manuscript are clear and convincing, and the manuscript is, for the most part, well-written. However, there are a few major and several minor issues. Please see my detailed comments below.

We thank the reviewer for the thoughtful and comprehensive assessment of our manuscript. We appreciate the careful summary of our findings and the acknowledgment that our study provides causal evidence linking Sst-Chodl neuron activity to cortical synchronization and sleep behavior. We also understand the reviewer’s concern that, because Sst-Chodl neurons have been anatomically described previously, the novelty of the present work may not be immediately apparent.

To clarify this point, we would like to highlight that the revised manuscript advances the field beyond prior anatomical and descriptive studies by providing a multi-level, mechanistic characterization of Sst-Chodl cells *in vivo*. Incorporating the reviewer’s suggestions has significantly strengthened the depth, scope, and conceptual clarity of the work.

In particular, the revised manuscript now provides:

- <u>Comprehensive anatomical and synaptic characterization across the full brain</u>**.** We systematically map Sst-Chodl projections and synaptic partners, yielding a detailed anatomical framework for how these neurons are positioned to influence both local and distant cortical circuits.
- <u>Direct demonstration of the functional impact of long-range inhibitory projections in vivo</u>**.** We show that Sst-Chodl cells regulate neuronal activity and synchrony not only locally within V1 but also across distant ipsilateral neocortical regions, revealing a circuit mechanism by which a sparse inhibitory population can exert widespread influence.
- <u>Evidence that Sst-Chodl activation produces physiologically meaningful state-level changes</u>**.** Activating Sst-Chodl cells induces cortical synchrony of a magnitude comparable to natural wake-to-sleep transitions, establishing their relevance for brain-state regulation.
- <u>*In vivo* characterization of Sst-Chodl activity across diverse arousal states.</u> We directly measure Sst-Chodl dynamics during REM sleep, quiet wakefulness, movement, and SWS, providing a comprehensive behavioral-state profile for this population.
- <u>A causal demonstration that a sparse neocortical inhibitory subtype can promote sleep-related dynamics.</u> By combining cell-type-specific imaging, electrophysiology, optogenetics, and chemogenetics, we show that Sst-Chodl cells are sufficient to bias cortical networks toward synchronized states and promote sleep behavior.

Taken together, these results establish Sst-Chodl cells as an integral neocortical circuit element capable of coordinating local and distributed network dynamics associated with sleep. We believe that the breadth of approaches and the mechanistic integration achieved here substantially enhance the novelty and significance of the study.

Major comments

1. The current study demonstrates two unique properties of SST-Chodl GABAergic cells: first, these neurons project to other brain areas despite being GABAergic, and second, their activity can influence cortical synchrony in the delta band range and slow-wave sleep (SWS) states, even though this group constitutes less than 1% of cortical GABAergic cells. However, it is unclear how these two features are interconnected. My understanding is that the long-range axonal projections of these neurons allow them to regulate broad cortical areas despite their small number. However, this study does not establish a direct link between their projection to other brain areas and the control of widespread cortical activity. Connecting these two properties is crucial for understanding the role of the long-range axonal projections. Alternatively, it is possible that delta band synchrony and SWS states are not induced by long-range SST-Chodl cell innervation but rather by local arborization and synapses from these cells. To address this, I suggest investigating how the activation of SST-Chodl cells in one cortical area affects delta band power in other cortical areas.

We thank the reviewer for this insightful and important comment. We fully agree that establishing a direct functional link between the long-range axonal projections of Sst-Chodl cells and their ability to influence widespread cortical synchrony is essential for understanding their role. While these neurons have been anatomically described as long-range projecting GABAergic cells, whether, and how,

Addressing this precise gap is a central advance of the revised manuscript. To directly test whether the widespread synchronization induced by Sst-Chodl activation reflects their long-range projections rather than purely local effects, we performed three complementary sets of new experiments. These data are now presented in a new main figure (Figure 4) and two new supplementary figures (Supplementary Figures 10 and 13), and together they provide converging functional evidence that the long-range axonal architecture of Sst-Chodl cells is directly linked to their capacity to modulate distributed cortical activity. This distinctive anatomy enables them to regulate cortical dynamics beyond their local arborization has remained unresolved.

<u>1. Local stimulation produces synchronized activity across millimeter-scale cortical distances.</u> Using multishank silicon probes, we stimulated Sst-Chodl cells at a single shank while simultaneously recording spiking activity and LFPs across several sites in the visual cortex. Despite local stimulation, we observed robust increases in delta-band power and spiking synchrony across all shanks, extending over ~1 mm from the stimulation site (Supp Fig 10). This demonstrates that the influence of Sst-Chodl cell activation is not spatially restricted to the immediate vicinity of light delivery, and that activation can influence synchrony across visual cortical regions on the millimeter scale.

<u>2. Sst-Chodl activation modulates synchrony across distant cortical regions.</u> To examine the broader spatial footprint, we used custom-built multisite electrodes located substantially farther from the stimulation site than in our multishank probe experiments, spanning ~2-6 mm from the stimulation site. Optogenetic activation in V1 increased produced the strongest enhancement of low-frequency power near the stimulation site, but importantly, synchrony also increased ~4.5 mm away, including in frontal regions such as the motor cortex (Figure 4f-i). The graded

decrease in effect strength with distance mirrors the anatomical density of Sst-Chodl projections, which are highest near their home region and sparser distally, providing a functional correlate of their projection architecture.

3. Long-range Sst-Chodl projections form functional synapses at distant cortical sites. To directly test whether these long-range axons establish effective synaptic contacts, we performed additional whole-cell patch-clamp recordings in acute slices at increasing distances from the injection site. Optogenetically evoked inhibitory postsynaptic currents were detected in nearly one-third of recorded neurons located approximately 2 mm away, and in neurons located at the farthest distance tested, up to 2.4 mm from the injection site (Fig. 4a–e, Supp. Fig. 13). These findings demonstrate that Sst-Chodl long-range projections form functional inhibitory synapses across distributed cortical regions, providing a synaptic substrate through which these neurons can influence cortical activity beyond their local arborization.

Together, these new experiments directly address the reviewer's concern by linking the long-range axonal architecture of Sst-Chodl cells to their ability to modulate cortical synchrony beyond local circuits. By demonstrating distance-dependent, synaptically mediated effects on neural activity and low-frequency oscillations across multiple cortical areas, the revised manuscript establishes that the widespread synchronization induced by this sparse neuronal population is tightly coupled to their long-range projections, rather than arising solely from local arborization.

2. The authors performed slice electrophysiology to map how SST-Chodl cells locally target other cells within V1 across cortical layers. Related to Major Comment 1, understanding how SST-Chodl cells target neurons in other cortical areas is crucial for elucidating their role in intercortical interactions.

We thank the reviewer for raising this important point. As detailed in our response to Major Comment 1, we performed additional experiments specifically designed to determine how Sst-Chodl cells influence neurons in distant cortical areas. These new data now demonstrate that:

- Sst-Chodl activation modulates LFP power and synchrony several millimeters from the stimulation site, including in frontal cortical regions.
- Functional inhibitory synaptic currents can be evoked in neurons located up to the farthest location we tested, 2.4 mm away from the injection site, indicating that long-range projections make effective synaptic contacts across distant cortical areas.
- The spatial decay of the functional effect closely matches the anatomical density of Sst-Chodl projections.

These results directly address the reviewer's concern by showing that Sst-Chodl neurons not only target local cells within V1 but also form functional connections and exert measurable physiological influence across distributed cortical regions.

3. The authors reported that SST-Chodl cells project to other cortical areas in a non-uniform manner, with uneven innervation patterns; for example, there is dense innervation in the RL area (Figure 1f, g). However, the injection sites in V1 are spatially biased, predominantly located in the anterior part of V1. To determine whether this uneven innervation is due to injection bias or reflects the inherent projection pattern of SST-Chodl cells, it would be useful to label SST-Chodl cells in different parts of V1 (posterior or lateral) and analyze their axonal innervation in other cortical areas.

We thank the reviewer for raising this excellent point regarding potential injection-site bias. To directly address this concern, we performed additional experiments and reconstructed 16 individual Sst-Chodl cells across the entire mouse brain using Micro-Optical Sectioning Tomography. This approach enabled us to map long-range axonal projections with single-cell resolution and without reliance on local viral injection sites.

These reconstructions reveal that Sst-Chodl neurons located in different parts of V1, including anterior, posterior, medial, and lateral regions, as well as Sst-Chodl neurons located outside V1 (e.g., in somatosensory cortex, Supp Fig 4) exhibit consistent projection principles and target largely overlapping sets of cortical regions (Fig 1 and Supp Fig 2). Thus, the uneven innervation patterns observed in our initial dataset are not attributable to injection bias but instead reflect intrinsic projection rules of this cell type.

For comparison, we also reconstructed other Sst subtypes and Pvalb cells, which further highlights the distinct long-range anatomical profile of Sst-Chodl cells.

Together, these additional experiments demonstrate:

1. Consistency across areas: Sst-Chodl projection rules are highly conserved regardless of precise somatic position within V1 or across other cortical regions.
2. Accurate estimation of projection strength: The density of labeling across regions reflects genuine differences in Sst-Chodl arborization, not technical variability.
3. Principles of single-cell targeting: Individual Sst-Chodl neurons follow a reproducible logic in the set of regions they innervate, underscoring a structured long-range communication pathway.

These results directly address the reviewer's concern and significantly strengthen our understanding of how Sst-Chodl neurons distribute their projections across the cortex.

4. Figure 1: The data in Figure 1 and from other studies demonstrated that SST-Chodl cells innervate other cortical regions. In current study, the analysis of SST-Chodl cell axonal innervation is performed at the population level. However, it is difficult to understand the extent to which individual SST-Chodl cells project to other cortical areas. How many SST-Chodl cells were labeled at each injection site? How does a single SST-Chodl cell innervate other brain regions?

We thank the reviewer for raising this important point. To directly address the extent to which *individual* Sst-Chodl neurons project to other cortical areas, we reconstructed 16 single Sst-Chodl neurons in their entirety using high-resolution Micro-Optical Sectioning Tomography. These neurons were sampled from multiple anatomical locations, including anterior and posterior V1 as well as several higher visual areas (Fig 1, Supp Fig 2). These single-cell reconstructions provide a detailed and accurate view of how individual Sst-Chodl cells distribute their axons across the brain.

These data reveal that a single Sst-Chodl neuron can innervate multiple cortical regions, often spanning both local visual areas and more distant cortical structures. While the density of innervation varies across targets, the overall projection logic is similar across cells, regardless of their exact soma location. This analysis clarifies the structure of long-range communication at the single-cell level.

In parallel, our population-level labeling strategy, which yielded ~20 detected Sst-Chodl neurons per brain on average during our preparation, allowed us to quantify the aggregate arborization

patterns across the cortex (Supp Fig 3h). These population data are complementary to the single-cell reconstructions: together, they demonstrate both (1) the overall distribution of projections across cortical areas and (2) the specific projection profiles of individual neurons.

Thus, our dataset now provides a comprehensive view of the long-range axonal organization of Sst-Chodl cells at both the *population* and *single-cell* levels, addressing the reviewer's questions.

5. Supplemental Figure 4: It is interesting to note that L6 pyramidal neurons receive strong inhibition from SST-Chodl cells. Please provide the distribution of ChR-expressing SST-Chodl cells to ensure that this finding is not due to injection bias.

We thank the reviewer for this insightful comment. To ensure that the strong inhibition observed in layer 6 pyramidal neurons was not an artifact of injection bias, we have added new data addressing both the distribution of ChR2-expressing Sst-Chodl cells and the laminar pattern of evoked responses.

First, we now include example images showing the ChR2-expressing Sst-Chodl axons overlaid with anatomical reconstructions of recorded post synaptic neurons (Fig 4c). These images illustrate our injection strategy in which 50% of the viral volume was delivered to superficial layers and 50% to intermediate layers, specifically to avoid laminar bias at the injection site.

Second, we quantified oIPSC amplitude across cortical layers at multiple distances from the injection site. Notably, layer 6 consistently displayed larger oIPSC than neurons in other layers, and this effect was stable across all tested distances. These results argue against a layer-specific injection artifact.

Our anatomical reconstructions further show that Sst-Chodl long-range axons are not uniformly distributed: projections are relatively sparse outside the injection site and are largely confined to upper layers (Figure 1, Supp Fig 3 and Supp Fig 13). Given this projection pattern, the robust and distance-independent responses in layer 6 pyramidal neurons recorded *outside* the injection site strongly suggest that the observed inhibition reflects a genuine physiological property rather than technical bias.

Finally, we note that inhibitory responses in L6 pyramidal neurons were markedly variable, with oIPSC amplitudes ranging from ~20 to 500 pA. This variability may reflect the substantial molecular and electrophysiological diversity within the L6 pyramidal neuron population. Future work will be needed to determine the specific features that predict sensitivity to Sst-Chodl input.

Together, these analyses indicate that the strong inhibition of layer 6 pyramidal neurons by Sst-Chodl cells is a biological property, not an injection artifact, and likely reflects a distinctive component of Sst-Chodl circuit function.

6. Figure 2: Please provide the imaging depth for the 97 SST-Chodl cells that were imaged. Is there any relationship between the recording depth and the Δf/F of each SST-Chodl cell?

We thank the reviewer for raising this important question regarding the laminar distribution and activity patterns of imaged Sst-Chodl neurons. In our initial dataset, all recorded cells were located in superficial cortical layers. This reflects a technical limitation of conventional two-

photon imaging through a standard cranial window, where scattering and signal attenuation make reliable deep-layer imaging difficult.

To overcome this limitation and directly test whether Sst-Chodl activity profiles differ across layers, we performed additional imaging experiments using microprisms, which provides simultaneous optical access to neurons across the full cortical depth. Using this approach, we were able to image Sst-Chodl cells spanning across all layers.

These experiments revealed that Sst-Chodl cells exhibit highly similar ΔF/F activity patterns across layers, both in their modulation by arousal state and in their relationship to synchronized cortical dynamics (Fig 2; Supp Fig 6). Thus, the activity profiles reported in our original dataset are representative of Sst-Chodl cells throughout the cortical column.

Together, these new experiments indicate that the relationship between Sst-Chodl activity and brain state is consistent across layers, and not driven by sampling bias toward superficial neurons.

7. Figure 3: The optogenetic stimulation of SST-Chodl cells induces cortical synchrony. Is this function unique to SST-Chodl cells or other SST-positive, Chodl-negative cells share a similar function. Please provide evidence to demonstrate that increasing cortical synchrony is a specific feature of SST-Chodl cells.

We thank the reviewer for this important question regarding the specificity of Sst-Chodl neurons in generating cortical synchrony. To directly test whether this function is unique to Sst-Chodl cells or shared with other Sst subtypes that do not express Chodl, we performed two complementary analyses.

1. Optogenetic activation of Martinotti cells does not induce cortical synchrony. Using recently developed enhancer viruses to selectively target Martinotti neurons, a major Sst-positive, Chodl-negative population, we performed optogenetic stimulation under conditions identical to those used for Sst-Chodl experiments. In contrast to the robust increase in delta-band power and network synchrony observed with Sst-Chodl activation, Martinotti stimulation produced no detectable changes in cortical synchrony (new data included in Supp Fig 12). This establishes that the ability to drive synchronized low-frequency activity is not a general property of Sst cells, but is specific to the Sst-Chodl subtype.

2. Comparative activity analyses with other neuronal populations. As part of a broader comparison (see also Reviewer 1, Comment #2) we analyzed public datasets and computed cross-correlograms between identified Sst-Chodl cells and other neuronal populations. (Supp. Fig 8). Sst-Chodl activity showed little correlation or negative correlation with populations typically active during locomotion (e.g., Vip, Sncg, regular-spiking Sst, excitatory neurons). This reinforces our observation that Sst-Chodl neurons exhibit a distinct activation profile relative to other inhibitory and excitatory cell classes.

Together, these new experiments and analyses demonstrate that Sst-Chodl neurons, but not other major Sst subtypes, can enhance synchronized low-frequency cortical dynamics, supporting a unique functional specialization within the Sst family.

8. Figure 3, Supplemental figure 7: The slice electrophysiology results demonstrate that inhibition from SST-Chodl cells is layer-dependent, with stronger inhibition observed in layer 6 pyramidal cells. Given this finding, please provide the analysis of the spike-LFP coherence

based on cortical layers (Figure 3) and the firing rate changes based on layers (Supplemental figure 7).

We thank the reviewer for this helpful suggestion. In response, we have expanded our analyses to explicitly examine both spike–LFP coherence and firing rate changes across cortical layers. First, we now provide layer-resolved spike–LFP coherence across multiple frequency bands, including delta, theta, beta, and gamma (Supp Fig 9). This analysis reveals how Sst-Chodl activation modulates rhythmic coupling in each cortical layer and allows direct comparison with the layer-dependent synaptic inhibition observed in slice recordings.

Second, we have divided the firing rate analysis by layer, enabling us to assess whether changes in spiking activity during stimulation differ across the cortical depth (Supp Fig 9). These analyses clarify that, despite stronger inhibitory postsynaptic currents in layer 6 pyramidal neurons, the population-level firing rate changes remain modest and broadly consistent across layers.

Together, these additions directly address the reviewer's request and strengthen the connection between the slice physiology and *in vivo* network-level effects.

9. Figure 3, Supplemental Figure 7: The duration and amplitude of down-states were increased during optogenetic stimulation, yet the event rate remained unchanged. If the duration of down-states is increased, the event rate should typically decrease. Please clarify this discrepancy.

We thank the reviewer for pointing out this apparent discrepancy. To clarify this issue, we performed additional experiments and expanded our dataset. With the increased statistical power, we now find that optogenetic activation of Sst-Chodl cells increases both DOWN-state duration and DOWN-state event rate.

Although this may appear counterintuitive, these effects are not mutually exclusive. UP/DOWN dynamics are not strictly rhythmic, and transitions do not follow a fixed oscillatory period. Instead, the timing of DOWN-state initiation is highly variable (Levenstein et al., 2019). As a result, DOWN states can become longer while also occurring more frequently if UP states shorten, fragment, or terminate earlier, independently of DOWN-state duration.

Thus, increased DOWN-state event rate does not imply shorter DOWN states, nor does prolonged DOWN-state duration necessarily predict fewer events. Rather, our results suggest that Sst-Chodl activation biases cortical dynamics toward more frequent and sustained transitions into DOWN states without imposing a fixed rhythmic structure. These clarifications and updated analyses are now included in the revised manuscript (Supp. Fig. 9).

10. Figures 3, 4: Optogenetic stimulation increased delta power during slow-wave sleep (SWS) and quiet wakefulness (QW), while chemogenetic stimulation increased delta power during SWS and wakefulness. It appears that chemogenetic stimulation has a stronger effect on delta power during wakefulness compared to optogenetic stimulation. Please provide a separate analysis of delta power changes during QW and movement under chemogenetic stimulation.

We thank the reviewer for this constructive suggestion. In response, we have now performed a separate analysis of delta-band power during quiet wakefulness (QW) and during movement under chemogenetic stimulation.

These analyses are now included in the revised manuscript (Fig 5 and Supp Fig 14). We show that chemogenetic activation of Sst-Chodl cells significantly increases delta power during both QW and movement, in addition to SWS, thereby directly addressing the reviewer's request for state-resolved quantification.

11. Supplemental Figure 9: It appears that the calcium activity of individual SST-Chodl cells was recorded using 2-photon (2PT) calcium imaging under head-fixed conditions. Please specify the imaging conditions clearly. Since panel 'a' shows data from a freely moving home cage condition, this could be confusing. If the data is with 2PT imaging under head-fixed conditions, please provide a population activity analysis of SST-Chodl cells following CNO injection.

We thank the reviewer for pointing out the potential confusion regarding experimental conditions. As suggested by the reviewer, we have now clarified the figure and legend, specifying that Supp Fig 14b-c are associated with calcium imaging and the rest of the figure with freely moving conditions.

In addition, in response to the reviewer's suggestion, we have performed and included a population-level analysis of Sst-Chodl calcium activity following CNO administration under two-photon imaging conditions. This analysis is now provided in Supplementary Figure 14 and demonstrates that chemogenetic manipulation increase Sst-Chodl cell activity independently of the animal state.

These clarifications and additional analyses ensure that the imaging modalities and experimental conditions are fully transparent to the reader.

Minor comments
Figure 1: Please provide the distribution of SST-Chodl cells across cortical layers in V1.

We thank the reviewer for this helpful suggestion. We have now added the laminar distribution of Sst-Chodl cells in V1 based on cells in our experiments (see updated Figure 1 and Supplementary Table 1 and 2). This distribution is consistent with the detailed characterization reported in Gouwens et al. (2020), which we now cite explicitly in main text.

Figure 3: It appears that this set of experiments was conducted under head-fixed conditions. Please clearly state the experimental conditions used.

We thank the reviewer for noting this ambiguity. We have now clarified the experimental conditions for the Figure 3 experiments, and for other figures where this information was missing, by explicitly stating that these recordings were performed under head-fixed conditions. The corresponding figure legends have been updated accordingly.

Figure 3dleft: please label x-axis.

We thank the reviewer for pointing this out. We added this label.

Figure 3g: It seems that the increased spike-LFP coherence extends beyond the delta band range. Please provide statistical analysis on that.

We thank the reviewer for this observation. We have now performed a full statistical analysis of spike–LFP coherence across all frequency bands, not only delta. These results are included in the revised manuscript (see Supp Fig 9 and accompanying text). The analysis confirms that Sst-

Chodl stimulation significantly increases coherence in the delta range and also produces measurable increases in adjacent low-frequency bands.

Figure 4: Please indicate that the LFP recordings are from V1.

We thank the reviewer for this helpful clarification. We have now explicitly indicated in Figure 5 (Figure 4 in the prior version) and its legend that the LFP recordings were obtained from V1.

Supplemental figure 9f: it appears that SWS data is missing.

We thank the reviewer for pointing this out. We have now clarified the graph to ensure that the SWS data are properly displayed and clearly distinguishable.

Referee #3 (Remarks to the Author):

This is an interesting and potentially important manuscript reporting on the function of a rare neocortical cell type, GABAergic Sst/Chodl/NOS1 cells, which send long range projections throughout the cortex. For some years, researchers have been speculating on the function of these rather exotic cells, and based on the work of one of the co-authors (TS Kilduff) there was a strong anticipation that this particular cell type would be involved in generating sleep. But these cells have been difficult to study because of their sparseness and the requirement for intersectional genetics (to set them apart from all the other sst cell types). Ratliff, Terral et al report on an impressive technical demonstration that the cells can be genetically targeted and functionally investigated. The overall findings reported are interesting, but the key observations presented in Figure 4, that these cells promote NREM sleep, however, seem quite subtle. The data are not very persuasive that these cells are major players in generating sleep. Alternatively, perhaps there is just too much variation in the current data set, or that there are subtypes of Sst/Chodl/NOS1 that do different things. These issues should be resolved in any revised manuscript.

We thank the reviewer for their thoughtful and candid assessment of our work. We especially appreciate the reviewer's perspective on the long-standing interest in Sst/Chodl/NOS1 cells and the challenge of studying such a sparse and specialized neuronal population. As the reviewer notes, establishing whether these neurons truly contribute to sleep regulation is a central and important question, and we are grateful for the opportunity to strengthen our manuscript in this regard.

In revisiting our dataset in light of the reviewer's comments, we realized that a key factor influencing the apparent effect size in our original chemogenetic experiments was the circadian timing of CNO administration. Our initial manipulations were performed during the light phase, when baseline sleep propensity in mice is already high, limiting the dynamic range available for detecting increases in sleep. Prompted by the reviewer's insight, we performed an additional cohort of experiments in which CNO was administered during the dark phase (ZT18), when sleep drive is low and wake predominates. Under these conditions, activation of Sst-Chodl cells produced a marked increase in total sleep time, revealing a robust and physiologically meaningful effect that was less apparent in the light-phase recordings. These results are now included in the revised Figure 5 and Supp Fig 16.

In addition to strengthen our chemogenetic experiments, we also:

- Increased the number of animals in the original cohort (from 8 to 14 animals), which reduced variability and confirmed that most individual animal shows increased sleep with Sst-Chodl activation.
- Highlight in the revised text that Sst-Chodl activation produces cortical synchrony of a magnitude comparable to natural SWS sleep, and remarkably, this synchronization emerges even when animals are actively moving (Fig 3n).
- Show that Sst-Chodl activation during dark/active phase increases sleep to comparable, if not beyond, levels observed under unperturbed (Vehicle) light/inactive phase condition.

Finally, as the reviewer correctly notes, several experimental factors, including the sparse distribution of Sst-Chodl neurons and the necessity of using relatively low CNO doses to avoid off-target sedation, may have contributed to the variability seen in the original dataset. We now discuss these considerations transparently in the revised manuscript, not as limitations of the conclusion, but as context that helps explain the range of effect sizes observed across animals.

We thank the reviewer again for their constructive skepticism, which directly motivated critical additions to our study. We believe that the resulting dataset presents a much clearer picture of the role of Sst-Chodl neurons in sleep regulation.

Specific points:
1. Numbers and variability. No power calculations were done. However, in general, some of the n numbers are particularly low, but at the same time, the experimental setting is complex. For example, for the experiments with the tamoxifen-independent conditional Cre recombination mice, using just 3 or 4 mice for in vivo experiments seems low. Although the overall statistics appear significant in key points, the individual plots, before and after, consistently show high variability throughout the optogenetics and chemogenetic experiments.

We thank the reviewer for highlighting the importance of sample size and variability in our in vivo experiments. We fully agree that, given the sparseness of the Sst-Chodl population and the complexity of the experimental paradigms, adequate sampling is essential for drawing robust conclusions.

Prompted by the reviewer's insightful comment, we have expanded our datasets across all in vivo manipulations, including both optogenetic and chemogenetic experiments. The number of mice in our conditional Cre recombination cohorts has now been increased from 3–4 animals to up to 10 animals, and we likewise added additional subjects across other stimulation paradigms (updated in all relevant figures and legends).

With the inclusion of these additional animals:

- All population-level effects remain statistically significant or have become more robust, and
- Most of individual animal shows effects in the same direction, even though the magnitude varies.

We note that some degree of variability is expected in these experiments due to factors inherent to the biology of this cell type, including its extreme sparsity, and the necessity of multisite cortical injections.

We are grateful to the reviewer for prompting us to expand these datasets, which has strengthened the statistical confidence and interpretability of the findings.

2. Chronic optogenetics with Ca2+-permeable CatCh by delivering LED stimulation above a thinned skull is an interesting approach; however, this was done in only 3 animals in one brain area. Furthermore, at least, in the example trace in Suppl Fig8b, the delta power is already looking like its increasing even before stimulation. Giving 1s 25mW LED pulses at 0.5Hz for over 1 hour would likely heat up the area generally. Therefore, increasing the cohort size, as well as using negative control experiments in different parts of the cortex would be necessary.

We thank the reviewer for this thoughtful assessment of the chronic optogenetic CatCh experiment. We agree with the reviewer that the cohort size, together with potential confounds such as slow drift in delta power and heating effects during prolonged illumination, limit the interpretability of this specific dataset. The central conclusion that Sst-Chodl activation promotes cortical synchrony is already supported by our acute optogenetic, chemogenetic, and electrophysiological experiments, all of which use well-controlled stimulation paradigms and larger sample sizes. Because the CatCh experiment was designed primarily as an exploratory complement, and not as essential evidence for our main claims, we have decided to remove this figure from the revised manuscript. We appreciate the reviewer's careful reading and helpful suggestion, which ultimately strengthened the clarity and focus of the overall study.

3. For the "whole cortex excitatory DREADD experiment", the authors injected AAV in multiple sites of cortex to cover wide areas. A technically impressive thing to do. The results in Fig. 4, however, are not compelling that these cells are a major driver of sleep (even though I appreciate that the overall stats show significance). Following CNO, the total duration of sleep is extended by only about 10 mins (Fig. 4c and d), and the time course of this accumulation is slow. These are hardly large effects. While the example trace given in Fig 4b seems to be a striking effect on sleep behaviour induction after CNO injection, including sleep onset shift by around 20mins, the individual plots in Fig4e shows only half the case of rough 20min changes. In fact, individual before-after plots in Fig 4d-h appear the effect is widely variable. This variability is also clear in Suppl Fig 9d.

We thank the reviewer for this important point regarding the modest effect size and variability in the initial whole-cortex chemogenetic experiments. As noted above, this concern directly motivated additional experiments in which Sst-Chodl cells were activated during the dark (active) phase (ZT18), when baseline sleep drive is low. Under these conditions, Sst-Chodl activation produced a substantially larger and more consistent increase in sleep across animals (revised Fig. 5; Supp. Fig. 16).

We additionally increased the sample size of the original light-phase cohort and revised the presentation to emphasize population-level analyses rather than individual examples, replacing the previously shown example session with more representative traces. Together, these additions clarify that the initially modest effect reflected experimental context rather than a limitation of Sst-Chodl function

4. In Fig. 4d, SWS plot, it looks like maybe eight instances of total time in SWS are decreasing following CNO administration. Similarly, in the wake graph, there are 7 or 8 instances of wake increasing. This might suggest that there are two subtypes of Sst-Chodl cell? One promoting wake, the other SWS? The authors should try to resolve this.

We thank the reviewer for raising this thoughtful question. In our original dataset, the small number of sessions contributed to the appearance of variability in the before–after plots for SWS and wake. To clarify this point, we have now substantially expanded the sample size

allowing us to plot animal level data rather than session level for both light- and dark-phase manipulations.

With the updated dataset:

- 13 of 14 animals in the light/inactive phase cohort show an increase in SWS following CNO.
- 6 of 6 animals in the dark/active phase cohort (ZT18) show an increase in SWS.

These results indicate that, at the animal level, the sleep-promoting effect of Sst-Chodl activation is highly consistent, with only a single mouse showing a minimal decrease during the light/inactive phase.

We appreciate the reviewer's question regarding whether bidirectional effects could suggest distinct functional subtypes of Sst-Chodl neurons. To address this, we examined both our imaging data and available transcriptomic evidence:

- Single-cell RNA-sequencing studies consistently identify only one Sst-Chodl subtype in the adult cortex (Micoli et al. 2025). This includes large-scale datasets in which inhibitory neuron diversity is well resolved (Yao et al. 2021). In our imaging dataset, the vast majority of Sst-Chodl cells (>85%) increase activity during low arousal or SWS, forming a highly coherent physiological group.
- A small fraction of cells (~<15%) exhibited divergent activity patterns. These may reflect biological heterogeneity within the targeted population, potentially including Type II nNOS-expressing interneurons that express lower or variable levels of Sst and Nos1 (Perrenoud et al., 2012)
- Consistent with this, our microprism imaging across all cortical layers revealed that Sst-Chodl neurons exhibit similar state-dependent activity profiles throughout the cortical depth, providing no evidence for layer-specific subpopulations with opposing functions.

Taken together, the transcriptomic, anatomical, physiological, and behavioral data all support the conclusion that adult Sst-Chodl neurons predominantly promote SWS sleep when activated. We thank the reviewer for prompting this deeper analysis, which has strengthened our interpretation and is now clearly reflected in the revised manuscript.

5. In Fig. 4c (cumulative sleep time), in addition to the saline control, it would be good to see the effect of CNO on sleep into the Cre/Flp mice that are not expressing the CNO-DREADD receptors.

In response, we have now included the cumulative sleep-time analysis for Cre/Flp mice that do not express the DREADD receptor following CNO administration. These data are presented in Supplemental Figure 15c-g and confirm that the low dose (0.5mg/kg) of CNO that we used does not alter sleep in DREADD-negative animals.

This addition strengthens the conclusion that the observed sleep effects result specifically from Sst-Chodl activation rather than off-target pharmacological actions of CNO.

6. The authors state that no differences in sleep behavior were detected between the time of the day used for the DREADD experiments (line 746-7). However, they selected injection timing only at ZT3, 5, and 7. While the animals are likely sleepy at these time points, what happens if Sst-Chodl cells are selectively activated in the (lights-off) wakeful time period of the mice?

We thank the reviewer for this extremely helpful observation. The reviewer's comment made us realize that restricting our chemogenetic manipulations to ZT3–7, when mice already exhibit high baseline sleep, limited the dynamic range for detecting the full contribution of Sst-Chodl cells. This was an important oversight on our part, and we are grateful to the reviewer for prompting us to revisit this question.

Guided by this insight, we performed an additional cohort of experiments during the dark/active phase (ZT18), when mice are naturally awake and sleep pressure is low. These new experiments revealed a substantially larger sleep-promoting effect of Sst-Chodl activation: we found that animals spent about 50% of their time in SWS after CNO administration, effectively doubling the amount of sleep relative to Vehicle administration (Figure 5m, Supp Fig 16).

This result directly addresses the reviewer's question and, importantly, demonstrates that the capacity of Sst-Chodl neurons to promote sleep is much more apparent when they are activated at a physiologically appropriate circadian time. We believe this insight significantly strengthens the overall contribution of the study.

We thank the reviewer again for bringing forward this critical point, which substantially improved the rigor and interpretability of our conclusions.

7. Another possibility: Perhaps the Sst-Chodl cells are following NREM sleep rather than being primary generators? In Fig. 2d, the calcium trace in SWS has a profile which slowly increases during SWS; this does not really look like the profile of a "driver cell".

We thank the reviewer for raising this insightful possibility. Reviewer 1 also highlighted the importance of determining whether Sst-Chodl neurons *follow* SWS dynamics or actively participate in shaping them. We fully agreed with this concern and, motivated by these comments, performed a series of additional experiments and analyses now included in the revised manuscript (Fig 2, Supp Fig 6-7, see also Figure_Reviewer).

Using simultaneous two-photon calcium imaging and local electrophysiology with high–temporal resolution, we examined the precise timing of Sst-Chodl activity relative to SWS onset and to the finer structure of cortical UP/DOWN transitions. These new data show that Sst-Chodl activity rises tightly with increases in delta power, but it does not *precede* the onset of NREM, consistent with the reviewer's suggestion.

Interestingly, compared to other cells (multiunit activity), Sst-Chodl neurons show a highly specific pattern of state-related activity, marked by increased activity prior DOWN states (at UP state termination) but no rebound activity during DOWN-to-UP transitions (Fig 2; Supp Fi. 7).

Thus, rather than being simple "followers" of SWS, these results suggest that Sst-Chodl neurons could participate in stabilizing or consolidating the synchronized cortical dynamics characteristic of SWS, specifically by engaging at the termination of UP states – a possibility that we included in the discussion.

We are grateful to the reviewer for prompting these important analyses, which have strengthened both the manuscript and our mechanistic understanding of how this rare interneuron population participates in sleep-related cortical dynamics.

8. In this regard, it is interesting to speculate what activates these cells? Presumably glutamate? Is it possible they might receive excitatory inputs from thalamic relay cells, which are active

during SWS, which then drive Sst-Chodl to further synchronize cortical activity? In that sense, the SWS-active Sst-Chodl cells would be acting to reinforce neocortical synchrony, but not be primary drivers. Could this be examined by looking at inputs to the Sst-Chodl cells e.g. with rabies-type methods?

We thank the reviewer for this thoughtful and stimulating question. Understanding what activates Sst-Chodl cells during NREM sleep is indeed an important next step, and we agree that identifying their inputs is essential for refining their mechanistic role.

To address this, we performed conditional monosynaptic rabies tracing and obtained a comprehensive map of presynaptic partners (Supp Fig 5). We found that the vast majority of inputs arose from local intracortical neurons, consistent with their extensive local and long-range cortical connectivity. In contrast, thalamic inputs were relatively sparse (<8%) and no inputs from the thalamic reticular nucleus were encountered.

These anatomical results suggest that Sst-Chodl neurons are predominantly driven by cortical excitatory signaling rather than by thalamic relay activity. At the same time, we note that rabies-based approaches are not exhaustive, and despite our best efforts, we cannot exclude the possibility that certain excitatory inputs, especially those that are weak, distal, or sparse, may not have been captured with this method. Given previous evidence that these cells express a wide range of neuromodulatory receptors, it is likely that they receive additional (potentially non-synapti) inputs from other sources (Paul et al. 2017; Endo et al. 2014; Williams et al. 2017), a limitation that we now discuss in the revised manuscript.
Our new high–temporal resolution imaging and electrophysiological analyses complement this anatomical picture. We find that:

- Sst-Chodl activity tracks ongoing cortical synchrony tightly and increases specifically around UP to DOWN transitions (Fig 2; Supp Fig 7).

Optogenetic stimulation of Sst-Chodl neurons rapidly enhances delta power, demonstrating that these cells can reinforce synchronized network states once they are activated.

Minor points:
9. Please consider adding NOS1 somewhere into the name of these cells, so instead of calling them “Sst-Chodl”, they might, for example, be called “Sst-NOS1-Chodl” instead. This makes the name easier to relate to the large body of previous work by Kilduff et al on NOS1 cells in cortex and sleep.

We thank the reviewer for this thoughtful suggestion and appreciate the historical importance of NOS1-expressing interneurons in the study of cortical sleep mechanisms, particularly through the influential work of Kilduff and colleagues. As the reviewer notes, NOS1 has long served as a defining molecular marker in this literature.

In recent years, however, large-scale transcriptomic and anatomical surveys (e.g., BICCN, Tasic et al. 2018) have converged on Chondrolectin (Chodl) as the primary and most specific marker for this rare long-range projecting Sst-expressing inhibitory population. For this reason, “Sst-Chodl” has become the current standard nomenclature in cell-type classification frameworks, and we chose to adopt it to remain consistent with these community-wide datasets.

That said, we fully agree that the connection to the NOS1 literature is important for clarity and continuity. In response to the reviewer’s suggestion, we have revised the text to explicitly note that these neurons correspond to the NOS1-expressing population described in earlier work, and we highlight this equivalence at the first mention of the cell type in the Introduction. This

strikes a balance between maintaining consistency with contemporary nomenclature and honoring the substantial body of foundational work linking NOS1 interneurons to sleep.

10. Line 311. Sst-expressing cells and sleep prep behaviour (ref 60). The authors write: “It remains to be seen whether those Sst cells correspond to the Sst-Chodl cells”. It seems likely that they do not. The fos-trapped sampled prefrontal cortex cells in that paper (ref 60) were reported as all Sst positive, all Chodl negative and only a few as Nos1-positive (and also had the capacity to fire at a relatively fast rate); whereas, as a positive control, chodl expression was found in randomly sampled Sst cells.

We thank the reviewer for pointing out this discrepancy. We agree that the Fos-trapped Sst cells reported in ref. 57 (60 in previous version) are unlikely to correspond to the Sst-Chodl population. As the reviewer notes, those cells were uniformly Chodl-negative and only rarely Nos1-positive, in contrast to the molecular profile of Sst-Chodl cells. We have corrected this error in the revised manuscript and now explicitly state that the previously reported Fos-trapped Sst cells are molecularly distinct from Sst-Chodl cells.

11. Last part of the Intro is misleading. For the last parts of the Introduction relevant to sleep, I would suggest to rewrite to better reflect current findings, specifically mentioning in the Intro previous work on sst cells, layer V pyramidal cells, REM sleep generation by the cortex, and setting the scene with the historical context, otherwise one gets a bit of a false impression that nothing else has been done in this area (sleep generation in the cortex).

We thank the reviewer for this insightful suggestion. We agree that the final section of the Introduction did not sufficiently reflect the historical and contemporary literature on cortical contributions to sleep. We have revised the text to incorporate prior work on Sst interneurons, layer V pyramidal neurons, and cortical mechanisms of REM sleep and SWS generation, thereby providing appropriate context and avoiding the impression that this area is unexplored. These additions now more accurately situate our study within the existing field.

12. The following paper should probably be discussed somewhere. Valero M et al 2021, Sleep-down state-active ID2/Nkx2.1 interneurons in the neocortex. Nat Neurosci 24 (Nos-1-positive, sst-negative cells).

We thank the reviewer for highlighting this important study. We have now incorporated the Valero et al. (2021) *Nat. Neurosci.* paper into the revised manuscript. This work identifying sleep down-state–active ID2/Nkx2.1 interneurons (Nos1-positive, Sst-negative) provides valuable context for cortical inhibitory diversity across sleep states and complements our discussion of long-range Sst-Chodl cells. We now reference this study in the Discussion to clarify how our findings relate to other interneuron populations implicated in sleep-associated cortical dynamics.

Response to reviewers

Referees' comments:

Referee #1 (Remarks to the Author):

This is a revision of an original manuscript by Ratliff, Terral and colleagues. This work main focus is the demonstration of a previously unknown role of a sparse and understudied subclass of cortical interneurons, Sst-Chodl cells, in promoting widespread cortical synchronization during non-REM sleep. The paper showcases and impressive combination of complementary experimental and analytical approaches to characterize the properties of Sst-Chold cells in behaving mice and functional role during sleep. The main finding of the paper is that Sst-Chodl cells exert widespread inhibitory modulation of cortical networks to promote synchronization and induce sleep. These exciting findings point to and important and under appreciated role of cortico-cortical inhibition in sleep regulation.

The authors have performed and outstanding job with this revision. In my original assessment I raised several issues that have been fully addressed in the revised manuscript. The authors performed several new experiments and included extensive new data in this revision. One of the new experiments is particularly remarkable as they recorded electrophisiological activity with a laminar flexible probe in the same window were 2-photon imaging was performed. This dual recording enabled new analysis of the temporal relationship between Sst-Chodl cells and cortical sleep dynamics. The analysis of this new dataset clearly demonstrates that Sst-Chodl cell activity reliably tracks NREM sleep episodes and Up-Down fluctuations within them. Other new experiments and analysis include the analysis of the activity of different cortical cell types in a large public dataset, a better characterization of the effects of optogenetic stimulation on Sst-Chodl cell activity and comparison with the effects of stimulating a different subpopulation of Sst interneurons.

Overall the new experimental and analytical evidence provided with this revision reinforced and expanded the previous results and provided strong support for the authors conclusions. I do not have more issues to raise and believe this paper will be an important contribution to the field.

We sincerely thank the reviewer for their careful evaluation, constructive suggestions, and very positive assessment of the revised manuscript. We are grateful for their recognition of the extensive new experiments and analyses included in the revision, which substantially strengthened the study. We appreciate the reviewer’s thoughtful feedback throughout the review process, which helped improve the rigor and clarity of the manuscript.

Referee #1 (Remarks on code availability):

NA

Referee #2 (Remarks to the Author):

The authors have added significant new data that solidify the main finding that Sst-Chodl GABAergic neurons are long-range projecting and that their manipulation causally promotes sleep-related cortical synchrony and behavioral state changes. In terms of novelty of the current study, previous studies have largely established correlations between Sst-Chodl neurons and sleep-related cortical states. The present work provides a causal link, offering more direct evidence that manipulating this sparse subtype can influence low-frequency synchrony and behavioral state. A study published in Neuron last year from Khodosevich's group reported similar findings, demonstrating sleep disturbances and rescue via chemogenetic manipulation of Sst-Chodl neurons (PMID: 40997796), which is conceptually aligned with the current study.

We sincerely thank the reviewer for recognizing the substantial additions made in the revised manuscript and for highlighting the complementary work from Khodosevich's group. We agree that this study is conceptually aligned with our findings and provides important complementary evidence supporting a role for Sst-Chodl neurons in the regulation of sleep-related cortical states. We have now cited and discussed this work in the revised manuscript.

Specific Comments

1. Single-cell reconstructions (Figure 1b–e)

The manuscript analyzes axon path length and projection density, but axon presence does not necessarily imply synaptic arborization. Are the quantified measures reflecting true terminal arborization or simply axonal trajectories? Given that Sst-Chodl axons travel via superficial layers, how were fibers of passage distinguished from arborization? Clear definitions of these metrics would strengthen interpretation.

We thank the reviewer for raising this important point regarding the interpretation of the morphological measurements. We agree that distinguishing terminal arborization from axons of passage is important for interpreting the long-range projection patterns of Sst-Chodl neurons. In the revised manuscript, we now clarify in the Methods which analyses quantify terminal projections (Supplementary Table 2) and which include axons of passage (Figure 1, Supplementary Table 1, and Supplementary Figures 1–2).

Given that Sst-Chodl neurons signal through both synaptic GABA release and non-synaptic neuromodulatory mechanisms, including nitric oxide and neuropeptide release, we consider both terminal arborization and long-range axonal trajectories to be biologically relevant features of these cells.

2. Contribution of long-range projections to delta synchrony (Figure 4)

The new multisite LFP recordings show that optogenetic stimulation in V1 increases low-frequency power at distant cortical sites, with strongest effects near the stimulation area. However, alternative interpretations remain possible. Light spread could contribute to stronger proximal effects. Alternatively, distal delta power changes could arise indirectly through polysynaptic corticocortical propagation rather than direct long-range inhibitory terminals. A subset of V1 neurons increases firing during stimulation, which may facilitate indirect network spread. More direct evidence would be stimulation of Sst-Chodl terminals in distal cortical regions where they innervate, or analyses that more definitively separate direct long-range synaptic effects from secondary network propagation.

We thank the reviewer for this insightful comment and for raising important alternative interpretations of the multisite LFP findings. In addition to the multisite recordings, our patch-clamp experiments (Figure 4a–e and Supplementary Figure 8) provide evidence for direct synaptic connectivity of Sst-Chodl neurons at distal cortical sites, supporting the possibility that low-frequency modulation may, at least in part, be directly regulated through long-range inhibitory projections.

At the same time, we agree that the observed increase in low-frequency power at distal cortical regions could also arise through additional mechanisms, including indirect polysynaptic corticocortical propagation potentially facilitated by increased firing in a subset of V1 neurons, as noted by the reviewer. We have now explicitly acknowledged these possibilities in the revised manuscript and clarified that the present data do not fully disentangle the relative contribution of direct long-range inhibition versus secondary network propagation.

We agree that future experiments involving selective stimulation of distal Sst-Chodl terminals will be important for further resolving these mechanisms.

Referee #3 (Remarks to the Author):

I think the authors have responded very well to all my points with extensive new data and revisions. The work is done to an impressive technical standard. The data are now much more convincing (e.g. Fig. 5, sleep induction in whole cortex DREADD experiments). In summary, this is an important study characterizing how a special subset of somatostatin-chodl cells coordinates cortical activity and can prompt wake

to sleep transitions. The work will interest both sleep physiologists and those working on cortical network function.

Happy to sign as: William Wisden

We sincerely thank the reviewer for their thoughtful and constructive comments throughout the review process. We are grateful for their recognition of the extensive new experiments and revisions included in the manuscript, which substantially strengthened the study. We appreciate their positive assessment of the revised work and its relevance to both sleep physiology and cortical network function.
