# Supplementary material for: Neocortical long-range inhibition promotes cortical synchrony and sleep
Source: Nature. 2026 Sep 9;658(8135):442–53. doi: 10.1038/s41586-026-10876-y (PMC13645642; doi:10.1038/s41586-026-10876-y)
Supplement: Supplementary file 3 [file 41586_2026_10876_MOESM4_ESM.docx]

**Supplemental Table 3**

| Area Name | Mean density | Density std. dev. |
| --- | --- | --- |
| rostrolateral area | 353.817744 | 86.8542906 |
| primary visual area | 254.230876 | 65.5825874 |
| anterolateral visual area | 190.110085 | 132.862149 |
| posteromedial visual area | 161.073969 | 106.692083 |
| lateral visual area | 101.274409 | 77.2172299 |
| anteromedial visual area | 100.422502 | 84.620492 |
| anterior area | 97.7034925 | 81.273239 |
| retrosplenial area, lateral agranular part | 49.2437828 | 98.0013612 |
| laterointermediate area | 28.0897375 | 39.5950979 |
| primary somatosensory area, barrel field | 27.8738788 | 16.9356931 |
| primary somatosensory area, trunk | 25.4189775 | 24.0517205 |
| posterior auditory area | 15.8636398 | 14.0018957 |
| posterolateral visual area | 14.947232 | 15.1223005 |
| primary somatosensory area, lower limb | 5.23046765 | 3.22956478 |
| primary auditory area | 5.19198356 | 7.34538855 |
| temporal association areas | 4.7131325 | 3.53264572 |
| retrosplenial area, dorsal part | 4.63443433 | 3.403158 |
| dorsal auditory area | 4.55820316 | 5.11929393 |
| retrosplenial area, ventral part | 3.61808834 | 3.83703651 |
| primary somatosensory area, unassigned | 3.48660635 | 3.12185105 |
| anterior cingulate area, dorsal part | 3.39150012 | 4.90557255 |
| postrhinal area | 3.3136601 | 3.48534805 |
| supplemental somatosensory area | 2.85778752 | 3.15858147 |
| primary somatosensory area, upper limb | 2.8159213 | 2.811427 |
| secondary motor area | 1.54072935 | 1.4655554 |
| primary somatosensory area, nose | 1.16144038 | 1.72629817 |
| primary motor area | 0.99224953 | 0.94206136 |
| ectorhinal area | 0.70558025 | 1.24361704 |
| visceral area | 0.4692631 | 0.69785985 |
| ventral auditory area | 0.39940397 | 0.25300502 |
| anterior cingulate area, ventral part | 0.31308763 | 0.36811857 |
| perirhinal area | 0.22011255 | 0.53916344 |
| primary somatosensory area, mouth | 0.15346161 | 0.23937919 |
| prelimbic area | 0.05074395 | 0.12429677 |
| agranular insular area, dorsal part | 0 | 0 |
| agranular insular area, posterior part | 0 | 0 |
| agranular insular area, ventral part | 0 | 0 |
| frontal pole | 0 | 0 |
| gustatory areas | 0 | 0 |
| infralimbic area | 0 | 0 |
| orbital area, lateral part | 0 | 0 |
| orbital area, medial part | 0 | 0 |
| orbital area, ventrolateral part | 0 | 0 |
