# Supplementary material for: Neocortical long-range inhibition promotes cortical synchrony and sleep
Source: Nature. 2026 Sep 9;658(8135):442–53. doi: 10.1038/s41586-026-10876-y (PMC13645642; doi:10.1038/s41586-026-10876-y)
Supplement: Supplementary file 4 [file 41586_2026_10876_MOESM5_ESM.docx]

**Supplemental Table 4**

| Area | Mean count | SD | Number of observations |
| --- | --- | --- | --- |
| Ammon's horn | 1 | 0 | 1 |
| *Anterior area/ layer* 5 | 5.23 | 4.24 | 3 |
| Anterolateral visual area/ layer 2/3 | 12 | 0 | 1 |
| Anterolateral visual area/ layer 4 | 6 | 4.25 | 4 |
| Anterolateral visual area/ layer 5 | 5 | 4.24 | 4 |
| Anterolateral visual area/ layer 6a | 2 | 0 | 2 |
| Anteromedial visual area/ layer 1 | 1 | 0 | 1 |
| Anteromedial visual area/ layer 2/3 | 2 | 3 | 2 |
| Anteromedial visual area/ layer 5 | 1 | 0 | 1 |
| Area prostriata | 1 | 0 | 1 |
| *corpus callosum/ splenium* | 1 | 0 | 1 |
| Dorsal part of the lateral geniculate complex | 3 | 0.71 | 3 |
| Dorsal part of the lateral geniculate complex/ shell | 2.2 | 1.25 | 3 |
| Entorhinal area | 2 | 0 | 1 |
| Entorhinal area/ medial part/ dorsal zone | 2 | 0 | 1 |
| *Entorhinal area/ medial* part/ dorsal zone/ layer 3 | 3 | 0 | 1 |
| Entorhinal area/ medial part/ dorsal zone/ layer 5 | 6 | 2.26 | 3 |
| Geniculate group/ dorsal thalamus | 2.5 | 0.71 | 4 |
| Inferior colliculus/ external nucleus | 1 | 0 | 1 |
| Lateral group of the dorsal thalamus | 5 | 1.99 | 3 |
| Lateral posterior nucleus of the thalamus | 6 | 4.55 | 4 |
| *Lateral visual area/* layer 2/3 | 2 | 1.41 | 3 |
| Lateral visual area/ layer 4 | 2 | 1.41 | 3 |
| Lateral visual area/ layer 5 | 1.3 | 0.58 | 3 |
| Lateral visual area/ layer 6a | 1 | 0 | 1 |
| Medial geniculate complex | 2 | 0 | 1 |
| Medial geniculate complex/ ventral part | 2 | 0 | 1 |
| optic radiation | 2 | 1.05 | 3 |
| *Posterior auditory* area/ layer 5 | 1 | 0 | 2 |
| Posterior complex of the thalamus | 2 | 0 | 1 |
| Posterior parietal association areas | 10.5 | 6.27 | 4 |
| Posterolateral visual area/ layer 4 | 1 | 0 | 1 |
| Posterolateral visual area/ layer 5 | 1 | 0 | 1 |
| posteromedial visual area/ layer 1 | 3 | 1.25 | 3 |
| posteromedial visual area/ layer 2/3 | 1 | 0 | 1 |
| *posteromedial visual area/ layer* 5 | 2 | 1.41 | 3 |
| Postrhinal area/ layer 5 | 2 | 0 | 1 |
| Primary auditory area/ layer 2/3 | 1 | 0 | 1 |
| Primary auditory area/ layer 4 | 1 | 0 | 1 |
| Primary somatosensory area/ barrel field/ layer 2/3 | 7.3 | 4.62 | 4 |
| *Primary somatosensory area/* barrel field/ layer 4 | 3 | 2.83 | 4 |
| Primary somatosensory area/ barrel field/ layer 5 | 3.5 | 1.91 | 4 |
| Primary somatosensory area/ barrel field/ layer 6b | 1 | 0 | 1 |
| Primary visual area/ layer 1 | 4.6 | 1.41 | 3 |
| Primary visual area/ layer 2/3 | 56.5 | 26.98 | 4 |
| *Primary visual* area/ layer 4 | 21.3 | 10.34 | 4 |
| Primary visual area/ layer 5 | 31.5 | 14.18 | 4 |
| Primary visual area/ layer 6a | 17.5 | 7.78 | 4 |
| Primary visual area/ layer 6b | 4 | 2.26 | 4 |
| Retrosplenial area/ dorsal part/ layer 5 | 1 | 0 | 1 |
| Retrosplenial area/ lateral agranular part/ layer 5 | 1 | 0 | 1 |
| *Retrosplenial* area/ lateral agranular part/ layer 6a | 1 | 0 | 1 |
| Retrosplenial area/ ventral part layer 3 | 1 | 0 | 1 |
| Retrosplenial area/ ventral part/ layer 5 | 1 | 0 | 1 |
| Rostrolateral area/ layer 2/3 | 29 | 12.12 | 4 |
| Rostrolateral area/ layer 4 | 11.7 | 0.58 | 4 |
| Rostrolateral area/ layer 5 | 8.3 | 4.27 | 4 |
| Rostrolateral area/ layer 6a | 9.5 | 0.71 | 3 |
| Rostrolateral area/ layer 6b | 1 | 0 | 1 |
| Somatosensory areas | 7.3 | 2.12 | 3 |
| Subiculum | 1 | 0 | 1 |
| Supplemental somatosensory area/ layer 2/3 | 3 | 2.2 | 3 |
| Supplemental somatosensory area/ layer 4 | 3 | 0 | 1 |
| Supplemental somatosensory area/ layer 5 | 1 | 0 | 1 |
| supra-callosal cerebral white matter | 1 | 0 | 1 |
| Temporal association areas/ layer 5 | 1 | 0 | 1 |
| Thalamus/ polymodal association cortex related | 2.1 | 0.96 | 4 |
| Thalamus/ sensory-motor cortex related | 2.2 | 0.71 | 4 |
